# Early molecular events associated with nitrogen deficiency in rice seedling roots

**DOI:** 10.1038/s41598-018-30632-1

**Published:** 2018-08-15

**Authors:** Ping-Han Hsieh, Chia-Cheng Kan, Hsin-Yu Wu, Hsiu-Chun Yang, Ming-Hsiun Hsieh

**Affiliations:** Institute of Plant and Microbial Biology, Academia Sinica, Taipei, 11529 Taiwan

## Abstract

Nitrogen (N) deficiency is one of the most common problems in rice. The symptoms of N deficiency are well documented, but the underlying molecular mechanisms are largely unknown in rice. Here, we studied the early molecular events associated with N starvation (−N, 1 h), focusing on amino acid analysis and identification of −N-regulated genes in rice roots. Interestingly, levels of glutamine rapidly decreased within 15 min of −N treatment, indicating that part of the N-deficient signals could be mediated by glutamine. Transcriptome analysis revealed that genes involved in metabolism, plant hormone signal transduction (e.g. abscisic acid, auxin, and jasmonate), transporter activity, and oxidative stress responses were rapidly regulated by −N. Some of the −N-regulated genes encode transcription factors, protein kinases and protein phosphatases, which may be involved in the regulation of early −N responses in rice roots. Previously, we used similar approaches to identify glutamine-, glutamate-, and ammonium nitrate-responsive genes. Comparisons of the genes induced by different forms of N with the −N-regulated genes identified here have provided a catalog of potential N regulatory genes for further dissection of the N signaling pathwys in rice.

## Introduction

Rice is a staple food for almost half of the world’s population^1^. The production of rice, especially in Asian countries, is important in food security. The Green Revolution rice cultivars developed in 1960’s, which constitute most of the rice varieties grown today, require large amounts of nitrogen (N) fertilizers to produce high yields^2^. However, the production of N fertilizer requires a lot of energy. Furthermore, only 20–30% of the applied N fertilizer is taken up by the rice plant^3,4^. Most of the N fertilizers applied to rice are lost to the air or water, which causes substantial environmental problems. Thus, the use of N fertilizer is costly to farmers and the environment. The current agricultural practices are not enconomically and environmentally sustainable. Therefore, considerable efforts have been directed toward improvement of N management and development of new rice varieties with better N use efficiency in the past decades to ensure sustainable agriculture^5–9^.

Despite decades of study, the improvement of N use efficiency in crop plants is still one of the scientific “Grand Challenges” in the 21^st^ century. To face this challenge, we need to have a better understanding of the genetics behind N uptake, transport, metabolism, and remobilization in crop plants, especially when N is limited in the environment. Since N is a major constituent of amino acids, nucleic acids, chlorophyll, ATP, coenzymes, plant hormones, and secondary metabolites, N deficiency affects all aspects of plant function, from metabolism to resource allocation, growth and development^8–10^. To cope with N deficiency, plants have evolved complex morphological, physiological, and biochemical adaptaions to the adverse environments. For instance, plants will increase its capacity to acquire N by stimulating root growth relative to shoot growth in response to N deficiency^10^. The expression of high affinifity nitrate and ammonium transporter genes was induced by N starvation (−N)^11–13^. Furthermore, the remobilization of stored N and the release of ammonium via the biosynthesis of phenylpropanoids were stimulated by N deprivation^14,15^. It is evident that plants have evolved regulatory systems to adjust metabolism, conserve resources and activate the acclimatory pathways enabling them to adapt to N-deficient conditions. Nevertheless, the molecular mechanisms underlying the N-deficient responses are still largely unknown in plants.

Global gene expression profiling using microarrays or RNA sequencing (RNA-Seq) has been a successful approach to study the molecular aspects of nutrient and stress responses. For instance, microarrays were used in several studies to identify nitrate-responsive genes in Arabidopsis and rice^16–22^. Ammonium is believed to be the major N source for paddy rice. Transcriptome analysis using microarray or RNA-Seq has been applied to identify ammonium-responsive genes in rice^23,24^. Similarly, −N-responsive genes have been identified by transcriptome analyses in rice^25–27^. These studies have provided catalogs for the identification of potential N regulatory genes.

Indeed, transcriptome analysis followed by reverse genetic study has successfully identified several N regulatory genes in plants. For instance, the *LBD/37/38/39* transcription factor genes were identified as nitrate-responsive genes in Arabidopsis^20^. Further genetic studies demonstrated that LBD37/38/39 are regulators of N responses in Arabidopsis^28^. The expression of *Os02g0325600* encoding nitrate-inducible GARP (GOLDEN2, ARR-B, Psr1) transcriptional repressor 1 (NIGT1) was specifically induced by nitrate^29^. The Arabidopsis NIGT1 homolog is involved in the integration of nitrate and phosphate signals at the root tip^30^. More recently, the *Os02g0120100* gene encoding ACT domain-containing protein kinase 1 (ACTPK1), a homolog of Arabidopsis serine/threonine/tyrosine kinase 46 (STY46), was identified by transcriptome analysis of ammonium-responsive genes in rice roots^31^. Further genetic and biochemical studies demonstrated that ACTPK1 can phosphorylate and inactivate AMT1;2, a major ammonium transporter, under ammonium-sufficient conditions^31^.

Since the availability of nutrients in the soil is directly perceived by roots, we aim to uncover the early molecular events associated with N deficiency in rice roots. This study primarily focused on the analysis of amino acids and identification of differentially expressed genes (DEGs) in response to N deficiency. All of the DEGs identified by microarray analysis were verified by quantitative (q)RT-PCR, and only the verified genes were further used for Gene Ontology (GO) and Kyoto Encyclopedia of Genes and Genomes (KEGG) pathway enrichment analyses. Interestingly, these analyses revealed that genes involved in carbon (C) and N metabolism, “plant hormone signal transduction” and “transporter activity” were enriched in −N-regulated genes. In addition, several novel N regulatory genes, including those encode transcription factors, protein kinases and protein phosphatases, were identified here. These newly identified N regulatory genes may play important roles in the regulation of N-deficient responses in rice roots.

In addition to −N, we previously used the same platform with similar criteria to identify ammonium nitrate (+N)-, glutamine (+Gln)-, and glutamate (+Glu)-regulated genes in rice roots^32–34^. These studies were conducted with similar setups, which allowed us to perform data comparisons across different experiments. We have identified at least 34 N-sensitive genes, whose expression was rapidly induced by +N and quickly repressed by −N. In addition to genes involved in nitrate/nitrite assimilation, ferredoxin reduction, and the pentose phosphate pathway, the identified N-sensitive genes include several well-known N regulatory genes, such as *LBD37* (*Os03g0445700*, *Os07g0589000*), *LBD38* (*Os03g0609500*) and *BT2* (*Os01g0908200*, BTB/POZ and TAZ domain-containing protein 2)^35^. These results implicate that some of the novel N-sensitive genes may be involved in the regulation of N metabolism and/or responses in rice. Further studies on the −N-regulated genes or N-sensitive genes identified here may provide new solutions to increase N use efficiency in rice.

## Results

### Effects of N starvation on the growth of rice seedlings

**Figure 1:**
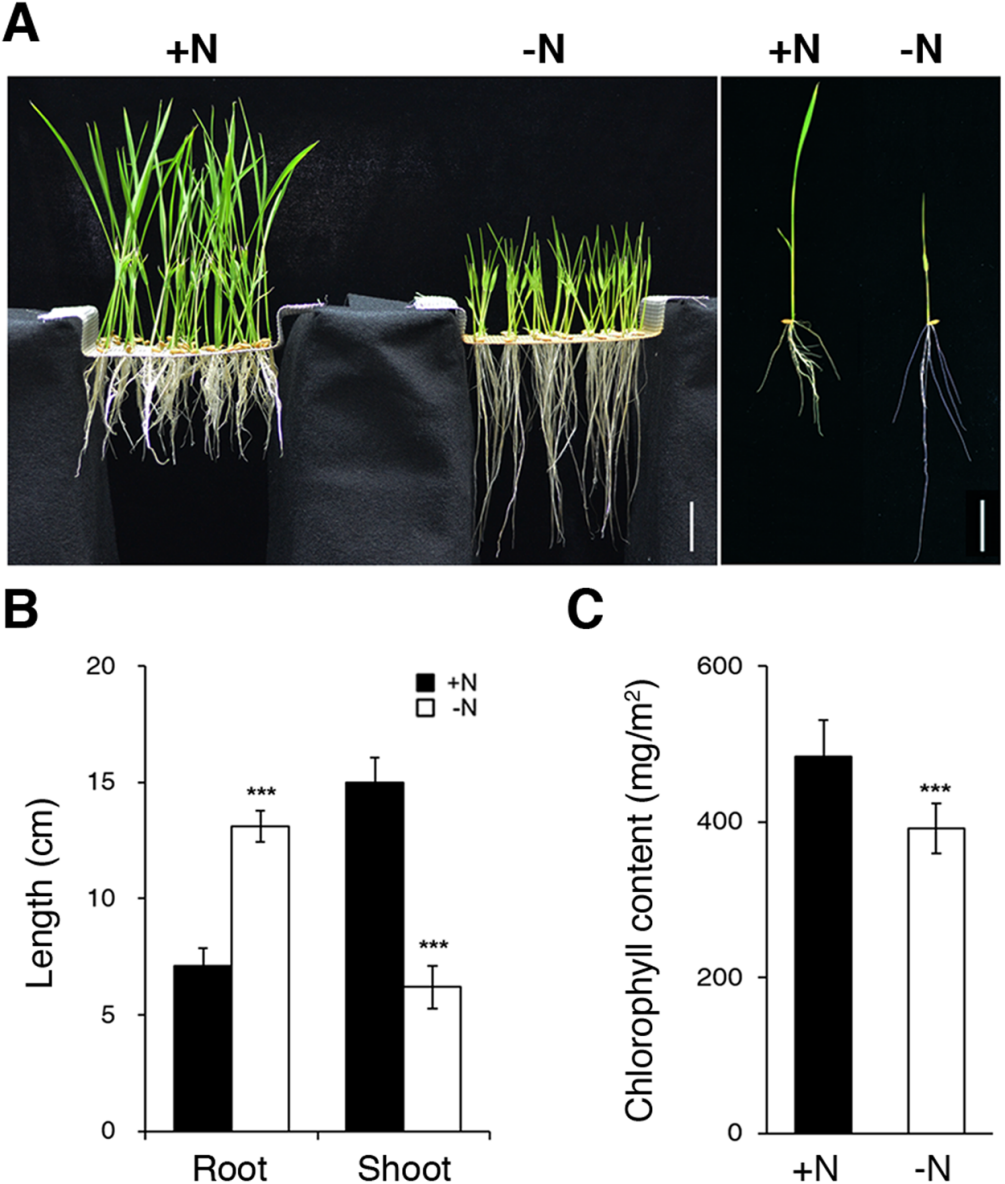
Effect of nitrogen starvation on the growth of rice seedlings. (**A**) Ten-day-old rice seedlings grown in hydroponic solutions containing 1.43 mM NH_4_NO_3_ (+N) or without nitrogen (−N). Individual plants from +N and −N were shown on the right. Root length, shoot length (**B**), and chlorophyll content (**C**) of rice seedlings from (**A**). Data are mean ±SD (*n* = 15). ***P < 0.005 represents the result of Student’s *t* test. Scale bars are 3 cm.

Compared with the green and healthy seedlings grown in +N hydroponic solution^36^, the 10-day-old rice seedlings grown in −N medium have chlorotic leaves, thinner and longer roots (Fig. 1A). The shoot length, root length, and chlorophyll content of the +N- and −N-treated rice seedlings are shown in Fig. 1B,C. The inhibition of shoot growth and promotion of root growth were also observed in 10-day-old rice seedlings treated with −N for 2, 5, and 7 days (Supplementary Fig. S1). It is known that −N can stimulate primary root growth by enhancing cell elongation as well as cell division^37^. These results implicated that N deficiency could induce changes at cellular, biochemical, and molecular levels to affect plant growth and development.

### Effects of N starvation on amino acid content in rice roots

**Figure 2:**
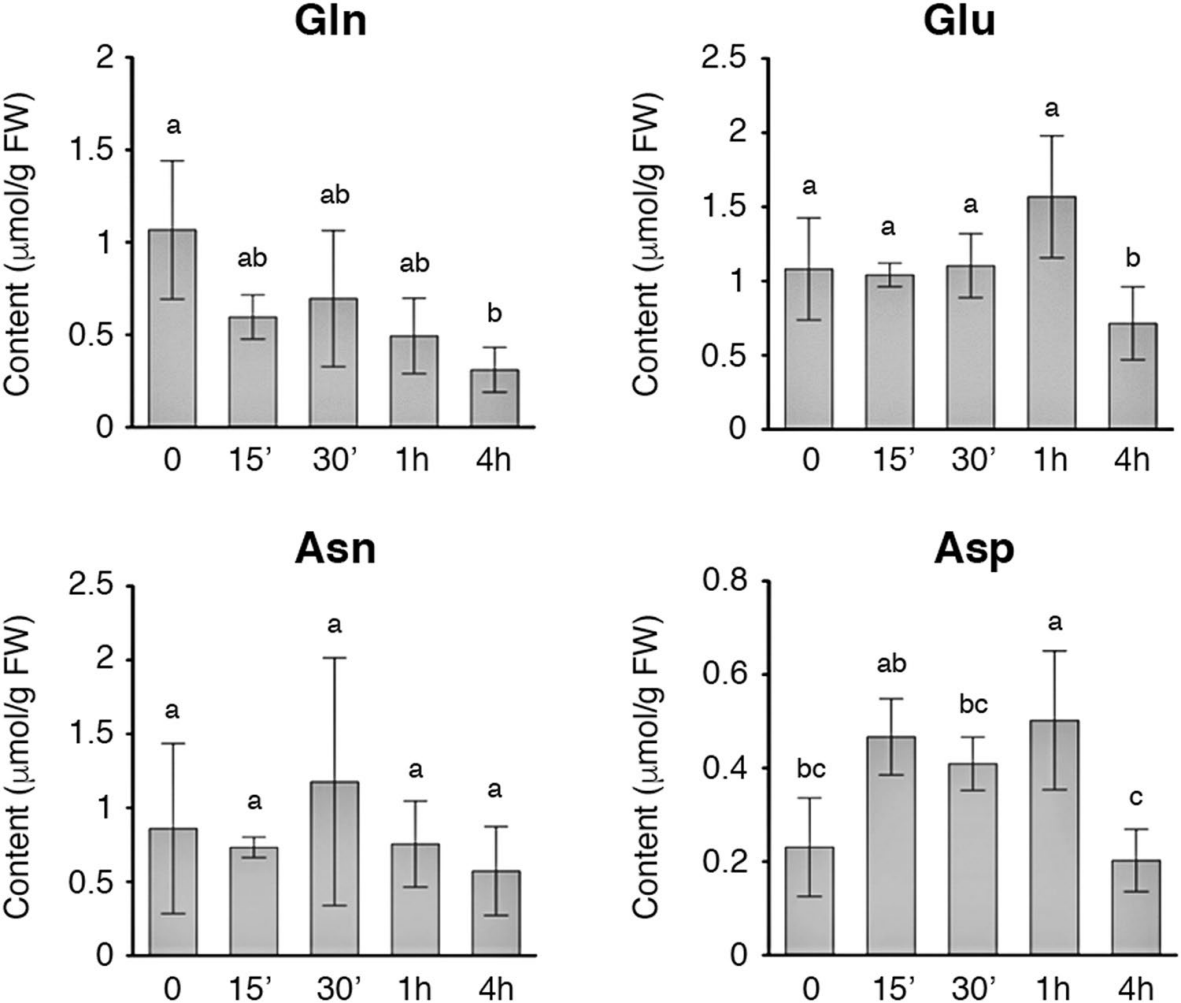
Effect of nitrogen starvation on contents of glutamine, glutamate, aspartate, and asparagine in rice roots. Amino acids extracted from roots of 10-day-old rice seedlings treated with nitrogen starvation for 0–4 h were analyzed. Data are mean ±SD (*n* = 3). Different lower-case letters indicate significant difference (ANOVA, post-hoc Tukey test, *P* < 0.05).

To examine the effects of −N on amino acid content in 10-day-old rice seedlings, we measured the amounts of free amino acids in the roots after −N treatment for 15 min to 4 h. Glutamine, glutamate, asparagine and aspartate are the most abundant amino acids in rice seedlings. Interestingly, levels of glutamine were rapidly reduced during the time course of N starvation. The amount of free glutamine was reduced approximately 50% during the first 15 min of −N treatment (Fig. 2, Gln). By contrast, levels of glutamate were not reduced until 4 h after −N treatment (Fig. 2, Glu). The amount of aspargine did not change significantly during the time course of −N treatment (Fig. 2). Levels of aspartate increased slightly within 1 h, and started to decrease after 4 h of −N treatment (Fig. 2). The amounts of the other amino acids did not change significantly during the time course of −N treatment (Supplementary Fig. S2).

### Identification of genes rapidly respond to nitrogen starvation

We used microarray analysis to identify DEGs in the roots of 10-day-old rice seedlings treated with −N for 1 h. The expression of 288 genes were differentially regulated (−N/+N, 144 up and 144 down) with 2-fold cutoff. Quantitative (q)RT-PCR analysis was used to examine the expression of all 288 genes identified by microarray analysis. The results confirmed that −N induced the expression of 116 genes and repressed the expression of 98 genes within 1 h with 2-fold cutoff. The expression patterns of these genes during the time course of −N treatment are shown in Supplementary Figs S3 and S4. The −N-induced 116 genes and the −N-repressed 98 genes are listed in Tables 1 and 2, respectively. These results revealed that approximately 81% of the up-regulated genes and 68% of the down-regulated genes identified by microarray analysis were reproducible in the independent qRT-PCR experiment. Most of the disqualified genes were up- or down-regulated by approximately 2-fold in the microarray analysis.

**Table 1:** List of genes rapidly induced by nitrogen starvation in rice roots.

No.	Locus ID		Fold change	Gene description
1	Os12g0189300	LOC_Os12g08760	9.5	Carboxyvinyl-carboxyphosphonate phosphorylmutase
2	Os12g0156100	LOC_Os12g05990	5.5	NAC domain-containing protein 90 (NAC90)
3	Os06g0725200	LOC_Os06g50950	5.5	GDSL esterase/lipase (GELP90)
4	Os08g0137800	LOC_Os08g04340	5.1	Mavicyanin, phytocyanin
5	Os07g0127500	LOC_Os07g03580	4.6	Pathogenesis-related protein PRB1-2
6	Os03g0667500	LOC_Os03g46470	4.1	Fe(II) transport protein 1 (OsIRT1)
7	Os02g0626600	LOC_Os02g41670	4.1	Phenylalanine ammonia-lyase 3 (PAL3)
8	Os09g0555500	LOC_Os09g38320	4.0	Phytoene synthase 3 (PSY3)
9	Os09g0543900	LOC_Os09g37180	3.9	Agmatine coumaroyltransferase-2
10	Os06g0587401	None	3.9	Unknown
11	Os01g0595600	LOC_Os01g41240	3.7	Probable esterase KARRIKIN- INSENSITIVE 2 (KAI2)
12	Os12g0556300	LOC_Os12g36920	3.5	Calmodulin-binding protein 60 A
13	Os03g0318400	LOC_Os03g20290	3.5	Aspartic proteinase nepenthesin-1
14	Os03g0183500	LOC_Os03g08520	3.5	Zinc-finger-FLZ domain-containing protein 24 (FLZ24)
15	Os09g0455300	LOC_Os09g28210	3.4	Basic helix-loop-helix transcription factor (bHLH120)
16	Os01g0666000	LOC_Os01g47580	3.4	Lipid phosphate phosphatase 2
17	Os07g0687900	LOC_Os07g48830	3.4	Galactinol synthase 2 (GolS2)
18	Os05g0161500	LOC_Os05g06920	3.3	GTP diphosphokinase; calcium-activated RelA-SpoT homolog 2 (CRSH2)
19	Os12g0478400	LOC_Os12g29430	3.2	Wall-associated receptor kinase 125 (WAK125)
20	Os01g0705200	LOC_Os01g50910	3.2	Late embryogenesis abundant protein, group 3
21	Os06g0521500	LOC_Os06g32990	3.1	Peroxidase 2-like
22	Os06g0218900	LOC_Os06g11520	3.0	LMBR1-like membrane protein
23	Os08g0470200	LOC_Os08g36630	3.0	Alpha carbonic anhydrase 7 (α-CA7)
24	Os07g0643700	LOC_Os07g44910	3.0	Probable carboxylesterase 18 (CXE18)
25	Os08g0353700	LOC_Os08g26520	3.0	Unknown
26	Os07g0678300	LOC_Os07g48090	3.0	CBL-interacting protein kinase 29 (CIPK29)
27	Os02g0205200	LOC_Os02g11040	3.0	Protein of unknown function (DUF642)
28	Os03g0316200	LOC_Os03g20120	2.9	Galactinol synthase 1 (GolS1)
29	Os08g0540900	LOC_Os08g42800	2.9	Unknown
30	Os07g0468100	LOC_Os07g28480	2.9	Glutathione S-transferase GSTU1
31	Os05g0135400	LOC_Os05g04490	2.9	Peroxidase 5
32	Os10g0391400	LOC_Os10g25230	2.8	TIFY 11e; jasmonate ZIM domain-containing protein 13 (JAZ13)
33	Os04g0605300	LOC_Os04g51580	2.8	Plant intracellular Ras-group-related LRR protein 1 (IRL1)
34	Os06g0522300	LOC_Os06g33100	2.8	Peroxidase 2-like
35	Os04g0308300	None	2.7	Unknown
36	Os02g0646200	LOC_Os02g43170	2.7	B-box zinc finger protein 6 (BBX6)
37	Os08g0349300	LOC_Os08g26120	2.7	Unknown
38	Os06g0142200	LOC_Os06g04990	2.7	Early nodulin 93 (ENOD93)
39	Os10g0488400	LOC_Os10g34700	2.7	Unknown, DUF761 containing protein
40	Os07g0582400	LOC_Os07g39350	2.7	Polyol/monosaccharide transporter 5 (PMT5)
41	Os04g0308401	None	2.7	Unknown, identical to Os04g0308300
42	Os01g0802700	LOC_Os01g58860	2.6	Auxin efflux carrier component 9 (PIN9)
43	Os05g0334400	LOC_Os05g26926	2.6	Chaperone protein DnaJ
44	Os12g0467700	LOC_Os12g28137	2.6	AAA-ATPase
45	Os10g0517500	LOC_Os10g37340	2.6	Methionine gamma-lyase (MGL)
46	Os03g0812400	LOC_Os03g59770	2.5	Calmodulin-like protein 2
47	Os06g0702000	LOC_Os06g48860	2.5	Auxin-responsive protein SAUR19
48	Os04g0308500	LOC_Os04g24328	2.5	23 kDa jasmonate-induced protein
49	Os08g0190100	LOC_Os08g09080	2.5	Germin-like protein 8–11
50	Os03g0180900	LOC_Os03g08320	2.5	TIFY 11c; jasmonate ZIM domain-containing protein 11 (JAZ11)
51	Os01g0895200	LOC_Os01g67010	2.5	Cytochrome b561 and DOMON domain-containing protein
52	Os04g0469100	None	2.5	Unknown
53	Os10g0523700	LOC_Os10g37980	2.5	Arogenate dehydratase/prephenate dehydratase 6 (ADT6)
54	Os08g0360300	LOC_Os08g27170	2.4	SAR DEFICIENT 1-like (SARD1)
55	Os02g0627100	LOC_Os02g41680	2.4	Phenylalanine ammonia-lyase 4 (PAL4)
56	Os08g0473900	LOC_Os08g36910	2.4	Alpha amylase isozyme 3D
57	Os07g0633400	LOC_Os07g43970	2.4	IQ domain-containing protein IQM2
58	Os04g0365100	LOC_Os04g29580	2.3	Wall-associated receptor kinase 37 (WAK37)
59	Os09g0325700	LOC_Os09g15670	2.3	Protein phosphatase 2 C 68 (PP2C68)
60	Os12g0227500	LOC_Os12g12600	2.3	Dirigent-like protein
61	Os08g0347000	LOC_Os08g25850	2.3	Unknown
62	Os12g0150200	LOC_Os12g05440	2.3	Cytochrome P450 94C1
63	Os04g0517500	LOC_Os04g43710	2.3	Phosphoenolpyruvate carboxylase kinase 3 (PPCK3)
64	Os12g0245700	LOC_Os12g14220	2.3	Unknown
65	Os01g0826400	LOC_Os01g61080	2.3	WRKY transcription factor 33 (WRKY33)
66	Os08g0352100	LOC_Os08g26350	2.3	Unknown
67	Os02g0699000	LOC_Os02g47090	2.3	NRT1/PTR FAMILY 8.3 (NPF)
68	Os04g0589800	LOC_Os04g49980	2.3	Late embryogenesis abundant (LEA) group 1
69	Os03g0860100	LOC_Os03g64260	2.3	Ethylene-responsive transcription factor 15 (ERF15)
70	Os04g0244800	LOC_Os04g17100	2.2	Heavy metal-associated isoprenylated plant protein 26
71	Os12g0518200	LOC_Os12g33300	2.2	WAT1-related protein, permease of the drug/metabolite Transporter (DMT) superfamily
72	Os09g0396900	LOC_Os09g23300	2.2	Vacuolar iron transporter 1.2
73	Os05g0332600	LOC_Os05g26840	2.2	Adenine/guanine permease AZG1
74	Os01g0736600	LOC_Os01g53500	2.2	RING-H2 finger protein ATL67
75	Os07g0461900	LOC_Os07g27780	2.2	Acetylornithine aminotransferase (ACOAT)
76	Os10g0508700	LOC_Os10g36500	2.2	21 kDa protein; PMEI-like_3; Uncharacterized subfamily of plant Invertase/pectin methylesterase inhibitor domains
77	Os09g0572700	LOC_Os09g39940	2.2	Basic blue protein; phytocyanin
78	Os10g0576600	LOC_Os10g42610	2.2	TPR protein
79	Os05g0546400	LOC_Os05g46840	2.2	Wiskott-Aldrich syndrome protein homolog
80	Os07g0599500	LOC_Os07g40850	2.2	Pollen-specific leucine-rich repeat extensin-like protein 1
81	Os03g0131100	LOC_Os03g03900	2.2	Protein NLP1
82	Os02g0581200	LOC_Os02g37070	2.2	Unknown
83	Os04g0608300	LOC_Os04g51890	2.2	Auxin-responsive protein SAUR36-like
84	Os04g0597600	LOC_Os04g50940	2.1	NRT1/PTR FAMILY 8.3 (NPF7.4)
85	Os08g0508800	LOC_Os08g39840	2.1	Lipoxygenase 7, chloroplastic; AtLOX2 homolog
86	Os04g0639000	LOC_Os04g54610	2.1	Unknown
87	Os03g0184300	LOC_Os03g08600	2.1	UDP-glucuronate:xylan alpha-glucuronosyltransferase 2
88	Os09g0484800	LOC_Os09g31120	2.1	Pirin-like protein
89	Os01g0917900	LOC_Os01g68900	2.1	C3HC4 type zinc finger protein no-on-transient A (NONA)
90	Os02g0198200	LOC_Os02g10470	2.1	Calcium-binding protein CML21
91	Os07g0561300	LOC_Os07g37400	2.1	F-box protein
92	Os03g0738600	LOC_Os03g52860	2.1	Linoleate 9S-lipoxygenase 2; AtLOX1 homolog
93	Os03g0180800	LOC_Os03g08310	2.1	TIFY 11a; jasmonate ZIM domain-containing protein 9 (JAZ9)
94	Os12g0108500	LOC_Os12g01760	2.1	F-box/LRR-repeat protein 3
95	Os09g0442100	LOC_Os09g27010	2.1	Probable receptor-like protein kinase (RLK)
96	Os01g0882800	LOC_Os01g66010	2.1	Amino acid permease 8 (AAP8)
97	Os10g0521400	LOC_Os10g37710	2.1	MhpC; Pimeloyl-ACP methyl ester carboxylesterase
98	Os08g0398300	LOC_Os08g30770	2.1	ABC transporter A family member 7
99	Os09g0511300	LOC_Os09g33650	2.1	Unknown
100	Os10g0459700	LOC_Os10g32170	2.1	Xyloglucan galactosyltransferase KATAMARI1 homolog
101	Os09g0543400	LOC_Os09g37120	2.1	Ornithine decarboxylase 1 (ODC1)
102	Os03g0194600	LOC_Os03g09880	2.1	Cytochrome b561 and DOMON domain-containing protein
103	Os08g0550200	LOC_Os08g43654	2.1	Protein DETOXIFICATION 33; MATE efflux family protein
104	Os03g0125100	LOC_Os03g03370	2.1	Beta-carotene hydroxylase 1 (BCH1); HYD3
105	Os08g0472800	LOC_Os08g36860	2.1	Abscisic acid 8′-hydroxylase 2; Cytochrome P450 707A6
106	Os11g0701100	LOC_Os11g47520	2.1	Xylanase inhibitor protein 2
107	Os01g0164100	LOC_Os01g07040	2.1	Non-classical arabinogalactan protein 30-like
108	Os07g0599300	LOC_Os07g40830	2.1	Pollen-specific leucine-rich repeat extensin-like protein 1
109	Os12g0484600	LOC_Os12g29950	2.0	Major Facilitator Superfamily (MFS) and nodulin-like domain-containing protein
110	Os08g0200100	LOC_Os08g10010	2.0	Acyl-[acyl-carrier-protein] desaturase 7, AtSAD2 homolog
111	Os01g0138500	LOC_Os01g04590	2.0	Unknown, DUF789 domain-containing
112	Os02g0143400	LOC_Os02g05060	2.0	Auxin-induced protein X15
113	Os06g0142650	LOC_Os06g05070	2.0	Probable receptor-like protein kinase (RLK)
114	Os06g0292400	LOC_Os06g18900	2.0	Unknown
115	Os04g0541700	LOC_Os04g45810	2.0	Homeobox-leucine zipper protein HOX22
116	Os05g0510100	LOC_Os05g43460	2.0	Protein LURP-one-related 5

Total RNA extracted from roots of 10-day-old rice seedlings (+N) or treated with nitrogen starvation for 1 h (−N) was used for microarray analysis. Quantitative RT-PCR analysis was used to verify the expression of genes identified in the microarray data. The expression of genes listed here was up-regulated by nitrogen starvation (−N/+N) for more than 2-fold in the qRT-PCR analysis.

**Table 2:** List of genes rapidly repressed by nitrogen starvation.

No.	Locus ID		Fold change	Gene description
1	Os02g0770800	LOC_Os02g53130	−16.7	Nitrate reductase [NAD(P)H]
2	Os05g0114400	LOC_Os05g02390	−13	Zinc finger transcription factor, ZOS5-02
3	Os08g0468100	LOC_Os08g36480	−7.5	Nitrate reductase [NADH] 1
4	Os11g0184900	LOC_Os11g08210	−7.4	NAC domain-containing protein 5 (NAC5)
5	Os01g0631200	LOC_Os01g44050	−7	Uroporphyrinogen-III C-methyltransferase
6	Os03g0684700	LOC_Os03g48030	−6.8	Unknown, integral membrane HPP family protein
7	Os08g0120600	LOC_Os08g02700	−6.4	Fructose-bisphosphate aldolase
8	Os05g0194900	LOC_Os05g10650	−6.2	ATP-dependent 6-phosphofructokinase 4 (PFK04)
9	Os03g0609500	LOC_Os03g41330	−6.1	LOB domain-containing protein 38 (LBD38)
10	Os09g0482800	LOC_Os09g30490	−5.9	EF-hand domain-containing protein
11	Os03g0126900	LOC_Os03g03520	−5.8	Unknown, putative AtpZ domain-containing protein
12	Os08g0113900	LOC_Os08g02200	−5.7	Unknown, putative AtpZ domain-containing protein
13	Os01g0860601	LOC_Os01g64120	−5.6	Ferredoxin, root R-B1
14	Os04g0506800	LOC_Os04g42760	−5	Sialyltransferase-like protein 3 (STLP3)
15	Os09g0484900	LOC_Os09g31130	−5	Tonoplast dicarboxylate transporter (TDT)
16	Os07g0589000	LOC_Os07g40000	−4.8	LOB domain-containing protein 37 (LBD37)
17	Os12g0198900	LOC_Os12g09710	−4.6	NB-ARC and LRR domain-containing protein, RPM1-like
18	Os04g0665600	LOC_Os04g56990	−4.5	Myb family protein
19	Os07g0147500	LOC_Os07g05360	−4.5	Photosystem II 10 kDa polypeptide; PsbR
20	Os05g0119000	LOC_Os05g02750	−4.1	UPF0014 membrane protein STAR2; AtALS3 homolog
21	Os05g0360400	LOC_Os05g29710	−4	RING-type E3 ubiquitin-protein ligase EL5-like
22	Os09g0545280	LOC_Os09g37330	−3.8	OsSAUR39 - Auxin-responsive SAUR gene family member
23	Os01g0179600	LOC_Os01g08440	−3.8	Crocetin glucosyltransferase; AtIAGLU homolog
24	Os09g0433800	LOC_Os09g26370	−3.7	Zinc-finger-FLZ domain-containing protein 14 (FLZ14)
25	Os02g0620600	LOC_Os02g40730	−3.6	Ammonium transporter 1 member 2 (AMT1;2)
26	Os02g0756600	LOC_Os02g52000	−3.5	Protein EXORDIUM
27	Os02g0120100	LOC_Os02g02780	−3.5	Serine/threonine-protein kinase STY46
28	Os02g0325600	LOC_Os02g22020	−3.5	Nitrate-inducible, GARP-type transcriptional repressor 1 (NIGT1)
29	Os12g0113500	LOC_Os12g02200	−3.5	CBL-interacting protein kinase 14 (CIPK14)
30	Os10g0578800	LOC_Os10g42780	−3.3	Plastidal glycolate/glycerate translocator 1 (PLGG1)
31	Os01g0908200	LOC_Os01g68020	−3.3	BTB/POZ and TAZ domain-containing protein 2 (BT2)
32	Os05g0111800	LOC_Os05g02110	−3.3	Protein phosphatase 2 C 46 (PP2C46)
33	Os11g0305400	LOC_Os11g20040	−3.1	O-methyltransferase (OMT)
34	Os03g0764600	LOC_Os03g55590	−3.1	MYB family protein
35	Os05g0401500	LOC_Os05g33310	−3	2OG-Fe(II) oxygenase superfamily
36	Os05g0443700	LOC_Os05g37150	−3	Unknown, syntaxin 6 N-terminal domain-containing protein
37	Os05g0472400	LOC_Os05g39540	−3	Zinc transporter 9 (ZIP9)
38	Os04g0280500	LOC_Os04g21130	−2.9	Putative F-box protein PP2-B12
39	Os05g0506800	LOC_Os05g43120	−2.9	GDSL esterase/lipase 72 (GELP72)
40	Os06g0535200	LOC_Os06g34430	−2.9	RING-H2 finger protein ATL74
41	Os06g0566300	LOC_Os06g37010	−2.9	Zinc transporter 10 (ZIP10)
42	Os05g0380250	None	−2.9	Unknown
43	Os01g0208700	LOC_Os01g11054	−2.9	Phosphoenolpyruvate carboxylase 4 (PPC4), chloroplastic
44	Os07g0686300	LOC_Os07g48680	−2.9	RING-H2 finger protein ATL45
45	Os02g0214900	LOC_Os02g12350	−2.8	Histone deacetylase 3 (HDAC3)
46	Os01g0179800	LOC_Os01g08460	−2.8	Probable serine incorporator (Serinc)
47	Os03g0784700	LOC_Os03g57120	−2.8	Ferredoxin−NADP reductase (FNR)
48	Os03g0445700	LOC_Os03g33090	−2.7	LOB domain-containing protein 37 (LBD37)
49	Os04g0649500	LOC_Os04g55600	−2.7	Unknown
50	Os04g0649600	LOC_Os04g55610	−2.7	Unknown
51	Os04g0640900	LOC_Os04g54830	−2.7	Unknown
52	Os04g0475600	LOC_Os04g39980	−2.7	Dioxygenase for auxin oxidation (DAO)
53	Os12g0204100	LOC_Os12g10280	−2.7	Aquaporin nodulin 26-like intrinsic membrane protein NIP3;5
54	Os02g0807000	LOC_Os02g56310	−2.6	Phosphoenolpyruvate carboxylase kinase 1 (PPCK1)
55	Os04g0520700	LOC_Os04g43990	−2.6	Senescence regulator-like protein
56	Os06g0633100	LOC_Os06g42660	−2.6	Glutamine dumper 6 (GDU6)
57	Os06g0683800	LOC_Os06g46980	−2.6	Unknown
58	Os05g0472700	LOC_Os05g39560	−2.6	Zinc transporter 5 (ZIP5)
59	Os03g0823400	LOC_Os03g60840	−2.6	Bowman-Birk type trypsin inhibitor (BBTI)
60	Os02g0765600	LOC_Os02g52710	−2.6	Alpha-amylase 1 (AMY1)
61	Os05g0501600	LOC_Os05g42220	−2.6	Leucine rich repeat domain-containing protein
62	Os01g0803300	LOC_Os01g58910	−2.5	EamA domain-containig drug/metabolite transporter (DMT)
63	Os01g0383100	LOC_Os01g28600	−2.5	Exocyst complex component EXO70A1
64	Os11g0484500	LOC_Os11g29400	−2.4	6-phosphogluconate dehydrogenase (6PGDH)
65	Os11g0256900	LOC_Os11g15040	−2.4	O-methyltransferase
66	Os06g0692600	LOC_Os06g47750	−2.4	Tyrosine-sulfated glycopeptide receptor 1; leucine-rich repeat receptor-like protein kinase
67	Os01g0357100	LOC_Os01g25484	−2.4	Nitrite reductase
68	Os05g0443500	LOC_Os05g37140	−2.4	Ferredoxin-6, chloroplastic
69	Os04g0683700	LOC_Os04g58710	−2.4	Oxalate–CoA ligase; acyl-activating enzyme 3 (AAE3)
70	Os03g0599000	LOC_Os03g40194	−2.4	Putative disease resistance protein RGA3
71	Os04g0561500	LOC_Os04g47360	−2.4	Prolyl endopeptidase
72	Os07g0406300	LOC_Os07g22350	−2.3	Glucose-6-phosphate dehydrogenase (G6PDH)
73	Os04g0403701	LOC_Os04g33080	−2.3	Protein phosphatase 2 C 39 (PP2C39)
74	Os01g0621900	LOC_Os01g43370	−2.3	Unknown, conserved peptide uORF-containing transcript
75	Os03g0190300	LOC_Os03g09070	−2.3	Leucine rich repeat domain-containing protein
76	Os08g0207500	LOC_Os08g10630	−2.3	Zinc transporter 4 (ZIP4)
77	Os08g0465700	LOC_Os08g36310	−2.2	Cytochrome P450 76M5-like
78	Os03g0838900	LOC_Os03g62240	−2.2	Unknown, mTERF domain-containing protein
79	Os04g0165200	LOC_Os04g08290	−2.2	Zinc finger protein STAR3-like, ZOS4-04
80	Os09g0453300	LOC_Os09g27990	−2.2	Annexin D8
81	Os02g0756200	LOC_Os02g51970	−2.1	Protein EXORDIUM
82	Os03g0854000	LOC_Os03g63700	−2.1	Putative gamma-glutamylcyclotransferase
83	Os01g0355100	LOC_Os01g25270	−2.1	Jacalin-like plant lectin domain-containing protein
84	Os10g0554200	LOC_Os10g40600	−2.1	NRT1/ PTR FAMILY 6.3 (NPF6.5)
85	Os10g0328400	LOC_Os10g18099	−2.1	Unknown
86	Os06g0323100	LOC_Os06g21820	−2.1	Indole-3-acetate O-methyltransferase 1 (IAMT1)-like
87	Os07g0119300	LOC_Os07g02800	−2.1	MYB family protein
88	Os02g0306401	LOC_Os02g20360	−2.1	Nicotianamine aminotransferase A
89	Os09g0474000	LOC_Os09g29820	−2.1	bZIP transcription factor 53
90	Os03g0243100	LOC_Os03g13950	−2.1	Actin-depolymerizing factor 5 (ADF5)
91	Os01g0747300	LOC_Os01g54340	−2.1	PDDEXK nuclease-like family of unknown function
92	Os02g0525100	LOC_Os02g32450	−2.1	Unknown
93	Os01g0191700	LOC_Os01g09570	−2.1	ATP-dependent 6-phosphofructokinase 6 (PFK01)
94	Os03g0228100	LOC_Os03g12690	−2	Unknown
95	Os05g0342000	LOC_Os05g27580	−2	Wound-induced WI12 family protein
96	Os01g0888900	LOC_Os01g66544	−2	Unknown
97	Os04g0645500	LOC_Os04g55250	−2	S-adenosylmethionine-dependent methyltransferase
98	Os05g0411100	LOC_Os05g34030	−2	NRT1/PTR FAMILY 3.1-like (NPF)

Total RNA extracted from roots of 10-day-old rice seedlings (+N) or treated with nitrogen starvation for 1 h (−N) was used for microarray analysis. Quantitative RT-PCR analysis was used to verify the expression of genes identified in the microarray data. The expression of genes listed here was down-regulated by nitrogen starvation (−N/+N) for more than 2-fold in the qRT-PCR analysis.

### GO and KEGG enrichment analyses of early N starvation-induced genes

**Figure 3:**
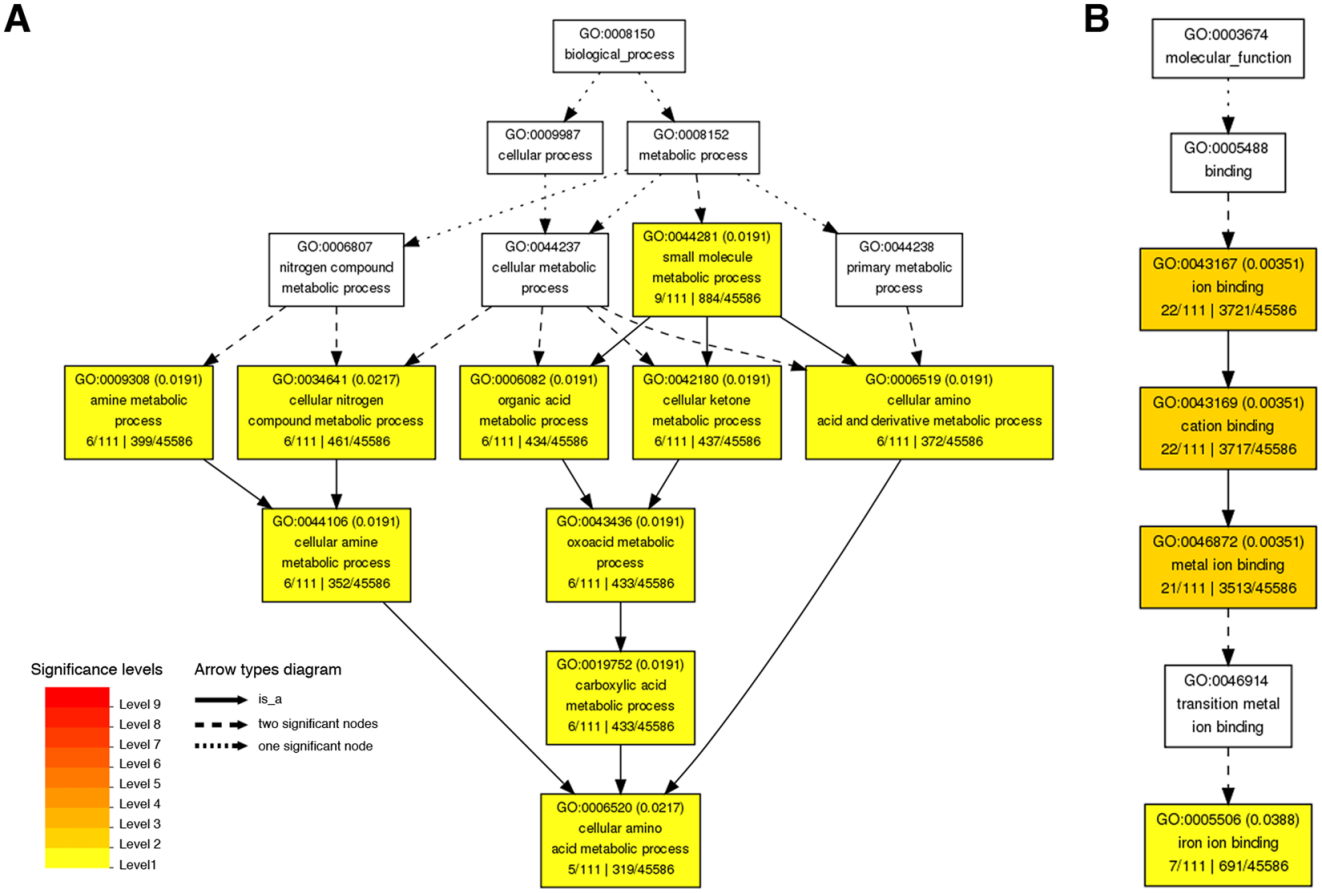
Gene ontology (GO) analysis of nitrogen starvation (−N)-induced genes. AgriGO (http://bioinfo.cau.edu.cn/agriGO/) was used to analyze the −N-induced genes in 10-day-old rice seedling roots (false discovery rate, FDR < 0.05). Of the three structured networks, the −N-induced genes are significantly enriched in the GO categories of biological process (**A**), and molecular function (**B**). The −N-induced genes were not enriched in the GO categories of cellular component.

AgriGO (http://bioinfo.cau.edu.cn/agriGO/) was used for GO enrichment analysis of the 116 genes induced by −N. In biological process, the GO terms “cellular amino acid and derivative metabolic process”, “cellular ketone metabolic process”, “organic acid metabolic process”, “cellular nitrogen compound metabolic process”, and “amine metabolic process” were significantly enriched (Fig. 3A). In molecular function, the GO terms “ion/cation/metal ion binding” were significantly enriched (Fig. 3B). No GO terms were enriched in the category of cellular component. The information of genes enriched in GO analysis is provided in Supplementary Table S1. In addition, KEGG pathway analysis of the 116 −N-induced genes revealed that “plant hormone signal transduction (ko04075)”, “carotenoid biosynthesis (ko00906)”, “plant-pathogen interaction (ko04626)”, “linoleic acid metabolism (ko00591)”, and “arginine and proline metabolism (ko00330)” were enriched. The information of genes enriched in these KEGG pathways is provided in Supplementary Table S2. Together, the GO and KEGG enrichment analyses suggest that −N rapidly induced the expression of genes involved in N remobilization and plant hormone signal transduction in rice roots.

### Analysis of −N-induced genes involved in metabolism, plant hormone signal transduction, and oxidative response

**Figure 4:**
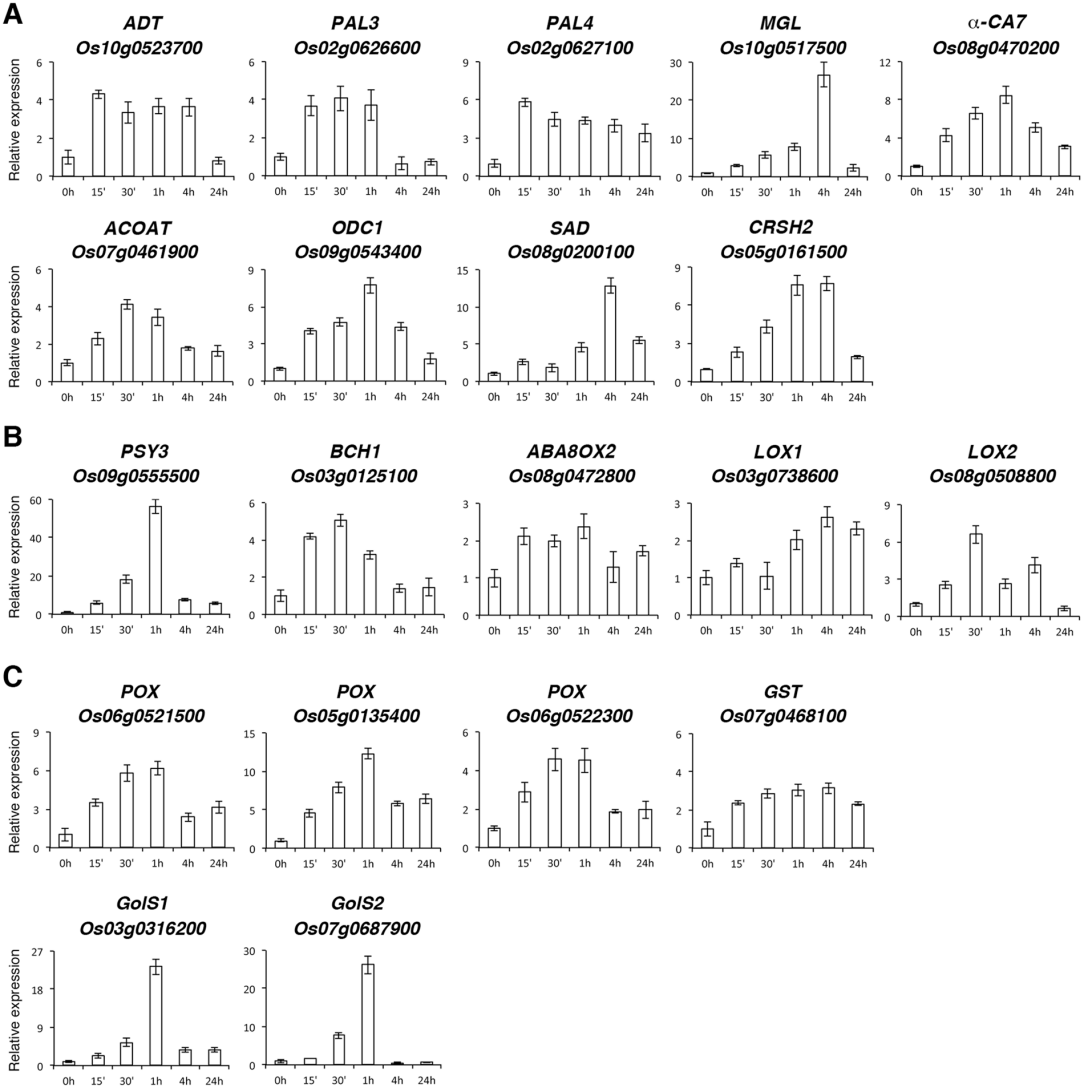
Expression of metabolic genes rapidly induced by nitrogen starvation. Quantitative RT-PCR analysis of genes involved in small molecule metabolic process (**A**), abscisic acid and jasmonic acid metabolism (**B**), and oxidative stress response (**C**). RNA samples from roots of 10-day-old rice seedlings treated with nitrogen starvation for 0, 15 min, 30 min, 1 h, 4 h and 24 h were analyzed by qRT-PCR. The expression level of each gene in the control sample (0 h) was set at 1. Relative expression represents the fold change of the target gene relative to that of the control. Data are mean ±SD of 3 biological replicates.

In the category of biological process, GO enrichment analysis identified 9 genes encoding enzymes involved in metabolic processes. These enzymes are arogenate dehydratase/prephenate dehydratase 6 (ADT6, Os10g0523700) of phenylalanine biosynthesis, phenylalanine ammonia-lyase (PAL3, Os02g0626600 and PAL4, Os02g0627100) of the phenylpropanoid pathway, methionine gamma-lyase (MGL, Os10g0517500) of methionine catabolism, acetylornithine aminotransferase (ACOAT, Os07g0461900) and ornithine decarboxylase 1 (ODC1, Os09g0543400) of arginine biosynthesis, acyl-[acyl-carrier-protein] desaturase 7 (SAD, Os08g0200100, an Arabidopsis SAD2 homolog), α-carbonic anhydrase 7 (α-CA7, Os08g0470200), and GTP diphosphokinase (calcium-activated RelA-SpoT homolog 2 [CRSH2], Os05g0161500). CRSH2 is a Ca^+2^-activated (p)ppGpp synthetase that has been proposed to integrate the Ca^+2^ and (p)ppGpp signaling pathways in rice^38^. The expression patterns of these metabolic genes during the time course (0–24 h) of −N treatment are shown in Fig. 4A.

The genes identified in the KEGG pathway “carotenoid biosynthesis (ko00906)”, *Os09g0555500* encoding phytoene synthase 3 (PSY3), *Os03g0125100* encoding beta-carotene hydroxylase 1 (BCH1), and *Os08g0472800* encoding abscisic acid 8′-hydroxylase 2 (ABA8OX2), are also involved in the biosynthesis and metabolism of plant hormone ABA^39–42^. The expression patterns of these genes during the time course of −N treatment are shown in Fig. 4B. The genes enriched in the KEGG pathway “linoleic acid metabolism (ko00591), including *Os08g0508800* encoding chloroplastic lipoxygenase 7, a homolog of Arabidopsis LOX2, and *Os03g0738600* encoding linoleate 9S-lipoxygenase 2, a homolog of Arabidopsis LOX1, are involved in the biosynthesis of plant hormone jasmonic acid (JA). The expression of these JA biosynthesis genes was rapidly induced by −N (Fig. 4B).

In addition, the expression of several genes related to oxidative stress was also rapidly induced by −N (Table 1 and Supplementary Fig. S3). For instance, the expression of *Os06g0521500*, *Os05g0135400*, and *Os06g0522300* encoding peroxidase (POX) and *Os07g0468100* encoding glutathione S-transferase (GSTU1) was rapidly induced by −N (Fig. 4C). Galactinol synthase (GolS) is the key enzyme for the synthesis of raffinose family oligosaccharide in plants^43^. In addition to their roles as osmoprotectants, galactinol and raffinose have been shown to protect plants from oxidative damage^44^. Interestingly, we found that the expression of *GolS1* (*Os03g0316200*) and *GolS2* (*Os07g0687900*) was rapidly and strongly induced by −N in rice roots (Fig. 4C). These results implicated that oxidative stress might be one of the early events associated with N deprivation in the roots of rice seedlings.

### Identifiction of early N starvation-induced transcription factor genes

**Figure 5:**
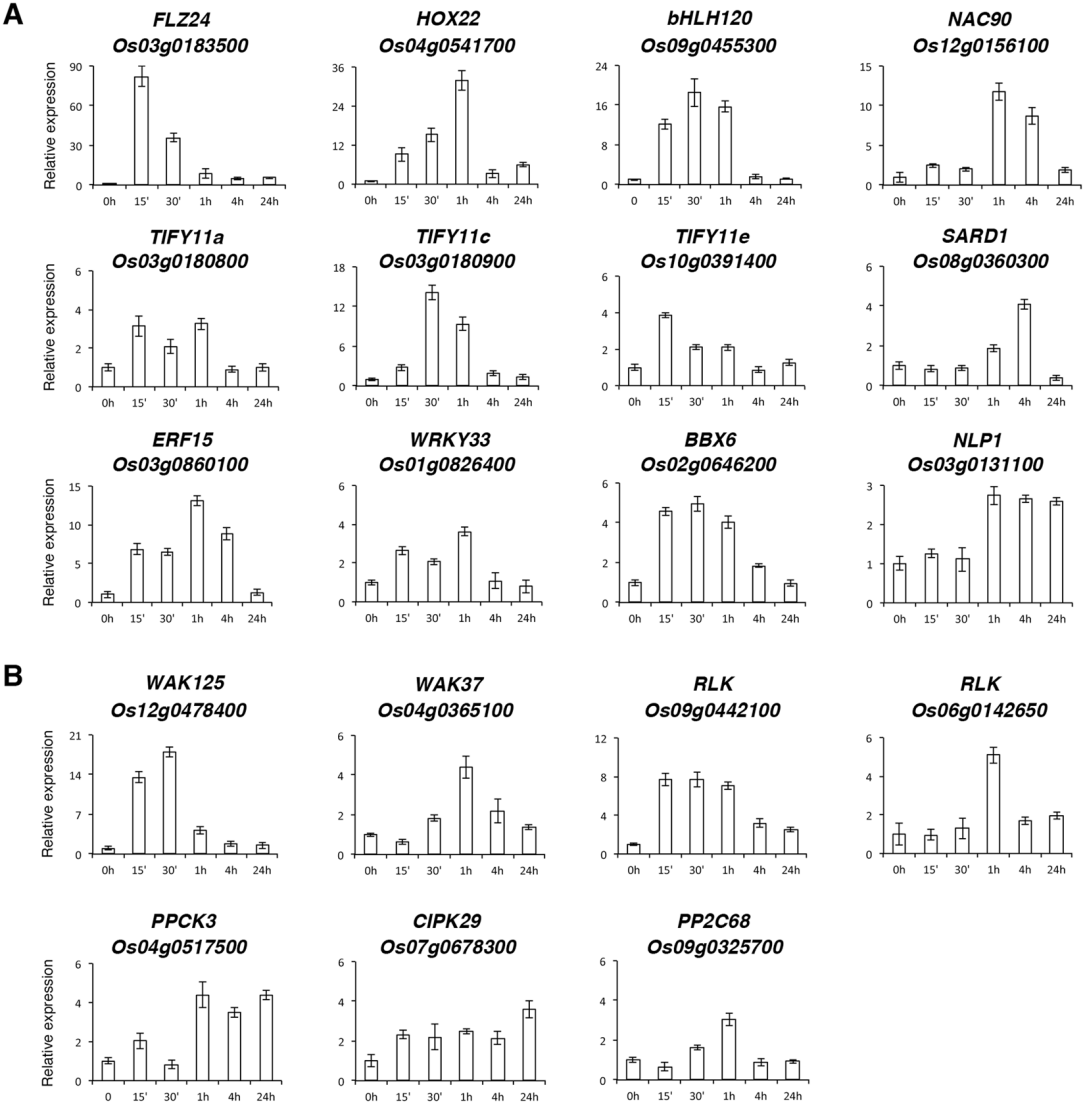
Expression of transcription factor and protein kinase/phosphatase genes induced by nitrogen starvation. (**A**) Transcription factor genes. (**B**) Protein kinase/phosphatase genes. RNA samples from roots of 10-day-old rice seedlings treated with nitrogen starvation for 0, 15 min, 30 min, 1 h, 4 h and 24 h were analyzed by qRT-PCR. The expression level of each gene in the control sample (0 h) was set at 1. Relative expression represents the fold change of the target gene relative to that of the control. Data are mean ±SD of 3 biological replicates.

Of the 116 −N-induced genes, at least 12 genes encode transcription factors. The expression patterns of these transcription factor genes during the time course of −N treatment are shown in Fig. 5A. The *Os03g0183500* gene encodes an uncharacterized plant-specific FCS-like zinc finger protein FLZ24^45,46^. The expression of *FLZ24* was rapidly and strongly induced by −N, which peaked at 15 min during the time course of −N treatment (Fig. 5A). The homeodomain-leucine zipper (HD-ZIP) gene *HOX22* (*Os04g0541700*) has been shown to affect ABA biosynthesis and regulate drought and salt responses through ABA-mediated signaling pathways in rice^47^. The basic helix-loop-helix transcription factor gene *bHLH120* (*Os09g0455300*) corresponds to the quantitative trait locus *qRT9* that controls root thickness and root length in upland rice^48^. Previous studies revealed that the expression of *HOX22* and *bHLH120* was strongly induced by polyethylene glycol, salt, and ABA^47,48^. Interestingly, the expression of *HOX22* and *bHLH120* was also rapidly and strongly induced by −N (Fig. 5A). The *Os12g0156100* gene encodes a homolog of Arabidopsis NAC90. The expression of *Os12g0156100* (*NAC90*) was strongly induced by −N after treatment for 1–4 h (Fig. 5A).

The plant-specific TIFY/JAZ (jasmonate-zim domain) transcription factors are key regulators of JA signaling pathways^49^. It has been demonstrated that the expression of *TIFY11a* (*JAZ9*, *Os03g0180800*), *TIFY11c* (*JAZ11*, *Os03g0180900*) and *TIFY11e* (*JAZ13*, *Os10g0391400*) was strongly induced by JA^50^. Interestingly, −N also rapidly induced the expression of these key transcription factor genes for JA signaling (Table 1). The expression patterns of *TIFY11a* (*JAZ9*, *Os03g0180800*), *TIFY11c* (*JAZ11*, *Os03g0180900*) and *TIFY11e* (*JAZ13*, *Os10g0391400*) during the time course of −N treatment are shown in Fig. 5A. The *Os08g0360300* gene encodes a homolog of Arabidopsis SYSTEMIC ACQUIRED RESISTANCE DEFICIENT 1 (SARD1) that is involved in salicylic acid (SA) signaling pathways. The expression of *SARD1* (*Os08g0360300*) was also induced by −N in rice roots (Fig. 5A).

The *Os03g0860100* gene encodes ethylene-responsive transcription factor 15 (ERF15) of unknown function. The expression of *Os03g0860100* (*ERF15*) was rapidly and strongly induced by −N (Fig. 5A). The *Os01g0826400* gene encodes a homolog of Arabidopsis WRKY33 that plays an important role in defense response^51^. The expression of rice *WRKY33* (*Os01g0826400*) was also induced by −N (Fig. 5A). The *Os02g0646200* gene encodes B-box zinc finger protein 6 (BBX6), a homolog of Arabidopsis BBX20/21 that are involved in photomorphogenesis^52,53^. The expression of *Os02g0646200* (*BBX6*) was rapidly induced by −N (Fig. 5A). The Arabidopsis NIN-like proteins (NLPs) are key players in nitrate signaling pathways^54^. The *Os03g0131100* (*NLP1*) gene encodes a homolog of Arabidopsis NLPs. The expression of *NLP1* was rapidly induced by −N in rice roots (Fig. 5A).

### Identifiction of early N starvation-induced protein kinase/phosphatase genes

Protien kinases and phosphatases are well known regulatory proteins involved in various signal transduction pathways. We have identified at least 6 protein kinase and one protein phosphatase genes that are rapidly induced by −N in rice roots (Table 1). The cell wall-associated receptor kinases (WAKs) are primarily involved in the regulation of plant cell wall functions such as pathogen response, binding to pectin to control cell expansion, morphogenesis and development^55,56^. The expression of *WAK125* (*Os12g0478400*) and *WAK37* (*Os04g0365100*) was rapidly induced by −N in rice roots (Fig. 5B). Interestingly, *WAK125* was previously found to be an early glutamate-responsive gene^33^. In addition to *WAK125* and *WAK37*, the expression of *Os09g0442100* and *Os06g0142650*, encoding receptor-like protein kinase (RLK) homologs, was also rapidly induced by −N (Fig. 5B). The functions of these protein kinases have yet to be characterized in rice.

The activity of phosphoenolpyruvate carboxylase (PEPC), a key enzyme of primary metabolism of higher plants, is regulated by PEPC kinase (PPCK). The expression of *PPCK3* (*Os04g0517500*) was rapidly induced by −N in rice roots (Fig. 5B). The expression of *Os07g0678300* encoding calcineurin B-like protein (CBL) interacting protein kinase 29 (CIPK29) was rapidly induced by −N (Fig. 5B). Interestingly, the expression of *CIPK29* was previously shown to be down-regulated by potassium (K) deficiency^57,58^. In addition, the *Os12g0189300* gene encoding carboxyvinyl-carboxyphosphonate phosphorylmutase was rapidly and strongly induced by −N (Table 1 and Supplementary Fig. 3), but was also previously shown to be down-regulated by K deficiency^57,58^. These genes may be involved in the regulation of −N and −K responses, but in the opposite way.

The only protein phosphatase gene found to be rapidly induced by −N is *Os09g0325700* that encodes protein phosphatase 2 C 68 (PP2C68). The rice PP2C68 is a homolog of Arabidopsis HAI1/2/3 (highly ABA-induced PP2C protein 1/2/3). It is not clear if ABA can induce the expression of *PP2C68*. Nevertheless, −N can induce the expression of *PP2C68* in rice roots. The expression patterns of *PP2C68* during the time course of −N treatment are shown in Fig. 5B.

### Analysis of genes rapidly induced by −N and +N

**Figure 6:**
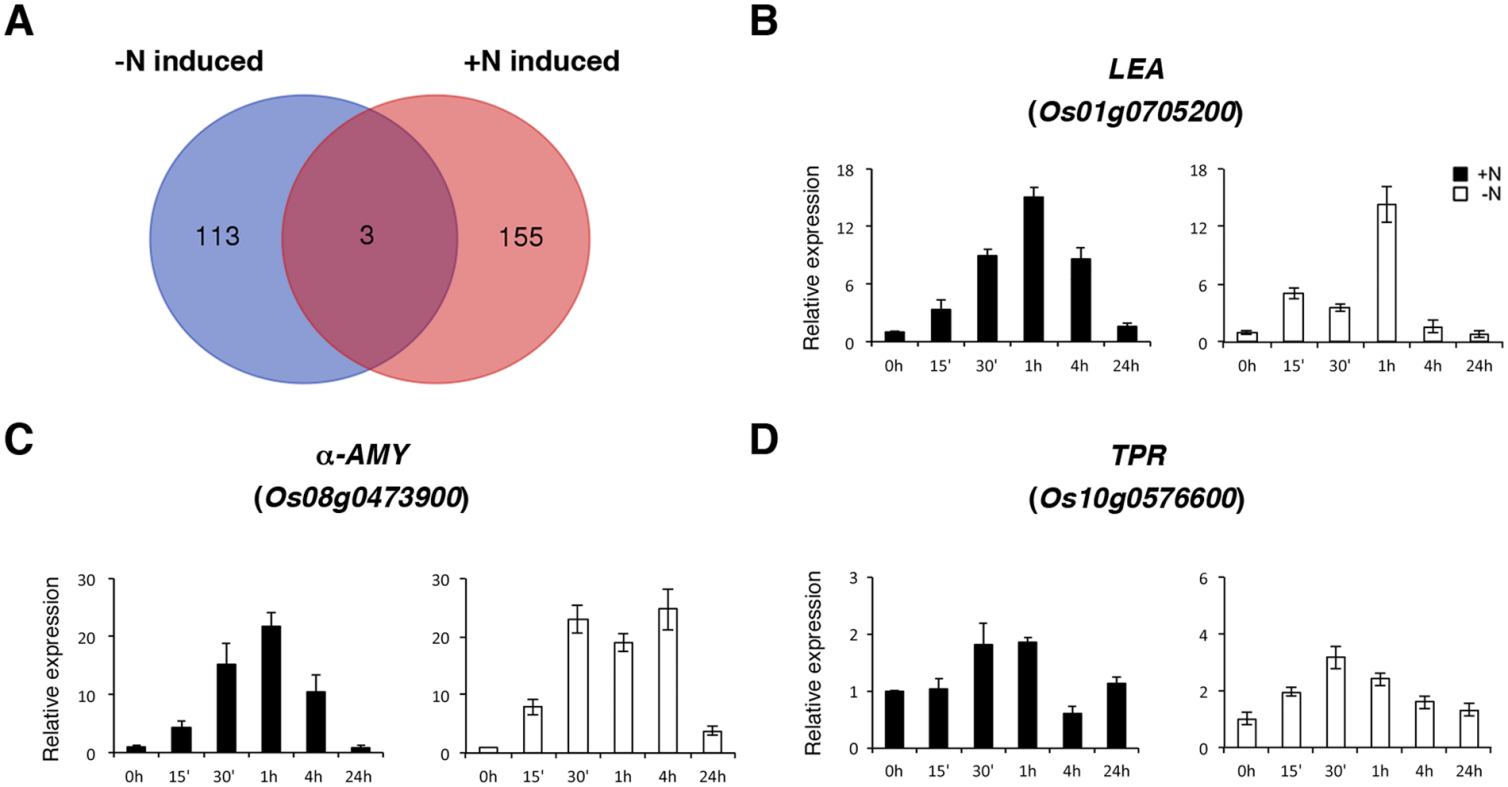
Identification of genes rapidly induced by the addition and deprivation of nitrogen. (**A**) Venn diagram of genes induced by nitrogen starvation (−N) and ammonium nitrate supplementation (+N)^34^. RNA samples from roots of 10-day-old rice seedlings treated with −N or +N for 0, 15 min, 30 min, 1 h, 4 h and 24 h were used for qRT-PCR to analyze the expression of *Os01g0705200* encoding a late embryogenesis abundant protein (**B**), *Os08g0473900* encoding α-amylase isozyme 3D (**C**), and *Os10g0576600* encoding a TPR protein (**D**). The expression level of each gene in the control sample (0 h) was set at 1. Relative expression represents the fold change of the target gene relative to that of the control. Data are mean ±SD of 3 biological replicates.

We previously used microarray and qRT-PCR analyses to identify 158 genes that were rapidly induced by +N (1.43 mM ammonium nitrate, 30 min) in the roots of hydroponically grown rice seedlings^34^. We compared the 116 genes up-regulated by −N (Table 1) with the 158 genes induced by +N and found that the expression of 3 genes, *Os01g0705200* encoding a late embryogenesis abundant protein, *Os08g0473900* encoding an α-amylase, and *Os10g0576600* encoding a tetratricopeptide repeat (TPR) protein was induced by both −N and +N treatments in rice roots (Fig. 6A and Table 3). To verify this result, we used 10-day-old rice seedlings to conduct −N and +N time course treatments. Total RNA extracted from roots of these samples was used for qRT-PCR analysis to examine the expression of *Os01g0705200*, *Os08g0473900*, and *Os10g0576600*. The results revealed that these genes responded to +N and −N rapidly and transiently (Fig. 6B–D). In general, the expression of these genes was induced by +N and −N after 15 min to 4 h, and back to control levels after 24 h (Fig. 6B–D).

**Table 3:** List of genes rapidly induced by nitrogen starvation and ammonium nitrate in rice roots.

Locus ID		Fold change		Gene description
		−N/+N	+N/−N	
Os01g0705200	LOC_Os01g50910	3.2	3.0	Late embryogenesis abundant protein, group 3
Os08g0473900	LOC_Os08g36910	2.4	2.7	Alpha amylase isozyme 3D
Os10g0576600	LOC_Os10g42610	2.2	2.1	TPR protein

### GO and KEGG enrichment analyses of early N starvation-repressed genes

**Figure 7:**
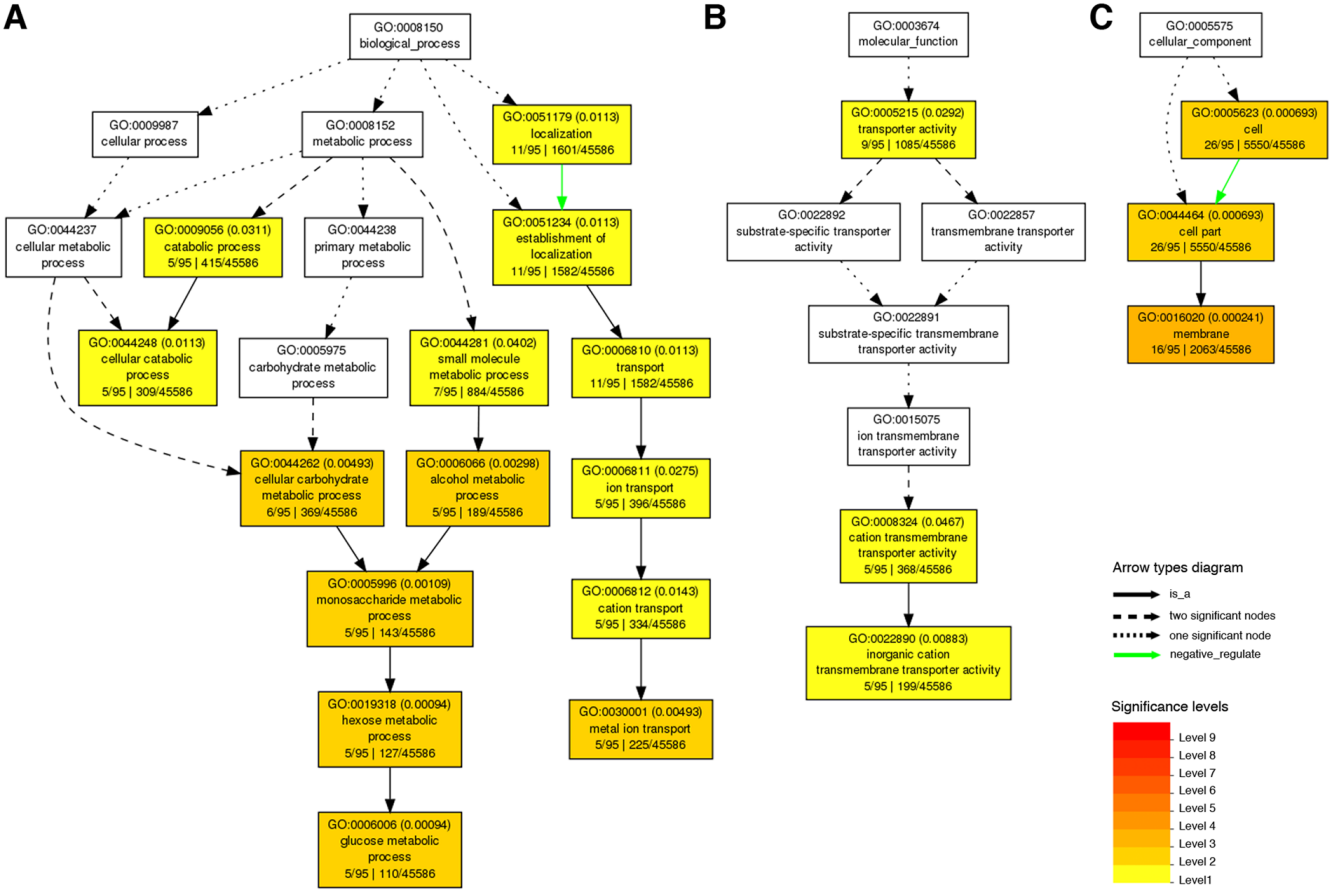
Gene ontology (GO) analysis of nitrogen starvation-repressed genes. AgriGO (http://bioinfo.cau.edu.cn/agriGO/) was used to analyze nitrogen starvation-repressed genes in 10-day-old rice seedling roots, and significantly enriched GO categories in biological process (**A**), molecular function (**B**), and cellular component (**C**) are shown in orange and yellow boxes (false discovery rate, FDR < 0.05).

In addition to −N-induced genes, we also performed GO and KEGG enrichment analyses on the 98 genes repressed by −N. In biological process, the GO terms such as “metal ion transport” and “glucose metabolic process” were significantly enriched (Fig. 7A). In molecular function, the GO terms “transporter activity” and “inorganic cation transmembrane transporter activity” were significantly enriched (Fig. 7B). In cellular component, the GO term “membrane” was significantly enriched (Fig. 7C). These results suggest that rapid changes in glucose metabolism to decrease the amounts of C skeleton for N assimilation and the reduction of various transporter activities are among the early events of N deprivation in rice roots. The information of −N-repressed genes enriched in GO analysis is provided in Supplementary Table S3.

KEGG pathway enrichment analysis of the 98 −N-repressed genes indicated that “pentose phosphate pathway (ko00030)”, “photosynthesis (ko00195)”, “nitrogen metabolism (ko00910)”, “carbon metabolism (ko01200)”, “fructose and mannose metabolism (ko00051)”, “carbon fixation in photosynthetic organisms (ko00710)”, “glutathione metabolism (ko00480)” and “biosynthesis of amino acids (ko01230)” were enriched. These results suggest that the expression of genes involved in N assimilation and production of C skeletons for amino acid biosynthesis is rapidly repressed by −N. The information of genes enriched in these pathways is provided in Supplementary Table S4. These GO and KEGG enrichment analyses highlight the importance of coordinated regulation of C and N metabolism in response to changes of N nutrients in rice seedlings.

### Analysis of metabolic and transporter genes rapidly repressed by −N

**Figure 8:**
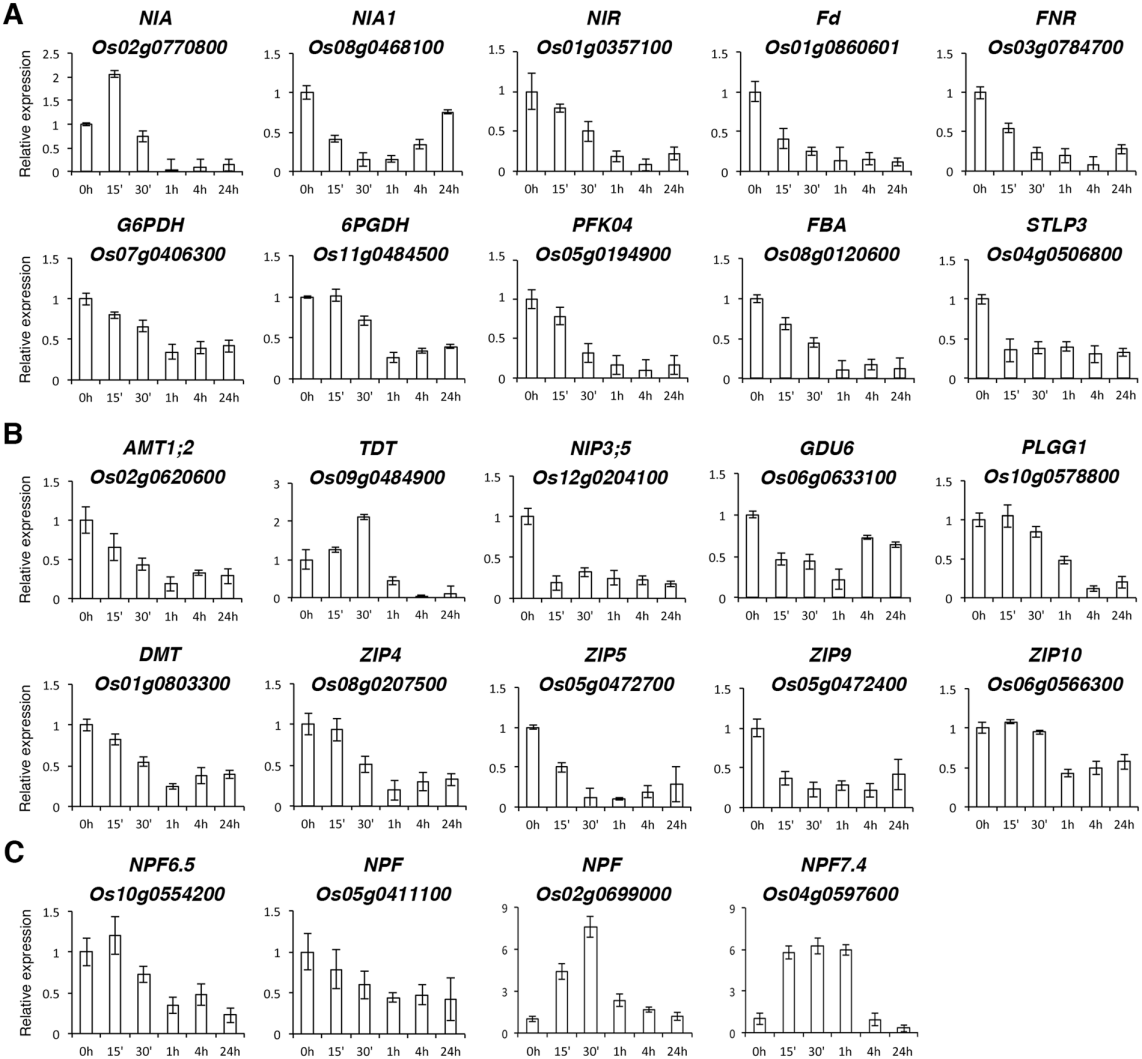
Quantitative RT-PCR analysis of genes repressed by nitrogen starvation (−N) in rice roots. (**A**) Carbon and nitrogen metabolic genes. (**B**) Transporter genes. (**C**) Nitrate transporter genes. RNA samples from roots of 10-day-old rice seedlings treated with −N for 0, 15 min, 30 min, 1 h, 4 h and 24 h were used for qRT-PCR analysis. The expression of nitrate transporter genes *Os10g0554200* (*NRT1.1B*/*NPF6.5*) and *Os05g0411100* (*NPF*) was rapidly repressed by −N, whereas the expression of *Os02g0699000* (*NPF*) and *Os04g0597600* (*NPF7.4*) was rapidly induced by −N. The expression level of each gene in the control sample (0 h) was set at 1. Relative expression represents the fold change of the target gene relative to that of the control. Data are mean ±SD of 3 biological replicates.

It is expected that −N will repress the expression of genes related to nitrate/nitrite assimilation, ferredoxin reduction, and the pentose phosphate pathway. Indeed, we found that the expression of *Os02g0770800* and *Os08g0468100* encoding nitrate reductase (NIA), *Os01g0357100* encoding nitrite reductase (NIR), *Os01g0860601* encoding ferridoxin (Fd), and *Os03g0784700* encoding ferredoxin-NADP reductase (FNR) was rapidly repressed by −N (Fig. 8A). The major function of glucose-6-phosphate dehydrogenase (G6PDH) and 6-phosphogluconate dehydrogenase (6PGDH) of the oxidative pentose phosphate pathway is to generate NADPH for the assimilation of inorganic N and fatty acid biosynthesis. The expression of *G6PDH* (*Os07g0406300*) and *6PGDH* (*Os11g0484500*) was also rapidly repressed by −N (Fig. 8A). In addition to *G6PDH* and *6PGDH*, genes involved in “cellular carbohydrate metabolic process (GO:0044262)” include *Os05g0194900* encoding ATP-dependent 6-phosphofructokinase 4 (PFK4), a key enzyme of the glycolysis pathway, *Os08g0120600* encoding fructose-bisphosphate aldolase (FBA), and *Os04g0506800* encoding sialyltransferase-like protein 3 (STLP3) (Supplementary Table S3). The expression patterns of these genes during the time course of −N treatment are shown in Fig. 8A.

In addition to genes involved in C and N metabolism, the expression of genes involved in the transport and allocation of C and N metabolites was also rapidly repressed by −N (Supplementary Table S3). For instance, the expression of *AMT1;2* (*Os02g0620600*) encoding a key ammonium transporter was rapidly and strongly repressed by −N (Fig. 8B). The expression of *Os09g0484900* encoding a tonoplast dicarboxylate transporter (TDT), *Os12g0204100* encoding an aquaporin nodulin 26-like intrinsic membrane protein (NIP3;5), *Os06g0633100* encoding glutamine dumper 6 (GDU6), *Os10g0578800* encoding plastidial glycolate/glycerate translocator 1 (PLGG1) and *Os01g0803300* encoding a drug/metabolite transporter (DMT) was also rapidly repressed by −N (Fig. 8B). Interestingly, we also found that the expression of four zinc transporter genes, *ZIP4* (*Os08g0207500*), *ZIP5* (*Os05g0472700*), *ZIP9* (*Os05g0472400*), and *ZIP10* (*Os06g0566300*), was rapidly repressed by −N in rice roots (Fig. 8B).

The rice nitrate transporter NRT1.1B/NPF6.5 has been demonstrated to play an important role in the regulation of N use efficiency^59^. Interestingly, the expression of *Os10g0554200* (*NRT1.1B*/*NPF6.5*) and *Os05g0411100* (*NPF*) was rapidly repressed by −N (Table 2 and Fig. 8C). By contrast, the expression of another two nitrate transporter genes, *Os02g0699000* (*NPF*) and *Os04g0597600* (*NPF7.4*), was rapidly induced by −N (Table 1 and Fig. 8C). It has been demonstrated that −N can induce the expression of high affinity nitrate transporter genes and repress the expression of low affinifity nitrate transporter genes^11,12^. It is possible that Os02g0699000 (NPF) and Os04g0597600 (NPF7.4) have higher affinifity to nitrate than Os10g0554200 (NRT1.1B/NPF6.5) and Os05g0411100 (NPF). Nevertheless, the physiological and biochemical features of these nitrate transporters have yet to be further characterized.

### Identification of genes that are sensitive to the availability of N in rice roots

**Figure 9:**
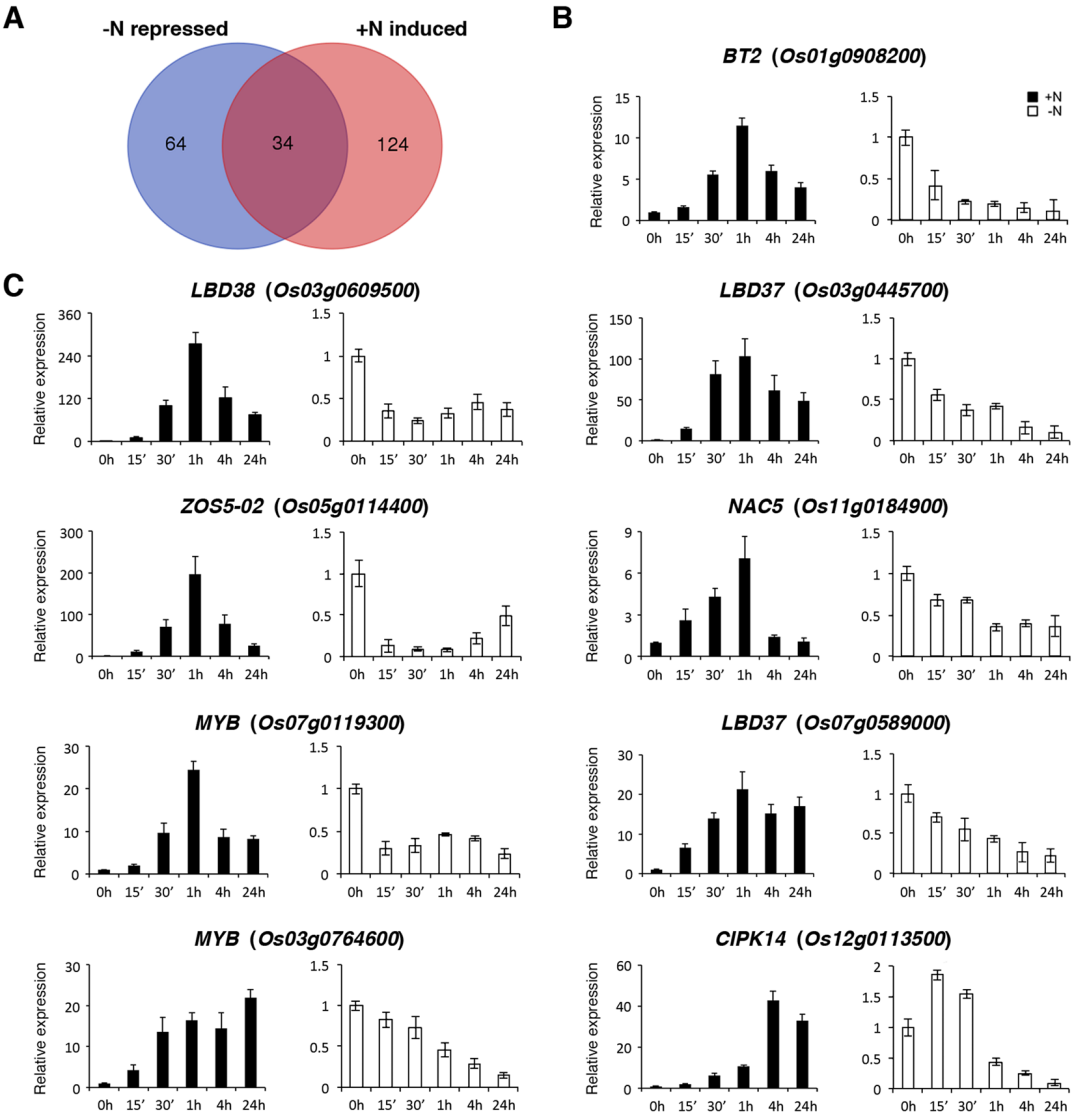
Identification of nitrogen-sensitive genes in rice seedling roots. (**A**) Venn diagram analysis of genes repressed by nitrogen starvation (−N) and induced by ammonium nitrate supplementation (+N)^34^. The expression of the 34 overlapped genes was rapidly induced by +N and quickly repressed by −N. RNA samples from roots of 10-day-old rice seedlings treated with +N or −N for 0, 15 min, 30 min, 1 h, 4 h and 24 h were used for qRT-PCR to analyze the expression of *BT2* encoding a nitrogen regulatory protein (**B**), and transcription factor/protein kinase genes (**C**). The expression level of each gene in the control sample (0 h) was set at 1. Relative expression represents the fold change of the target gene relative to that of the control. Data are mean ±SD of 3 biological replicates.

Venn diagram analysis of the 98 genes down-regulated by −N and the 158 genes up-regulated by +N identified 34 overlapped genes (Fig. 9A). The expression of these N-sensitive genes was rapidly induced by +N and quickly repressed by −N. A complete list of these 34 genes is shown in Table 4. As expected, genes related to nitrate/nitrite assimilation, ferredoxin reduction, and the pentose phosphate pathway are very sensitive to the availability of N in the growth medium (Table 4). The *BT2* (*Os01g0908200*) gene encoding a negative regulator of N use efficiency and several prominent candidate genes for the regulation of N response, including *LBD37* (*Os03g0445700*, *Os07g0589000*), and *LBD38* (*Os03g0609500*), are among the 34 genes identified here (Table 4). Still, we have identified several novel genes encoding potential N regulatory proteins, which may be involved in the regulation of N metabolism and/or signaling in rice roots.

**Table 4:** List of nitrogen-sensitive genes.

No.	Locus		Fold change		Gene description
			+N/−N	−N/+N	
1	Os02g0770800	LOC_Os02g53130	65.8	−16.7	Nitrate reductase [NAD(P)H]
2	Os01g0860601	LOC_Os01g64120	42.2	−5.6	Ferredoxin, root R-B1
3	Os01g0631200	LOC_Os01g44050	32.3	−7.0	Uroporphyrinogen-III C-methyltransferase
4	Os03g0609500	LOC_Os03g41330	30.5	−6.1	LOB domain-containing protein 38 (LBD38)
5	Os03g0684700	LOC_Os03g48030	25.8	−6.8	Integral membrane HPP family protein (HPP)
6	Os09g0484900	LOC_Os09g31130	15.7	−5.0	Tonoplast dicarboxylate transporter (TDT)
7	Os03g0445700	LOC_Os03g33090	15.0	−2.7	LOB domain-containing protein 37 (LBD37)
8	Os05g0114400	LOC_Os05g02390	14.0	−13.0	Zinc finger transcription factor, ZOS5-02
9	Os04g0640900	LOC_Os04g54830	13.4	−2.7	Unknown
10	Os11g0184900	LOC_Os11g08210	11.5	−7.4	NAC domain-containing protein 5 (NAC5)
11	Os07g0119300	LOC_Os07g02800	9.4	−2.1	MYB family transcription factor
12	Os07g0589000	LOC_Os07g40000	8.5	−4.8	LOB domain-containing protein 37 (LBD37)
13	Os03g0784700	LOC_Os03g57120	8.5	−2.8	Ferredoxin−NADP reductase (FNR)
14	Os03g0764600	LOC_Os03g55590	8.0	−3.1	MYB family transcription factor
15	Os01g0357100	LOC_Os01g25484	8.0	−2.4	Nitrite reductase
16	Os03g0243100	LOC_Os03g13950	6.5	−2.1	Actin-depolymerizing factor 5 (ADF5)
17	Os05g0360400	LOC_Os05g29710	5.9	−4.0	RING-type E3 ubiquitin-protein ligase EL5-like
18	Os12g0113500	LOC_Os11g02240	5.4	−3.5	CBL-interacting protein kinase 14 (CIPK14)
19	Os07g0406300	LOC_Os07g22350	5.1	−2.3	Glucose-6-phosphate dehydrogenase (G6PDH)
20	Os03g0823400	LOC_Os03g60840	5.0	−2.6	Bowman-Birk type trypsin inhibitor 13 (BBTI13)
21	Os06g0633100	LOC_Os06g42660	4.9	−2.6	Glutamine dumper 6 (GDU6)
22	Os05g0443700	LOC_Os05g37150	4.8	−3.0	Unknown, syntaxin 6 N-terminal domain protein
23	Os06g0566300	LOC_Os06g37010	4.5	−2.9	Zinc transporter 10 (ZIP10)
24	Os11g0484500	LOC_Os11g29400	4.3	−2.4	6-phosphogluconate dehydrogenase (6PGDH)
25	Os04g0475600	LOC_Os04g39980	3.1	−2.7	Dioxygenase for auxin oxidation (DAO)
26	Os05g0194900	LOC_Os05g10650	2.9	−6.2	ATP-dependent 6-phosphofructokinase 4 (PFK04)
27	Os01g0621900	LOC_Os01g43370	2.9	−2.3	Unknown, conserved peptide uORF-containing transcript
28	Os03g0838900	LOC_Os03g62240	2.9	−2.2	Unknown, mTERF domain-containing protein
29	Os05g0443500	LOC_Os05g37140	2.9	−2.4	Ferredoxin-6, chloroplastic
30	Os08g0207500	LOC_Os08g10630	2.6	−2.3	Zinc transporter 4 (ZIP4)
31	Os01g0908200	LOC_Os01g68020	2.4	−3.3	BTB/POZ and TAZ domain-containing protein 2 (BT2)
32	Os04g0649500	LOC_Os04g55600	2.3	−2.7	Unknown
33	Os04g0649600	LOC_Os04g55610	2.3	−2.7	Unknown
34	Os01g0747300	LOC_Os01g54340	2.2	−2.1	Unknown, PDDEXK nuclease-like family protein

Genes listed here are up-regulated by ammonium nitrate (+N/−N)^34^ and down-regulated by nitrogen starvation (−N/+N) for more than 2-fold after 30 min-1 h of treatments. Numbers of fold change are derived from the microarray data.

To verify the expression of these 34 N-sensitive genes, total RNA extracted from roots of 10-day-old rice seedlings treated with +N or −N for 0, 15 min, 1 h, 4 h, and 24 h was used for qRT-PCR analysis. The results of these +N and −N time course experiments confirmed that the expression of these 34 genes was rapidly induced by +N and quickly repressed by −N (Fig. 9B,C and Supplementary Fig. S5). For instance, the expression of the N regulatory gene *BT2* was rapidly regulated by +N and −N, but in the opposite way (Fig. 9B). The 34 N-sensitive genes include at least 7 transcription factor genes, *Os03g0609500* (*LBD38*), *Os03g0445700* (*LBD37*), *Os07g0589000* (*LBD37*), *Os05g0114400* (*ZOS5-02*), *Os11g0184900* (*NAC5*), *Os07g0119300* (*MYB*) and *Os03g0764600* (*MYB*), and one protein kinase gene, *Os12g0113500* (*CIPK14*). The expression patterns of these genes during the time course of +N and −N treatments are shown in Fig. 9C.

The expression patterns of genes related to nitrate/nitrite assimilation, ferredoxin reduction, and the pentose phosphate pathway during the +N and −N time course treatments are shown in Supplementary Fig. S5. The assimilation of N is highly dependent on the availability of C skeletons derived from glycolysis and the TCA cycle. In addition to N metabolic and regulatory genes, the expression of *Os05g0194900* encoding ATP-dependent 6-phosphofructokinase 4 (PFK04), a key enzyme of the glycolysis pathway, was rapidly induced by +N and quickly repressed by −N (Supplementary Fig. S5). The expression of 5 transporter genes, including *Os06g0633100* encoding glutamine dumper 6 (GDU6), *Os09g0484900* encoding tonoplast dicarboxylate transporter (TDT), *Os03g0684700* encoding an integral membrane HHP family protein (HHP), *Os08g0207500* encoding zinc transporter 4 (ZIP4) and *Os06g0566300* encoding zinc transporter 10 (ZIP10), was up-regulated by +N and down-regulated by −N (Supplementary Fig. S5).

The *Os04g0475600* gene encoding dioxygenase for auxin oxidation (DAO) is involved in catalyzing the irreversible oxidation of active indole-3-acetic acid (IAA) to biologically inactive 2-oxindole-3-acetic acid (oxIAA)^60^. Interestingly, the expression of *DAO* (*Os04g0475600*) was rapidly regulated by +N and −N treatments (Supplementary Fig. S5). Actin-depolymerizing factors (ADFs) are involved in the regulation of actin assembly, which affects cell growth, expansion, proliferation and differentiation. We have identified that one of the *ADF* genes, *ADF5* (*Os03g0243100*), is a N-sensitive gene (Supplementary Fig. S5). This implicates that the N status may rapidly and directly regulate cell growth and differentiation via the reorganization of cytoskeletons in rice roots. In addition to BT2, the ubiquitin-mediated proteolytic degradation machinery has been shown to modulate N responses in Arabidopsis^61^. Interestingly, the expression of *Os05g0360400* encoding RING-type E3 ubiquitin-protein ligase EL5-like was rapidly regulated by the availability of N in the growth medium (Supplementary Fig. S5).

In addition, the expression of *Os03g0823400* encoding a Bowman-Birk type trypsin inhibitor (BBTI) was rapidly induced by +N and quickly repressed by −N (Supplementary Fig. S5). We previously found that the expression of *BBTI* was also rapidly induced by glutamine and glutamate^32,33^. Trypsin inhibitor is usually associated with defense response^62^. It is not clear why the expression of this particular *BBTI* (*Os03g0823400*) gene is tightly regulated by the availability of N in the growth medium. The *Os05g0443700* gene, one of the 7 unknown function genes identified here, encodes a syntaxin 6 N-terminal domain-containing protein, which is commonly found in various SNARE proteins involved in endosomal transport^63^. The rapid response of *Os05g0443700* to +N and −N treatments (Supplementary Fig. S5) suggest that the encoded protein may be involved in cell trafficking associated with N metabolism and/or signaling. The expression patterns of the other 6 unknown function genes (*Os04g0640900, Os01g0621900, Os03g0838900, Os04g0649500, Os04g0649600, Os01g0747300*) during the time course of +N and −N treatments are shown in Supplementary Fig. S5.

### Analysis of −N-repressed transcription factor genes

**Figure 10:**
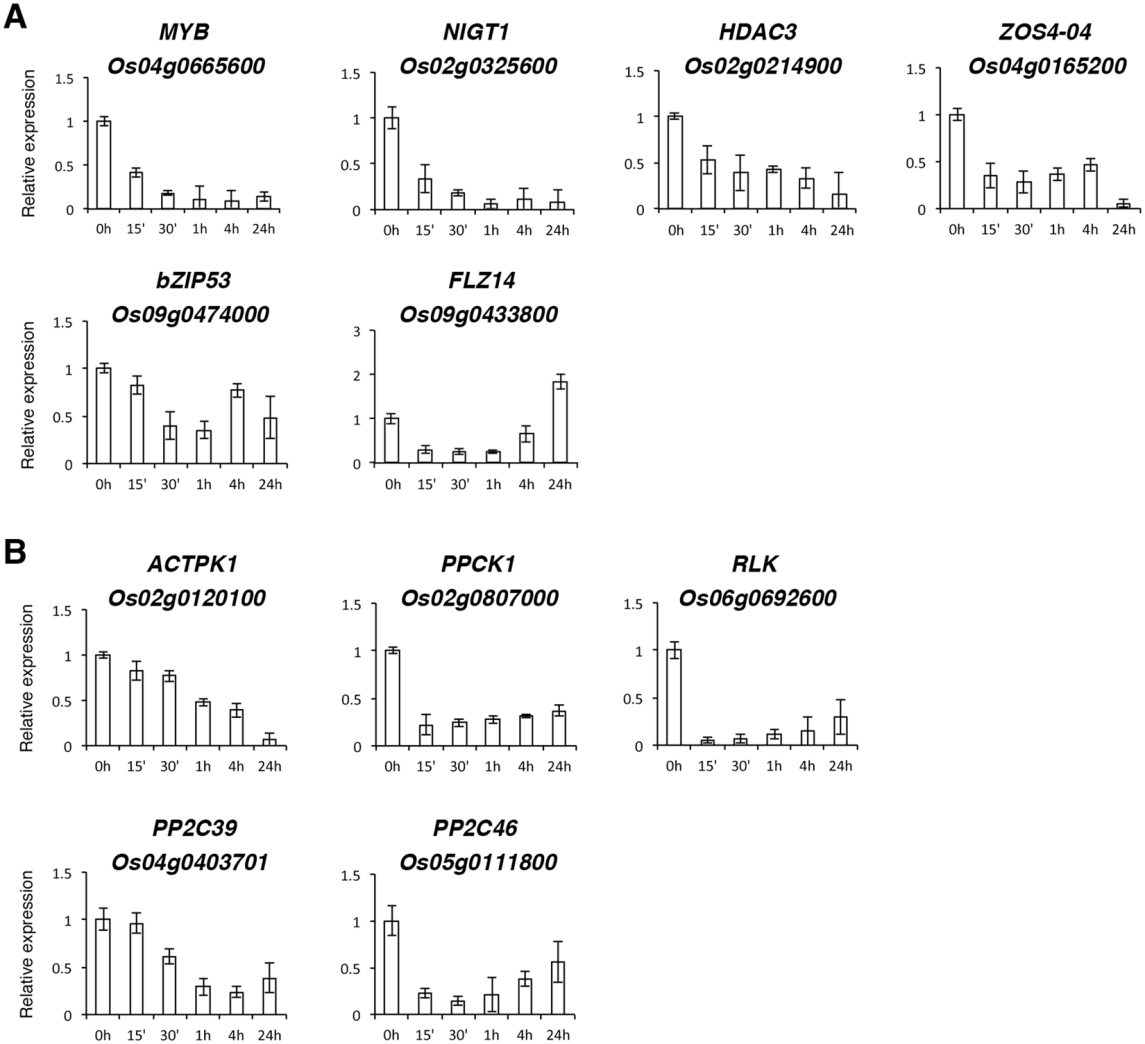
Expression of transcription factor and protein kinase/phosphatase genes rapidly repressed by nitrogen starvation in rice roots. RNA samples from roots of 10-day-old rice seedlings treated with nitrogen starvation for 0, 15 min, 30 min, 1 h, 4 h and 24 h were used for qRT-PCR to analyze the expression of genes encoding transcription factor/nuclear protein (**A**), and protein kinase/phosphatase (**B**). The expression level of each gene in the control sample (0 h) was set at 1. Relative expression represents the fold change of the target gene relative to that of the control. Data are mean ±SD of 3 biological replicates.

In addition to the 7 N-sensitive transcription factor genes shown in Fig. 9C, we have identified at least 6 more genes encoding transcription factors or nuclear proteins, including *Os04g0665600* (*MYB*), *Os02g0325600* (*NIGT1*), *Os02g0214900* (*HDAC3*, *HISTONE DEACETYLASE 3*), *Os04g0165200* (*ZOS4-04*), *Os09g0474000* (*bZIP53*) and *Os09g0433800* (*FLZ14*), that were rapidly repressed by −N. NIGT1 is a N regulatory protein^29,30^. The enzyme histone deacetylase 3 (HDAC3) may have a global effect on gene expression via chromosome modification. The functions of *Os04g0665600* encoding a MYB family protein, *Os04g0165200* encoding a zinc-finger protein (ZOS4-04), *Os09g0474000* encoding basic leucine zipper 53 (bZIP53) and *Os09g0433800* encoding FCS-like zinc finger protein 14 (FLZ14) are unknown. The expression patterns of these 6 genes during the time course of −N treatment are shown in Fig. 10A. The expression of *Os04g0665600* (*MYB*) and *Os02g0325600* (*NIGT1*) was strongly and continuously repressed by −N (Fig. 10A). By contrast, the expression of *Os09g0474000* (*bZIP53*) and *Os09g0433800* (*FLZ14*) was only transiently repressed within 1 h of −N treatment (Fig. 10A).

### Analysis of −N-repressed protein kinase/phosphatase genes

We identified at least 4 protein kinase and 2 phosphatase genes that were rapidly repressed by −N in rice seedling roots (Table 2). *CIPK14* (*Os12g0113500*) is a N-sensitive gene that is rapidly regulated by +N and −N treatments as shown in Fig. 8B. The expression patterns of the other protein kinase/phosphatase genes, e.g. *Os02g0120100* (*ACTPK1*), *Os02g0807000* (*PPCK1*), *Os06g0692600* (*RLK*), *Os04g0403701* (*PP2C39*), and *Os05g0111800* (*PP2C46*), during the time course of −N treatment are shown in Fig. 10B. ACTPK1 (Os02g0120100) has been demonstrated to phosphorylate and inactivate the ammonium transporter AMT1;2 in rice seedling roots under sufficient ammonium conditions^31^. The *Os02g0807000* gene encodes phosphoenolpyruvate carboxylase kinase 1 (PPCK1) that plays an important role in the regulation of phosphoenolpyruvate carboxylase (PEPC) and plant metabolism. The functions of the other protein kinases/phosphatases and/or their roles in the regulation of N response have yet to be characterized in rice.

### Genes rapidly regulated by N starvation, glutamine, and glutamate

In addition to ammonium nitrate, we previously used transcriptome analysis followed by RT-PCR or qRT-PCR verification to identify genes that were rapidly regulated by glutamine (+Gln, 2.5 mM, 30 min) or glutamate (+Glu, 2.5 mM, 30 min) in rice roots^32,33^. In the +Gln study, we only identified 35 up-regulated genes, whereas 122 up- and 4 down-regulated genes were identified in the +Glu study^32,33^. None of the 35 + Gln-induced genes were induced by −N (Supplementary Fig. S6A). By contrast, 10 of the 35 Gln-induced genes were rapidly repressed by −N (Supplementary Fig. S6B and Supplementary Table S5). Interestingly, 5 of the 10 + Gln-induced and −N-repressed genes encode transcription factors, e.g. *ZOS5-02* (*Os05g0114400*), *NAC5* (*Os11g0184900*), *LBD37* (*Os07g0589000*), *LBD37* (*Os03g0445700*), and *MYB* (*Os07g0119300*) (Supplementary Table S5). The other 5 + Gln-induced and −N-repressed genes are *CIPK14* (*Os12g0113500*), *GDU6* (*Os06g0633100*), *PFK04* (*Os05g0194900*), *BBTI13* (*Os03g0823400*), and *Os09g0482800* encoding an EF-hand domain-containing protein (Supplementary Table S5).

Interestingly, the expression of 9 +Gln-induced and −N-repressed genes, except *Os09g0482800* encoding an EF-hand domain-containing protein, was also rapidly induced by +N (Table 4)^34^. These results suggest that part of the inorganic N signaling pathways may be mediated via glutamine, and some of the transcription factors/regulatory proteins identified here may be involved in these processes in rice seedlings.

Venn diagram analysis of genes induced by −N and +Glu identified 12 overlapped genes (Supplementary Fig. S7A and Table S6.). The −N- and +Glu-induced genes include *Os09g0455300* (*bHLH120*), *Os12g0478400* (*WAK125*), *Os08g0508800* (*LOX2*), *Os02g0627100* (*PAL4*), *Os01g0882800* encoding amino acid permease 8, *Os12g0518200* encoding a drug/metabolite transporter (DMT), *Os08g0473900* (α-amylase isozyme 3D), *Os01g0666000* encoding lipid phosphate phosphatase 2, *Os03g0318400* encoding aspartic proteinase nepenthesin-1, *Os01g0705200*, encoding a late embryogenesis abundant protein, *Os03g0194600* encoding cytochrome b561 and DOMON domain-containing protein, and *Os06g0292400* encoding an unknown function protein. Exogenous Glu has been implicated to induce defense response^33,64^. It is possible that +Glu and −N may share some components related to stress and/or defense response. For instance, the *Os08g0508800* gene encodes a LOX2 homolog that may play an important role in the biosynthesis of JA in rice. The expression of *Os08g0508800* (LOX2) was commonly induced by +Glu^33^ and −N (Fig. 4B). It will be interesting to further investigate if JA, as well as the other +Glu- and −N-induced genes, are involved in the interaction between +Glu and −N signaling pathways.

By contrast, comparison between the −N-repressed and +Glu-induced genes revealed that the expression of *NAC5* (*Os11g0184900*), *LBD37* (*Os07g0589000*), *MYB* (*Os07g0119300*), *BBTI13* (*Os03g0823400*), *TDT* (*Os09g0484900*), and *Os09g0482800* encoding an EF-hand domain-containing protein was rapidly regulated by +Glu and −N (Supplementary Fig. S7B and Table S7). Interestingly, 4 of the 6 + Glu-induced and −N-repressed genes, e.g. *NAC5* (*Os11g0184900*), *LBD37* (*Os07g0589000*), *MYB* (*Os07g0119300*) and *BBTI* (*Os03g0823400*), were also commonly induced by +N and +Gln^32–34^. The *Os09g0482800* gene encoding an EF-hand domain-containing protein is only shared by +Gln and +Glu, whereas the *TDT* (*Os09g0484900*) gene is commonly induced by +N and +Glu^32–34^. Nevertheless, these analyses futher support the notion that the transcription factors NAC5 (Os11g0184900), LBD37 (Os07g0589000) and MYB (Os07g0119300) are potential N regulatory proteins in rice. None of the 4 +Glu-repressed genes overlapped with the genes up- or down-regulated by −N.

## Discussion

Transcriptomic analysis using microarray or RNA-Seq has been applied to identify genes that are differentially regulated by −N in rice^25–27^. However, these studies were either performed with a relatively long time of N deprivation or the identified genes were not verified by independent experiments. Here, we used a more stringent approach to identify genes that were rapidly regulated by −N in rice roots. All genes identified by microarray analysis were further verified by qRT-PCR, and only those genes that had fold-change greater than 2 (−N/+N) in both experiments were used for GO and KEGG enrichment analyses. Approximately 19% of the up-regulated and 32% of the down-regulated genes identified by microarray analysis did not pass the verification by qRT-PCR. In addition to genes involved in C and N metabolism, the expression of genes related to “plant hormone signal transduction” and “transporter activity” is rapidly regulated by −N in rice roots.

The expression of genes related to nitrate/ammonium uptake, nitrate/nitrite assimilation, ferredoxin reduction, the pentose phosphate pathway, and glucose metabolic process was rapidly repressed by −N (Table 2, Fig. 7 and Supplementary Fig. S4). By contrast, the expression of genes involved in the release of ammonium, including *PAL3* (*Os02g0626600*), *PAL4* (*Os02g0627100*), and *MGL* (*Os10g0517500*) was rapidly induced by −N (Figs 3 and 4 and Table 1). These results suggest that the recycling of ammonium from amino acids is one of the early events during −N in rice seedlings. The release of ammonium from amino acids may provide the initial demand of N during the sudden change from N sufficient to N deficient conditions.

In bacteria, guanosine pentaphosphate and tetraphosphate (p)ppGpp play a major role in the stringent response such as nutrient starvation^65^. Bacterial (p)ppGpp is synthesized from ATP and GTP/GDP by the RelA and SpoT enzymes, which modulates target enzymes to reduce cell proliferation to conserve resources and activates the acclimatory pathways^65^. Genes encoding RelA and SpoT homologs (RSH) are widespread in plants and algae, which may play an important role in influencing plant growth and stress acclimation^66^. Interestingly, the expression of *Os05g0161500* encoding chloroplastic GTP diphosphokinase/calcium-activated RelA-SpoT homolog 2 (CRSH2) was rapidly induced by −N in rice roots (Table 1, Fig. 4A). CRSH2 contains a central RelA-SpoT domain and two EF-hand motifs for calcium binding that may function as a Ca^2+^-activated (p)ppGpp synthetase to integrate the Ca^2+^ and (p)ppGpp signaling pathways^38^. It will be interesting to investigate if CRSH2 and its product (p)ppGpp are involvled in the acclimatory responses during N deficiency in rice.

The metabolism of C and N is highly interdependent as the assimilation of inorganic N requires C skeletons derived from glycolysis and the TCA cycle. Thus, the production and flux of C skeletons has to be regulated to match the demands under various N conditions. However, how the N status is perceived to regulate C metabolism and flux is unknown. Phosphofructokinase (PFK) catalyzes a key regulatory step of the glycolysis pathway. The expression of *PFK04* (*Os05g0194900*), one of the 15 *PFK* genes identified in rice^67^, was rapidly induced by +N and quickly repressed by −N in rice roots (Table 4, Supplementary Fig. S5). Dicarboxylate transporters play an important role in the transport and compartmentation of C metabolites^68^. The expression of *TDT* (*Os09g0484900*) was co-regulated with *PFK04* and N assimilatory genes in response to the availability of N in the environment (Table 4, Supplementary Fig. S5). It is possible that PFK04 is one of the key enzymes that coordinately regulate C metabolism, and the intracellular dicarboxylate transporter TDT may modulate the levels of dicarboxylate in different cellular compartments in response to the demand of N assimilation in rice roots.

The inter-dependency of C and N metabolism suggests that the signal transduction pathways underlying C and N deficiency may also interact with each other. The regulatory proteins CIPK14/CIPK15 have been shown to coordinate the responses to oxygen and sugar deficiency in rice^69^. Interestingly, *CIPK14*/*CIPK15* are N-sensitive genes as the expression of *CIPK14*/*CIPK15* in rice roots was rapidly regulated by the availability of N in the growth medium (Fig. 9). This finding further supports the notion that CIPK14/15 may coordinate the C and N signaling pathways in response to the relative C/N status in rice^69^.

In addition to rapid changes in C and N metabolism, the homeostasis of plant homones such as IAA, JA, and ABA, and their signal transduction pathways may be associated with the early events of N deficiency in rice. It is known that auxin/IAA is involved in the regulation of root system archetiture in response to nitrate and N deficiency in Arabidopsis^70,71^. By contrast, how auxin/IAA regulates the growth and development of rice roots in response to N deficiency is largely unknown. DAO (Os04g0475600) catalyzes the irreversible oxidation of IAA to oxIAA in rice^60^. The discovery that *DAO* is a N-sensitive gene provides insights into the involvement of IAA oxidation in the modulation of N responses in rice roots. In addition to IAA oxidation, the formation of IAA-glucose conjugate or IAA methyl ester (MeIAA) is one of the molecular modifications controlling IAA homeostasis and activity. Interestingly, the expression of *Os01g0179600* encoding indole-3-acetate beta-D-glucosyltransferase (IAGLU) and *Os06g0323100* encoding indole-3-acetate O-methyltransferase 1 (IAMT1) was rapidly repressed by −N (Table 2, Supplementary Fig. S4). These results suggest that N deficiency may increase the amount of IAA via decreasing the formation of oxidized- and conjugated-IAA, which in turn activates the IAA signaling pathway. In accordance with this hypothesis, the expression of *PIN9* (*Os01g0802700*), *SAUR19* (*Os06g0702000*), *SAUR36* (*Os04g0608300*) and *Os02g0143400* encoding auxin-induced protein X15 was rapidly induced by −N (Table 1 and Supplementary Fig. S3). Thus, the oxidation and modifications of IAA may play a role in mediating N-deficient responses in rice roots.

In addition to IAA, plant hormones JA and ABA may be also involved in the regulation of −N responses in rice roots. The *TIFY11a* (*JAZ9*, *Os03g0180800*), *TIFY11c* (*JAZ11*, *Os03g0180900*), and *TIFY11e* (*JAZ13*, *Os10g0391400*) genes identified in the categories of “plant hormone signal transduction” and “plant-pathogen interaction” encode key components of the JA signaling pathway^49^. The *Os08g0508800* gene identified in the “linoleic acid metabolism” encodes a LOX2 homolog, which is a key enzyme of the JA biosynthesis pathway^72^. The expression of these genes and *Os04g0308500* encoding a 23 kDa jasmonate-induced protein was rapidly induced by −N (Table 1, Figs 4B and 5A and Supplementary Fig. S3). These results indicate that the JA signal transduction pathways are among the early responses associated with N deficiency in rice roots.

The plant hormone ABA is derived from the carotenoid biosynthesis pathway. The −N-induced genes *PSY3* (*Os09g0555500*) and *BCH1* (*Os03g0125100*) are associated with ABA biosynthesis^39–42^. The rice *PSY* gene family consists of 3 members. *PSY1* and *PSY2* are involved in light-regulated carotenoid biosynthesis, whereas *PSY3* is devoted to abiotic stress-induced ABA formation^39,40^. The *Os03g0125100* gene encoding β-carotene hydroxylase 1 (BCH1) was shown to confer drought and oxidative stress resistance by increasing xanthophylls and ABA in rice^41,42^. Moreover, the expression of *PSY3* and *BCH1* is induced by ABA^39,41^. Interestingly, we found that −N could rapidly and strongly induce the expression of *PSY3* and *BCH1* (Fig. 4B). The expression of ABA-responsive transcription factor genes *HOX22* and *bHLH120* was also rapidly and strongly induced by −N (Fig. 5A). These results suggest that ABA biosynthesis and signaling are among the early events induced by N deficiency in rice roots. Recently, ABA was shown to regulate auxin homeostais to promote root hair elongation in rice root tips^73^. It is known that N deficiency will induce cell division as well as cell elongation to promote primary root growth in rice^37^. It is possible that these processes are governed by auxin and the interactions between auxin and ABA in rice.

ROS production was shown to be associated with K, P, and N deficiencies in Arabidopsis^74^. The rapid induction of genes encoding peroxidase or peroxidase-like proteins (*Os06g0521500*, *Os05g0135400*, and *Os06g0522300*) and *Os07g0468100* encoding GSTU1 (Fig. 4C) indicates that N deficiency may also cause ROS production in rice roots. In addition, the expression of several oxidative stress-responsive genes was also rapidly induced by −N (Table 1). For instance, the expression of *GolS1* (*Os03g0316200*) and *GolS2* (*Os07g0687900*) encoding galactinol synthase, a key enzyme for the synthesis of raffinose family oligosaccharide to protect plants from oxidative damage^44^, was rapidly and strongly induced by −N (Table 1 and Fig. 4C). These results implicate that the production of ROS and redox signaling pathways are among the early events associated with N deficiency in rice roots.

We have identified several transcription factor genes, including *NIGT1*, whose expression is rapidly regulated by −N (Figs 5, 9 and 10). The functions of these genes in the regulation of N responses are mostly uncharacterized in rice. The Arabidopsis LBD/37/38/39 transcription factors have been demonstrated to regulate N responses^28^. Interestingly, the expression of *Os07g058900*, *Os03g0445700* and *Os03g0609500* encoding LBD37/38 homologs is co-regulated with *NIA* and *NIR* in response to changes of N availability (Fig. 9). It is likely that the LBD37/38 homologs also play a key role in the regulation of N responses in rice. The NAC5 transcription factor is involved in stress tolerance^75–77^, but its role as a N regulatory protein has yet to be characterized. Further studies on NAC5 may provide insights into the interaction between the N response and stress signaling pathways in rice. In addition to transcription factor genes, we have also identified several protein kinase/phosphatase genes that are rapidly up- or down-regulated by −N (Figs 5, 9 and 10). The functions of these genes, except *ACTPK1*, in the regulation of N responses are unknown. Further studies on these potential N regulatory genes may provide a new solution to enhance N use efficiency in rice.

Components of the ubiquitin-mediated proteolytic degradation machinery have been shown to modulate N responses in Arabidopsis^61^. The Arabidopsis BTB protein acts as a substrate-specific adapter of an E3 ubiquitin-protein ligase complex (CUL3-RBX1-BTB), which mediates the ubiquitination and subsequent proteasomal degradation of target proteins^78,79^. One of the Arabidopsis BTB proteins, BT2, has been shown to mediate multiple responses to nutrients, stresses, and hormones^35,80,81^. The rice BT2 homolog (Os01g0908200) functions as a negative regulator of nitrate transporter genes and N use efficiency^35^. It is possible that the ubiquitin-mediated proteolytic degradation machinery using BT2 as a hub may also interconnect N, hormone, and stress signaling pathways in rice. Interestingly, *BT2* and *Os05g0360400* encoding RING-type E3 ubiquitin-protein ligase EL5-like are N-sensitive genes. The expression of *BT2* and *EL5-like* was co-regulated with *NIA* and *NIR* in response to changes of N in the growth medium (Table 4, Fig. 9B and Supplementary Fig. S5). Ubiquitin ligase EL5 has been shown to maintain the viability of root meristems by influencing cytokinin-mediated nitrogen effects in rice^82^. The function of EL5-like (Os05g0360400) protein has yet to be characterized. It is worthy to further investigate if BT2 and EL5-like (Os05g0360400) are involved in the regulation of N response and the crosstalk among multiple signaling pathways in rice.

GO enrichment analysis revealed that transporter genes were enriched in −N-repressed genes (Fig. 7 and Table 2). Interestingly, some of the −N-repressed transporter genes were also rapidly induced by +N (Table 4). In addition to *TDT*, the expression of *GDU6*, *ZIP4*, *ZIP10* and *Os03g0684700* encoding an integral membrane HPP family protein was co-regulated with *NIA* and *NIR* in response to the availability of N (Supplementary Fig. S5). Glutamine dumpers are plant-specific membrane proteins that are involved in nonselective amino acid export^83,84^. GDU6 may modulate the transport of amino acids in response to changes of N in the growth medium. Members of integral membrane HPP family are predicted to contain 4 transmembrane domains and a conserved HPP motif (Pfam: PF04982). Some of the Arabidopsis HPP family proteins are nitrate-inducible components of the nitrite transport system of plastids^85^. It will be interesting to further study if HPP (Os03g0684700) is a nitrite transporter in rice. Zinc is an essential element that functions as a catalytic or structural co-factor in a large number of enzymes and regulatory proteins in plants^86^. It has been shown that improved N nutrition can enhance zinc uptake and remobilization in plants^87^. However, it is not clear if the uptake and remobilization of zinc will affect N metabolism. The discovery that *ZIP4* and *ZIP10* are N-sensitive genes raises an interesting question whether the homeostasis of zinc plays a role in the regulation of N response. Still, we cannot exclude the possibility that some of the zinc transporters may transport zinc as well as the other compounds associated with N metabolism.

We previously used microarray analysis followed by RT-PCR or qRT-PCR verification to identify genes that were rapidly regulated by +N, +Gln, and +Glu in rice roots^32–34^. Comparison of these results revealed that there were at least 7 genes that were commonly induced by +N, +Gln, and +Glu^34^. We proposed that these genes may be involved in the regulation of general N responses in rice roots regardless the forms of N source^34^. Interestingly, 4 of these 7 genes, e.g. *LBD37* (*Os07g058900*), *NAC5* (*Os11g0184900*), *MYB* (*Os07g0119300*), and *BBTI13* (*Os03g0823400*), are among the N-sensitive genes identified here (Fig. 9C and Supplementary Fig. S5). Previously, Gln was found to be rapidly accumulated in the roots of rice seedlings after 15–30 min of +N treatment^34^. Similarly, Gln also rapidly accumulated after feeding of Glu in rice seedling roots^33^. Here, we found that levels of Gln were rapidly reduced within 15 min of −N treatment in rice roots (Fig. 2). These results suggest that the endogenous levels of glutamine are very sensitive to the availability of N in the environment. It is conceiveable that part of the general N signal may be mediated by Gln. Nonetheless, further studies on the putative N regulatory genes identified here may provide insights into the regulation of N signaling pathways in rice roots.

## Methods

### Plant material and growth conditions

The rice plant *Oryza sativa* L. ssp. *japonica* cv. TNG67 was used in all experiments. Rice seeds were surface-sterilized and placed in darkness at 30 °C for 3 days. The germinated seedlings were transferred to 5-inch square pots filled with hydroponic solutions and placed in a growth chamber at 30 °C for 7 days under a 12 h light/12 h dark cycle, 200 µmol photons m^−2^ s^−1^ light intensity, and 70% relative humidity. The hydroponic solution recommended by the International Rice Research Institute containing 1.43 mM NH_4_NO_3_ was used as +N treatment^36^, and the same hydroponic solution without the addition of 1.43 mM NH_4_NO_3_ was used as −N treatment in all experiments. For −N treatment, 10-day-old rice seedlings grown in +N hydroponic solution were transferred to −N hydroponic solution for 1 h or the indicated time. The nutrient solution was completely renewed every 3 days.

### Leaf chlorophyll measurement

Leaf chlorophyll measurement was conducted with the Chlorophyll Content Meter (CCM-300, Opti-sciences, NH, USA) as described previously^34^. Fifteen leaves from 15 rice seedlings grown in +N or −N hydroponic solution were used for the measurement.

### Microarray analysis

The GeneChip Rice Genome Array (Affymetrix, Santa Clara, CA, USA) was used for transcriptome analysis. The extraction of total RNA from roots of 10-day-old rice seedlings grown in +N or −N (1 h) was conducted as described previously^88^. RNA samples of three biological replicates from +N- or −N-treated rice seedling roots were submitted to the Affymetrix Gene Expression Service Lab at Academia Sinica (http://ipmb.sinica.edu.tw/affy/) for microarray analysis. The experimental procesdures and criteria for selecting −N-regulated genes were performed as described previously^32^. AgriGO (http://bioinfo.cau.edu.cn/agriGO/) and EXPath (http://expath.itps.ncku.edu.tw) were used for GO and KEGG enrichment analysis of the −N-regulated genes, respectively. The nomenclature of genes listed in Tables 1 and 2 is according to the annotation in NCBI (https://www.ncbi.nlm.nih.gov/gene/) or relevant literatures.

### Quantitative RT-PCR analysis of genes responsive to −N or +N treatment

Total RNA extracted from 10-day-old rice seedlings treated with −N or +N for the indicated time was used for qRT-PCR analysis with 3 biological replicates. The expression of nuclear genes *UBC3* (*Os02g0634800*) and *UBQ10* (*Os02g 0161900*) was used to normalize the qRT-PCR data. The primer sequences used in this study are listed in Supplementary Tables S8 and S9. The sequences of *CIPK14* (*Os12g0113500*) and *CIPK15* (*Os11g0113700*) are highly identical. The expression of these two genes cannot be distinguished by qRT-PCR with the primers used in this study.

### Analysis of free amino acids in rice roots

The Waters Acquity UPLC system was used to analyze free amino acids extracted from 10-day-old rice seedlings treated with −N for 0–4 h. Amino acid extraction and analysis were performed as described previously^32^.

## Electronic supplementary material

Supplementary Information

## Data Availability

The microarray datasets generated in this study are available in the NCBI GEO repository GSE109649 (https://www.ncbi.nlm.nih.gov/geo/query/acc.cgi?acc=GSE109649). All other data generated or analyzed during this study are included in this published article and its Supplementary Information files.
