# Supplementary material for: Early molecular events associated with nitrogen deficiency in rice seedling roots
Source: Sci Rep. 2018 Aug 15;8:12207. doi: 10.1038/s41598-018-30632-1 (PMC6093901; doi:10.1038/s41598-018-30632-1)
Supplement: Supplementary file 1 — Supplementary Information [file 41598_2018_30632_MOESM1_ESM.pdf]

Early molecular events associated with nitrogen deficiency in rice seedling roots

Ping-Han Hsieh†, Chia-Cheng Kan†, Hsin-Yu Wu, Hsiu-Chun Yang, and Ming-Hsiun Hsieh*

Supplementary Information

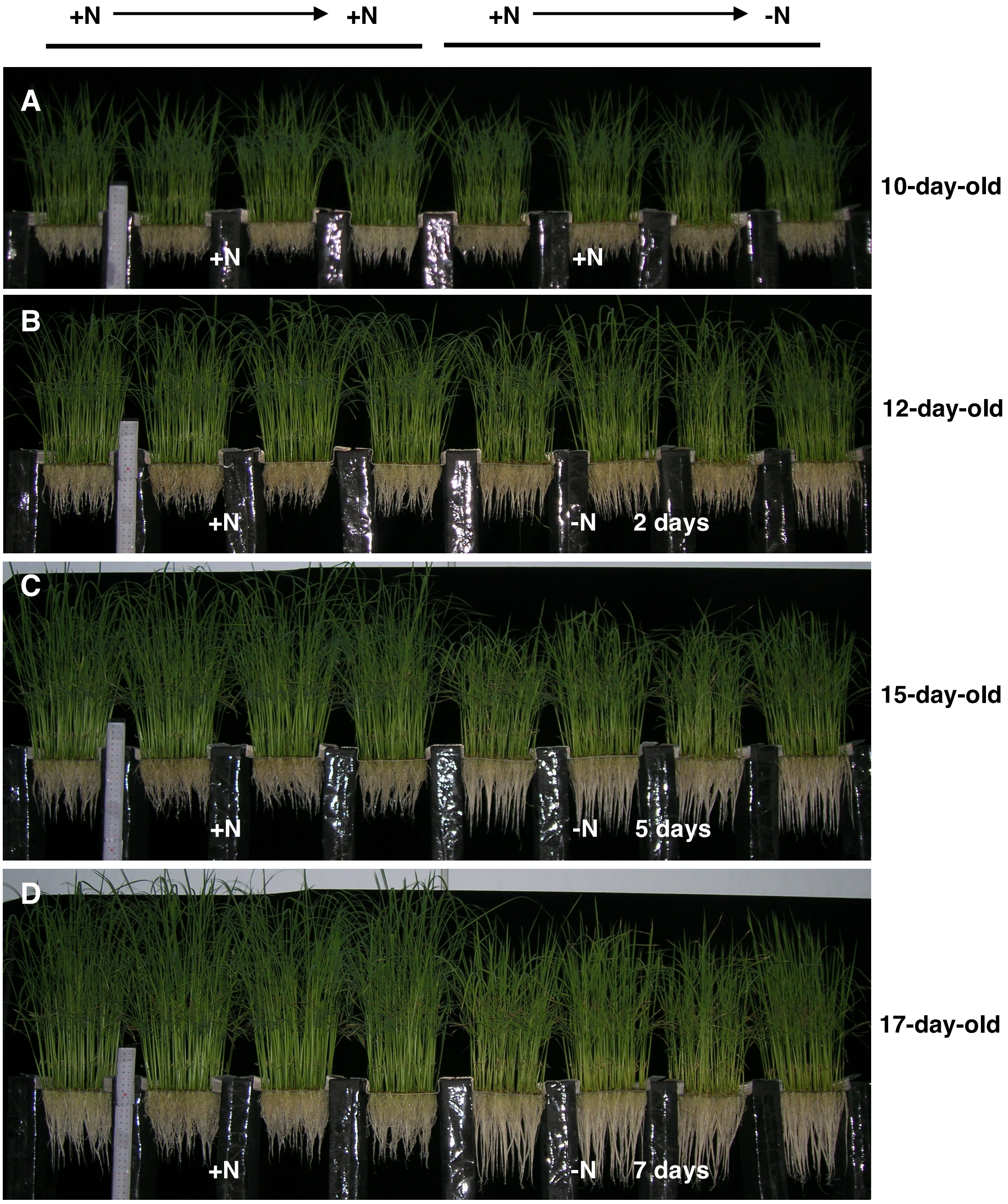

Fig. S1 Adaptation of rice seedlings to nitrogen deficiency (-N). **(A)** Ten-day-old rice seedlings grown in hydroponic solutions containing 1.43 mM ammonium nitrate (+N). **(B-D)** Plants in **(A)** were transferred to hydroponic solutions with or without N for 2, 5, and 7 days. -N inhibits shoot growth and promotes root growth in rice seedlings.

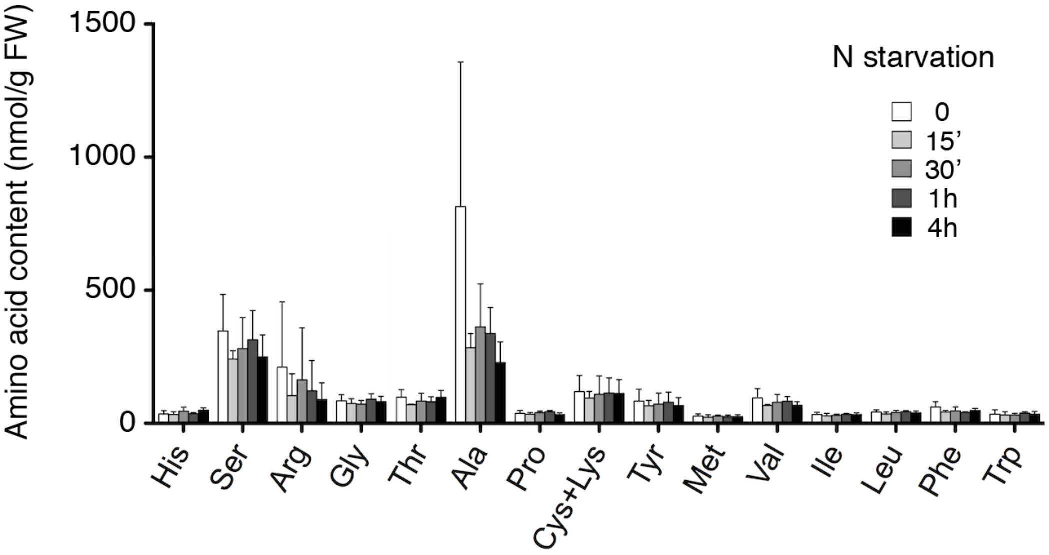

Fig. S2 Amino acid contents in the roots of rice seedlings during the time course of nitrogen starvation treatment. The amounts of amino acids shown here do not have significant differences. Data are means ± SD (*n* = 3).

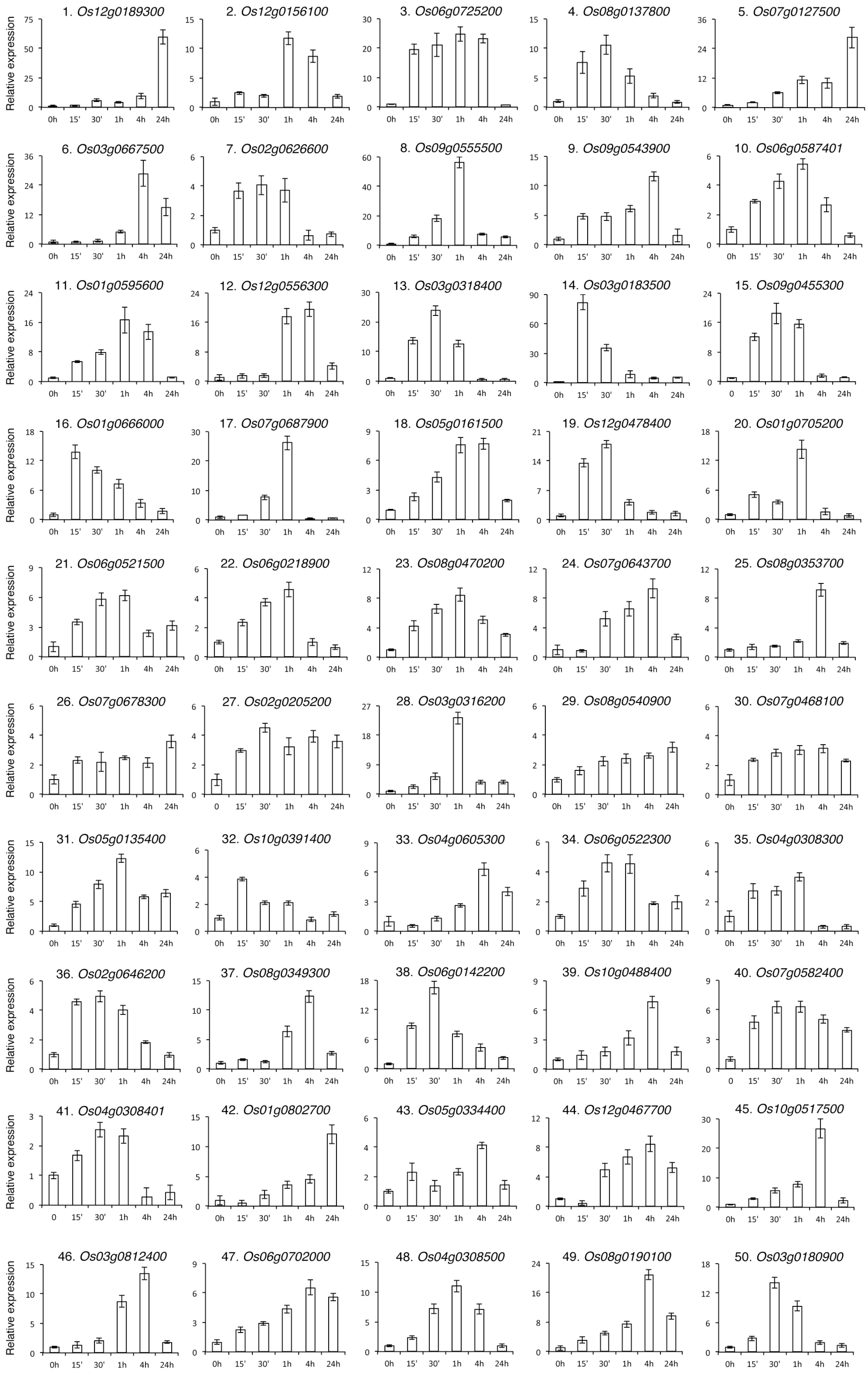
Relative expression
1. Os12g0189300
2. Os12g0156100
3. Os06g0725200
4. Os08g0137800
5. Os07g0127500
6. Os03g0667500
7. Os02g0626600
8. Os09g0555500
9. Os09g0543900
10. Os06g0587401
11. Os01g0595600
12. Os12g0556300
13. Os03g0318400
14. Os03g0183500
15. Os09g0455300
16. Os01g0666000
17. Os07g0687900
18. Os05g0161500
19. Os12g0478400
20. Os01g0705200
21. Os06g0521500
22. Os06g0218900
23. Os08g0470200
24. Os07g0643700
25. Os08g0353700
26. Os07g0678300
27. Os02g0205200
28. Os03g0316200
29. Os08g0540900
30. Os07g0468100
31. Os05g0135400
32. Os10g0391400
33. Os04g0605300
34. Os06g0522300
35. Os04g0308300
36. Os02g0646200
37. Os08g0349300
38. Os06g0142200
39. Os10g0488400
40. Os07g0582400
41. Os04g0308401
42. Os01g0802700
43. Os05g0334400
44. Os12g0467700
45. Os10g0517500
46. Os03g0812400
47. Os06g0702000
48. Os04g0308500
49. Os08g0190100
50. Os03g0180900
0h 15' 30' 1h 4h 24h

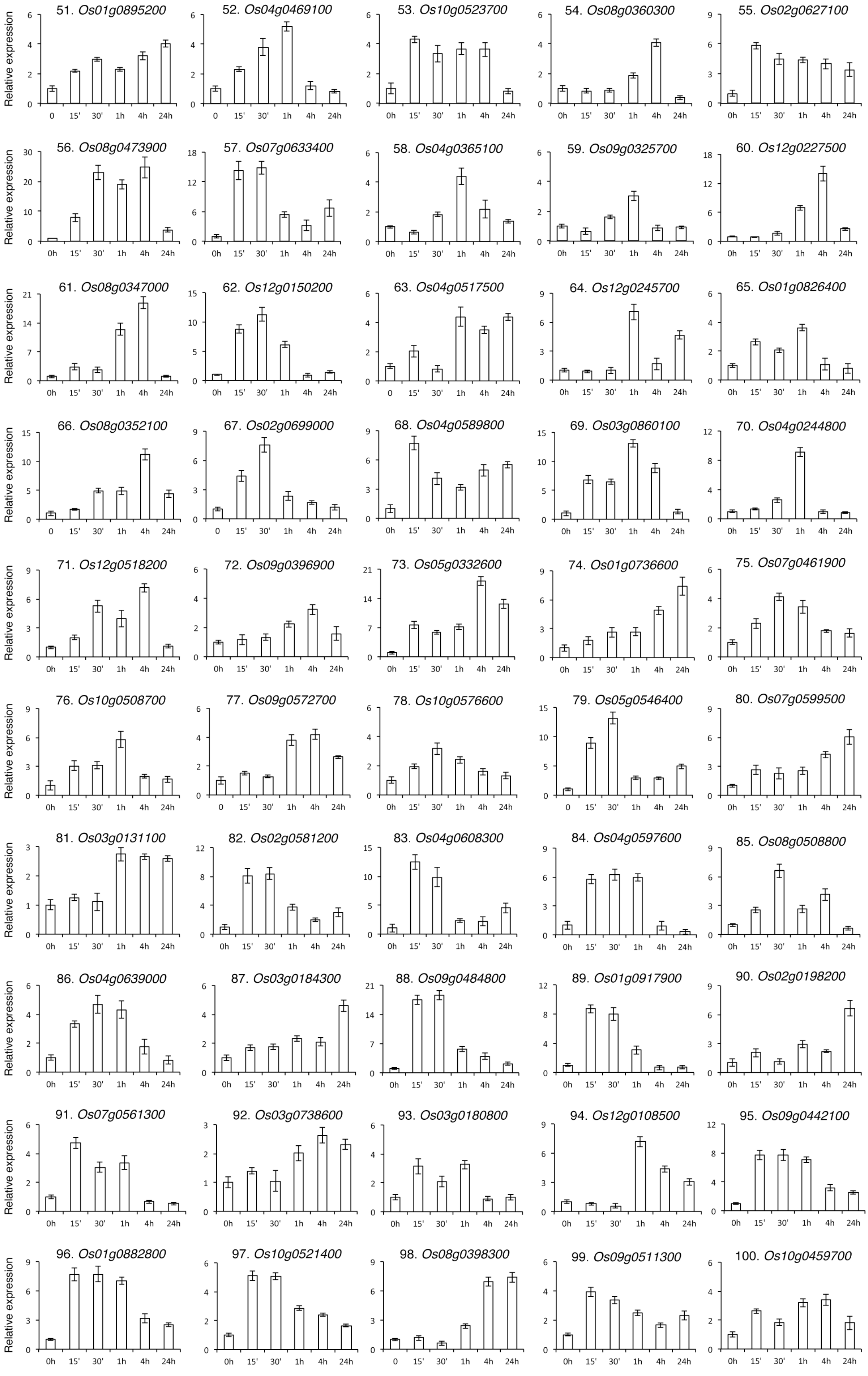
Relative expression
51. Os01g0895200
52. Os04g0469100
53. Os10g0523700
54. Os08g0360300
55. Os02g0627100
56. Os08g0473900
57. Os07g0633400
58. Os04g0365100
59. Os09g0325700
60. Os12g0227500
61. Os08g0347000
62. Os12g0150200
63. Os04g0517500
64. Os12g0245700
65. Os01g0826400
66. Os08g0352100
67. Os02g0699000
68. Os04g0589800
69. Os03g0860100
70. Os04g0244800
71. Os12g0518200
72. Os09g0396900
73. Os05g0332600
74. Os01g0736600
75. Os07g0461900
76. Os10g0508700
77. Os09g0572700
78. Os10g0576600
79. Os05g0546400
80. Os07g0599500
81. Os03g0131100
82. Os02g0581200
83. Os04g0608300
84. Os04g0597600
85. Os08g0508800
86. Os04g0639000
87. Os03g0184300
88. Os09g0484800
89. Os01g0917900
90. Os02g0198200
91. Os07g0561300
92. Os03g0738600
93. Os03g0180800
94. Os12g0108500
95. Os09g0442100
96. Os01g0882800
97. Os10g0521400
98. Os08g0398300
99. Os09g0511300
100. Os10g0459700
0h
15'
30'
1h
4h
24h

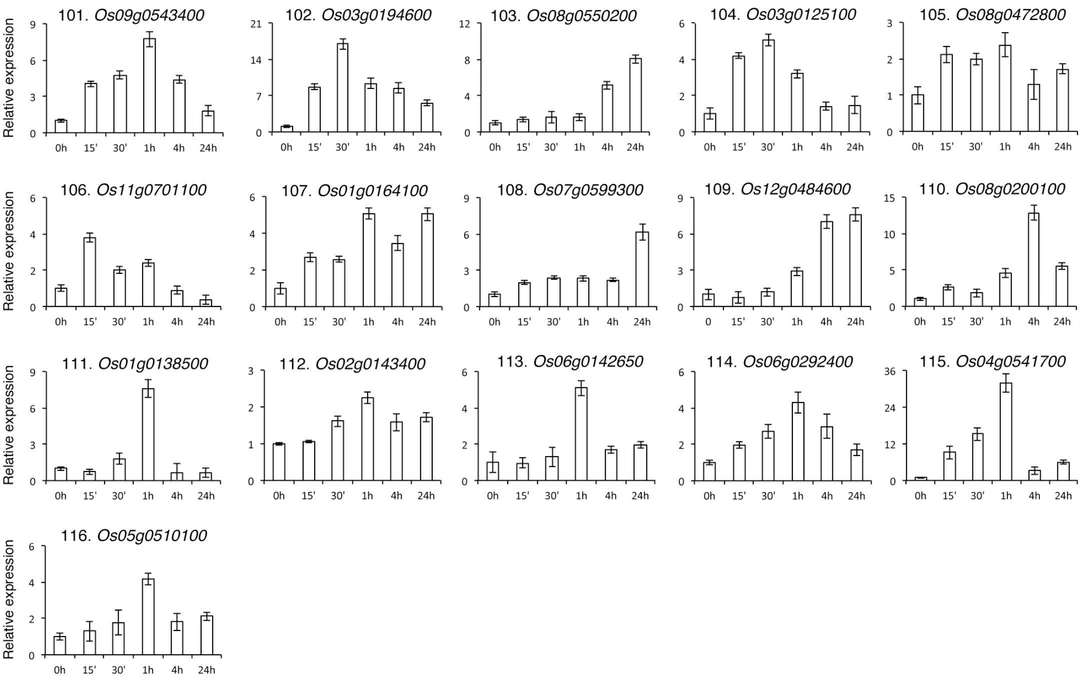

Fig. S3 Quantitative RT-PCR analysis of 116 nitrogen starvation-induced genes. Total RNA extracted from roots of 10-day-old rice seedlings treated with nitrogen starvation for 0–24 h was used for qRT-PCR analysis. Relative expression indicates the fold-change of each gene as compared to that of control. A complete list of the 116 -N-induced genes is shown in Table 1. Data are shown as means ± SD from three biological repeats.

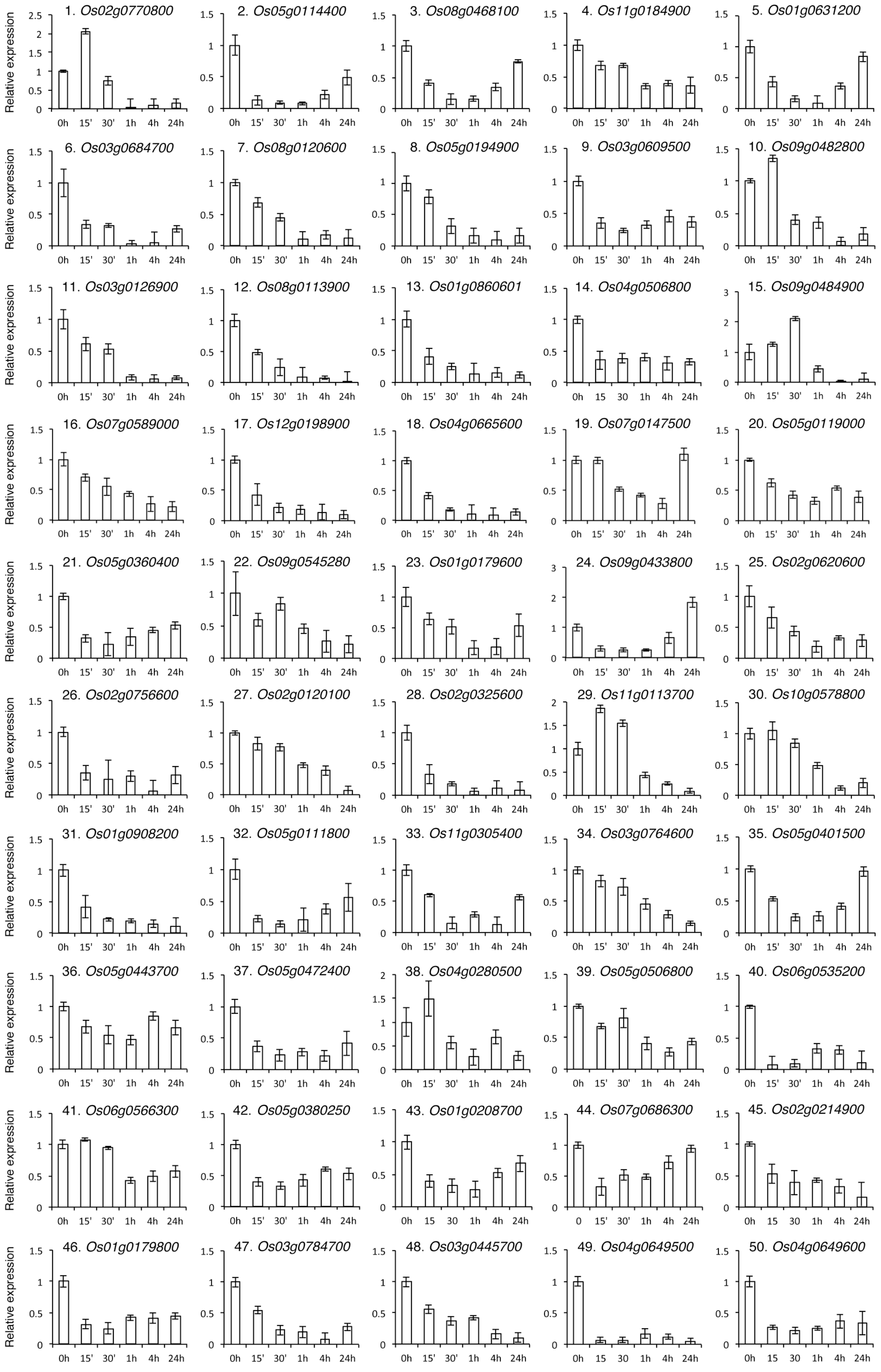
Relative expression
0h 15' 30' 1h 4h 24h
1. Os02g0770800
2. Os05g0114400
3. Os08g0468100
4. Os11g0184900
5. Os01g0631200
6. Os03g0684700
7. Os08g0120600
8. Os05g0194900
9. Os03g0609500
10. Os09g0482800
11. Os03g0126900
12. Os08g0113900
13. Os01g0860601
14. Os04g0506800
15. Os09g0484900
16. Os07g0589000
17. Os12g0198900
18. Os04g0665600
19. Os07g0147500
20. Os05g0119000
21. Os05g0360400
22. Os09g0545280
23. Os01g0179600
24. Os09g0433800
25. Os02g0620600
26. Os02g0756600
27. Os02g0120100
28. Os02g0325600
29. Os11g0113700
30. Os10g0578800
31. Os01g0908200
32. Os05g0111800
33. Os11g0305400
34. Os03g0764600
35. Os05g0401500
36. Os05g0443700
37. Os05g0472400
38. Os04g0280500
39. Os05g0506800
40. Os06g0535200
41. Os06g0566300
42. Os05g0380250
43. Os01g0208700
44. Os07g0686300
45. Os02g0214900
46. Os01g0179800
47. Os03g0784700
48. Os03g0445700
49. Os04g0649500
50. Os04g0649600

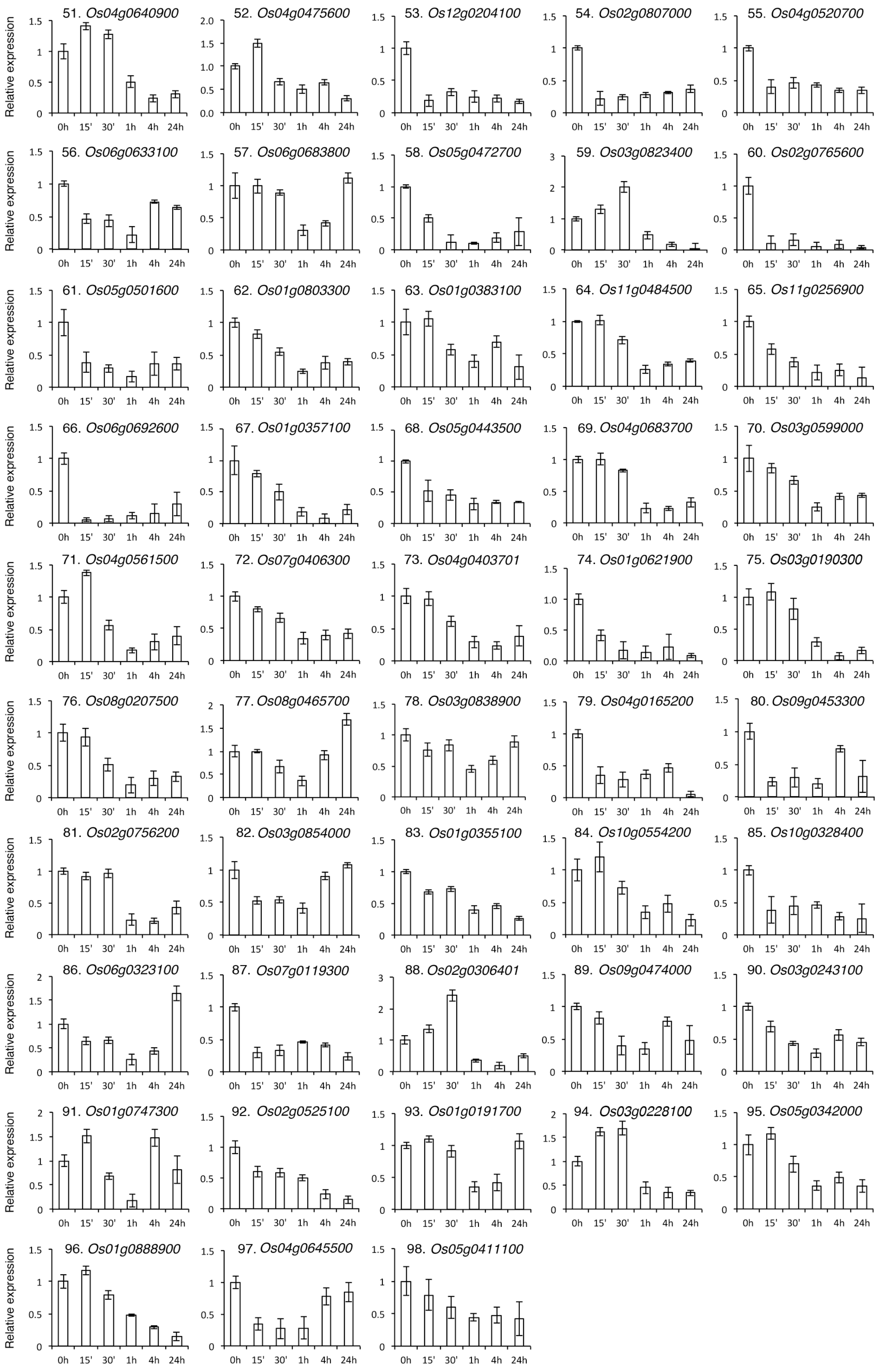

Fig. S4 qRT-PCR analysis of nitrogen starvation-repressed genes in rice roots. A list of these 98 genes is shown in Table 2. Data are means ± SD from three biological repeats.

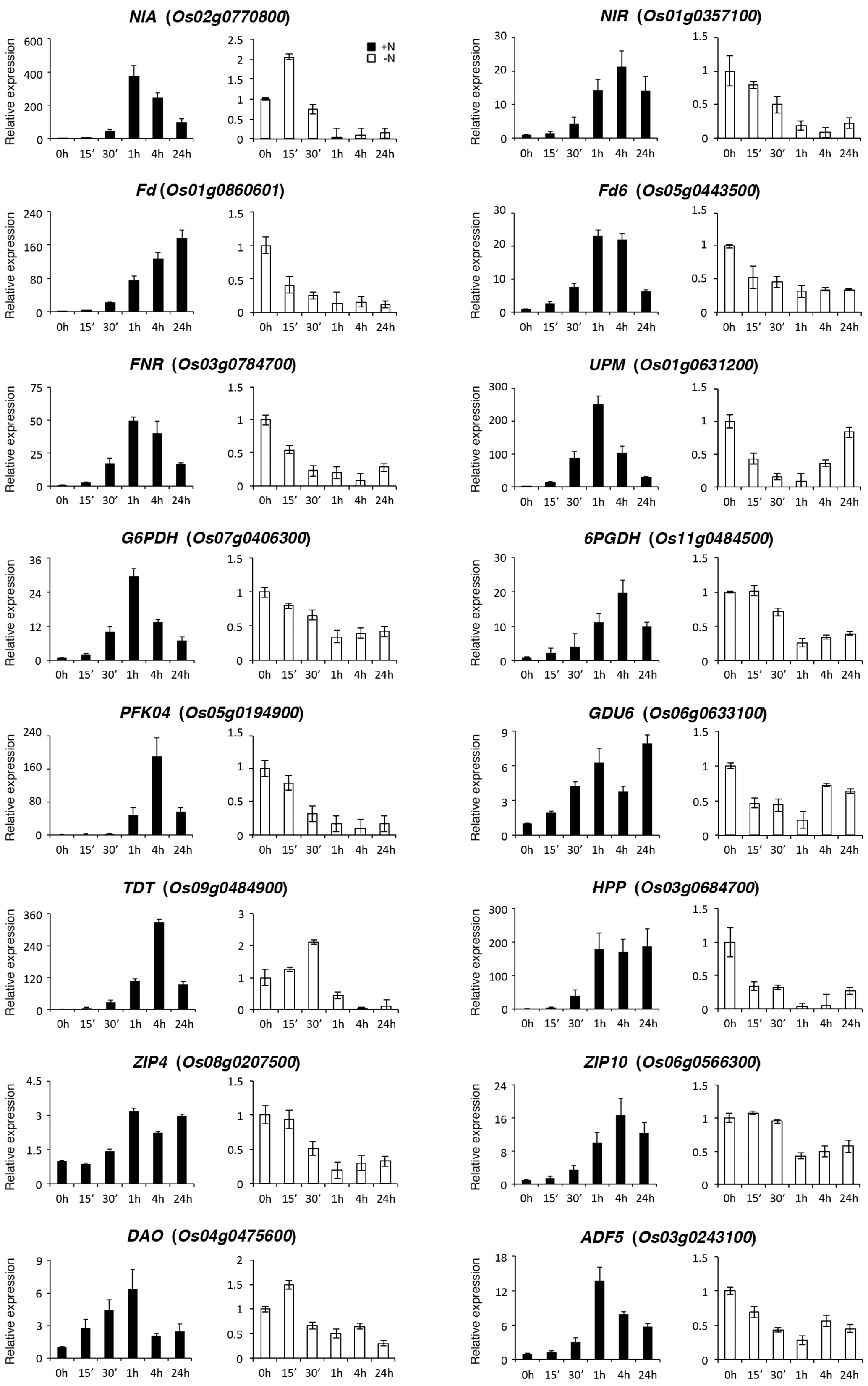
NIA (Os02g0770800)
NIR (Os01g0357100)
Fd (Os01g0860601)
Fd6 (Os05g0443500)
FNR (Os03g0784700)
UPM (Os01g0631200)
G6PDH (Os07g0406300)
6PGDH (Os11g0484500)
PFK04 (Os05g0194900)
GDU6 (Os06g0633100)
TDT (Os09g0484900)
HPP (Os03g0684700)
ZIP4 (Os08g0207500)
ZIP10 (Os06g0566300)
DAO (Os04g0475600)
ADF5 (Os03g0243100)
+N
-N
Relative expression
0h 15’ 30’ 1h 4h 24h

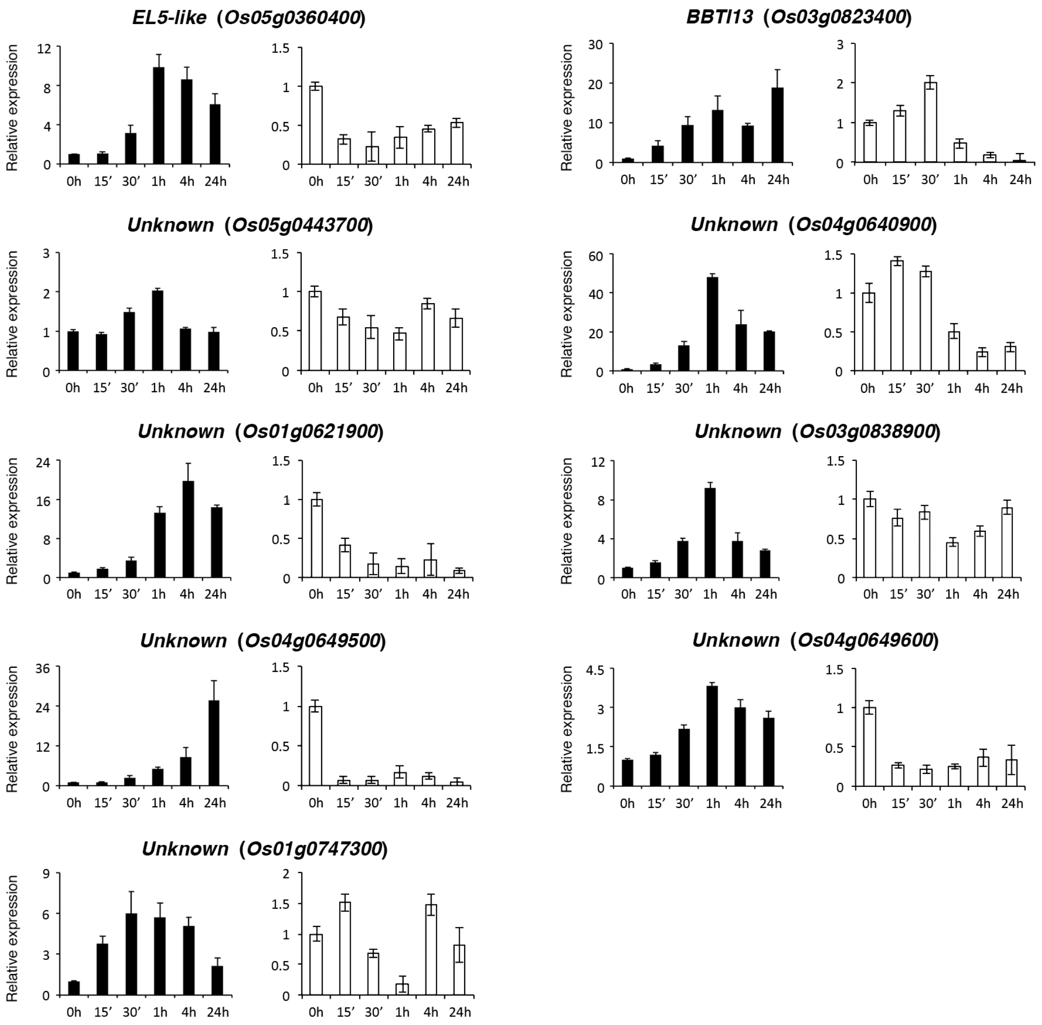

Fig. S5 qRT-PCR analysis of nitrogen-sensitive genes in the roots of 10-day-old rice seedlings. The expression of these genes was rapidly induced by +N and quickly repressed by -N. A complete list of nitrogen-sensitive genes is shown in Table 4. Data are means ± SD from three biological repeats.

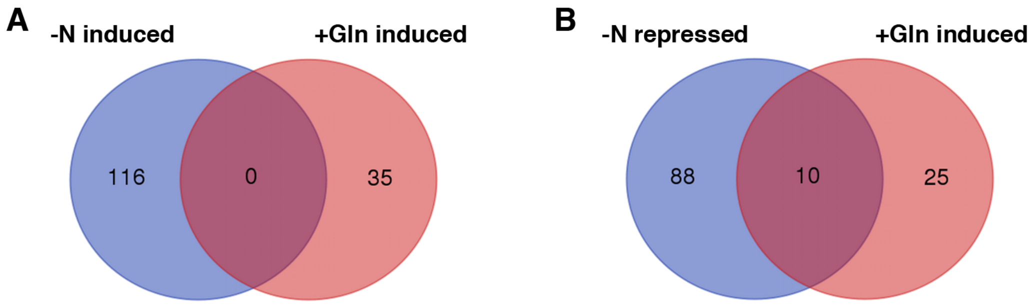

Fig. S6 Venn diagram of genes commonly regulated by nitrogen starvation (-N) and glutamine (+Gln) in rice roots. The +Gln-induced genes were identified by Kan *et al.*[32].

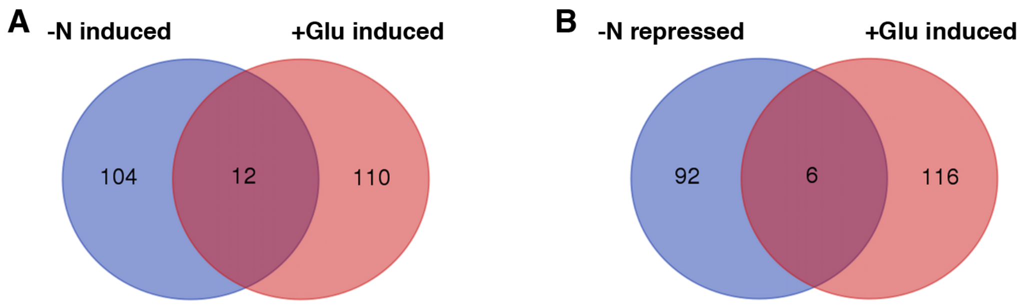

Fig. S7 Venn diagram of genes commonly regulated by nitrogen starvation (-N) and glutamate (+Glu) in rice roots. The +Glu-regulated genes were identified by Kan *et al.*[33].

Table S1 Gene ontology (GO) enrichment analysis of nitrogen starvation-induced genes in rice roots

Locus ID		Gene Description
**Biological Process/small molecule metabolic process (GO:0044281)**		
Os02g0626600	LOC_Os02g41670	Phenylalanine ammonia-lyase 3 (PAL3)
Os02g0627100	LOC_Os02g41680	Phenylalanine ammonia-lyase 4 (PAL4)
Os05g0161500	LOC_Os05g06920	GTP diphosphokinase; Calcium-activated RelA-SpoT homolog 2 (CRSH2)
Os07g0461900	LOC_Os07g27780	Acetylornithine aminotransferase (ACOAT)
Os08g0200100	LOC_Os08g10010	Acyl-[acyl-carrier-protein] desaturase 7; AtSAD2 homolog
Os08g0470200	LOC_Os08g36630	Alpha carbonic anhydrase 7 (α-CA7)
Os09g0543400	LOC_Os09g37120	Ornithine decarboxylase 1 (ODC1)
Os10g0517500	LOC_Os10g37340	Methionine gamma-lyase (MGL)
Os10g0523700	LOC_Os10g37980	Arogenate dehydratase/prephenate dehydratase 6 (ADT6)
**cellular amino acid and derivative metabolic process (GO:0006519), cellular nitrogen compound metabolic process (GO:0034641), amine metabolic process (GO:0006082), cellular amine metabolic process (GO:0044106)**		
Os02g0626600	LOC_Os02g41670	Phenylalanine ammonia-lyase 3 (PAL3)
Os02g0627100	LOC_Os02g41680	Phenylalanine ammonia-lyase 4 (PAL4)
Os07g0461900	LOC_Os07g27780	Acetylornithine aminotransferase (ACOAT)
Os09g0543400	LOC_Os09g37120	Ornithine decarboxylase 1 (ODC1)
Os10g0517500	LOC_Os10g37340	Methionine gamma-lyase (MGL)
Os10g0523700	LOC_Os10g37980	Arogenate dehydratase/prephenate dehydratase 6 (ADT6)
**cellular amino acid metabolic process (GO:0044106)**		
Os02g0626600	LOC_Os02g41670	Phenylalanine ammonia-lyase 3 (PAL3)
Os02g0627100	LOC_Os02g41680	Phenylalanine ammonia-lyase 4 (PAL4)
Os07g0461900	LOC_Os07g27780	Acetylornithine aminotransferase (ACOAT)
Os10g0517500	LOC_Os10g37340	Methionine gamma-lyase (MGL)
Os10g0523700	LOC_Os10g37980	Arogenate dehydratase/prephenate dehydratase 6 (ADT6)
**cellular ketone metabolic process (GO:0042180), organic acid metabolic process (GO:0006082), oxoacid metabolic process (GO:0043436), carboxylic acid metabolic process (GO:0019752)**		
Os02g0626600	LOC_Os02g41670	Phenylalanine ammonia-lyase 3 (PAL3)
Os02g0627100	LOC_Os02g41680	Phenylalanine ammonia-lyase 4 (PAL4)
Os07g0461900	LOC_Os07g27780	Acetylornithine aminotransferase (ACOAT)
Os08g0200100	LOC_Os08g10010	Acyl-[acyl-carrier-protein] desaturase 7; AtSAD2 homolog
Os10g0517500	LOC_Os10g37340	Methionine gamma-lyase (MGL)

Os10g0523700	LOC_Os10g37980	Arogenate dehydratase/prephenate dehydratase 6 (ADT6)

Molecular Function/ion binding (GO:0043167), cation binding (GO:0043169)

Os01g0736600	LOC_Os01g53500	RING-H2 finger protein ATL67
Os01g0917900	LOC_Os01g68900	C3HC4 type zinc finger protein no-on-transient A (NONA)
Os02g0198200	LOC_Os02g10470	Calcium-binding protein CML21
Os02g0646200	LOC_Os02g43170	B-box zinc finger protein 6 (BBX6)
Os03g0738600	LOC_Os03g52860	Linoleate 9S-lipoxygenase 2; AtLOX1 homolog
Os03g0812400	LOC_Os03g59770	Calmodulin-like protein 2
Os04g0244800	LOC_Os04g17100	Heavy metal-associated isoprenylated plant protein 26
Os04g0365100	LOC_Os04g29580	Wall-associated receptor kinase 37 (WAK37)
Os05g0135400	LOC_Os05g04490	Peroxidase 5
Os05g0161500	LOC_Os05g06920	GTP diphosphokinase; Calcium-activated RelA-SpoT homolog 2 (CRSH2)
Os06g0521500	LOC_Os06g32990	Peroxidase 2-like
Os06g0522300	LOC_Os06g33100	Peroxidase 2-like
Os08g0137800	LOC_Os08g04340	Mavicyanin, phytocyanin
Os08g0190100	LOC_Os08g09080	Germin-like protein 8-11
Os08g0200100	LOC_Os08g10010	Acyl-[acyl-carrier-protein] desaturase 7; AtSAD2 homolog
Os08g0470200	LOC_Os08g36630	Alpha carbonic anhydrase 7 (α-CA7)
Os08g0472800	LOC_Os08g36860	Abscisic acid 8'-hydroxylase 2
Os08g0473900	LOC_Os08g36910	Alpha-amylase isozyme 3D
Os08g0508800	LOC_Os08g39840	Lipoxygenase 7, chloroplastic; AtLOX2 homolog
Os09g0572700	LOC_Os09g39940	Basic blue protein; phytocyanin
Os11g0701100	LOC_Os11g47520	Xylanase inhibitor protein 2
Os12g0150200	LOC_Os12g05440	Cytochrome P450 94C1

metal ion binding (GO:0046872)

Os01g0736600	LOC_Os01g53500	RING-H2 finger protein ATL67
Os01g0917900	LOC_Os01g68900	C3HC4 type zinc finger protein no-on-transient A (NONA)
Os02g0198200	LOC_Os02g10470	Calcium-binding protein CML21
Os02g0646200	LOC_Os02g43170	B-box zinc finger protein 6 (BBX6)
Os03g0738600	LOC_Os03g52860	Linoleate 9S-lipoxygenase 2; AtLOX1 homolog
Os03g0812400	LOC_Os03g59770	Calmodulin-like protein 2
Os04g0244800	LOC_Os04g17100	Heavy metal-associated isoprenylated plant protein 26
Os04g0365100	LOC_Os04g29580	Wall-associated receptor kinase 37 (WAK37)
Os05g0135400	LOC_Os05g04490	Peroxidase 5
Os05g0161500	LOC_Os05g06920	GTP diphosphokinase; Calcium-activated RelA-SpoT homolog 2

		(CRSH2)
Os06g0521500	LOC_Os06g32990	Peroxidase 2-like
Os06g0522300	LOC_Os06g33100	Peroxidase 2-like
Os08g0137800	LOC_Os08g04340	Mavicyanin, phytocyanin
Os08g0190100	LOC_Os08g09080	Germin-like protein 8-11
Os08g0200100	LOC_Os08g10010	Acyl-[acyl-carrier-protein] desaturase 7; AtSAD2 homolog
Os08g0470200	LOC_Os08g36630	Alpha carbonic anhydrase 7 (α-CA7)
Os08g0472800	LOC_Os08g36860	Abscisic acid 8'-hydroxylase 2
Os08g0473900	LOC_Os08g36910	Alpha-amylase isozyme 3D
Os08g0508800	LOC_Os08g39840	Lipoxygenase 7, chloroplastic; AtLOX2 homolog
Os09g0572700	LOC_Os09g39940	Basic blue protein; phytocyanin
Os12g0150200	LOC_Os12g05440	Cytochrome P450 94C1
**iron ion binding (GO:0005506)**		
Os03g0738600	LOC_Os03g52860	Linoleate 9S-lipoxygenase 2; AtLOX1 homolog
Os05g0135400	LOC_Os05g04490	Peroxidase 5
Os06g0521500	LOC_Os06g32990	Peroxidase 2-like
Os06g0522300	LOC_Os06g33100	Peroxidase 2-like
Os08g0472800	LOC_Os08g36860	Abscisic acid 8'-hydroxylase 2
Os08g0508800	LOC_Os08g39840	Lipoxygenase 7, chloroplastic; AtLOX2 homolog
Os12g0150200	LOC_Os12g05440	Cytochrome P450 94C1

Table S2 KEGG pathway enrichment analysis of genes rapidly induced by nitrogen starvation in rice roots

Locus ID		Gene Description
**Plant hormone signal transduction (4.92E-5)**		
Os10g0391400	LOC_Os10g25230	TIFY 11e; jasmonate ZIM domain-containing protein 13 (JAZ13)
Os03g0180900	LOC_Os03g08320	TIFY 11c; jasmonate ZIM domain-containing protein 11 (JAZ11)
Os09g0325700	LOC_Os09g15670	Protein phosphatase 2C 68 (PP2C68)
Os03g0860100	LOC_Os03g64260	Ethylene-responsive transcription factor 15 (ERF15)
Os04g0608300	LOC_Os04g51890	Auxin-responsive protein SAUR36-like
Os03g0180800	LOC_Os03g08310	TIFY 11a; jasmonate ZIM domain-containing protein 9 (JAZ9)
Os02g0143400	LOC_Os02g05060	Auxin-induced protein X15
**Carotenoid biosynthesis (3.96E-4)**		
Os09g0555500	LOC_Os09g38320	Phytoene synthase 3 (PSY3)
Os03g0125100	LOC_Os03g03370	Beta-carotene hydroxylase 1 (BCH1); HYD3
Os08g0472800	LOC_Os08g36860	Abscisic acid 8'-hydroxylase 2
**Plant-pathogen interaction (5.53E-4)**		
Os10g0391400	LOC_Os10g25230	TIFY 11e; jasmonate ZIM domain-containing protein 13 (JAZ13)
Os03g0180900	LOC_Os03g08320	TIFY 11c; jasmonate ZIM domain-containing protein 11 (JAZ11)
Os01g0826400	LOC_Os01g61080	WRKY transcription factor 33 (WRKY33)
Os02g0198200	LOC_Os02g10470	Calcium-binding protein CML21
Os03g0180800	LOC_Os03g08310	TIFY 11a; jasmonate ZIM domain-containing protein 9 (JAZ9)
**Linoleic acid metabolism (2.57E-3)**		
Os08g0508800	LOC_Os08g39840	Lipoxygenase 7, chloroplastic; AtLOX2 homolog
Os03g0738600	LOC_Os03g52860	Linoleate 9S-lipoxygenase 2; AtLOX1 homolog
**Arginine and proline metabolism (0.05)**		
Os07g0461900	LOC_Os07g27780	Acetylornithine aminotransferase (ACOAT)
Os09g0543400	LOC_Os09g37120	Ornithine decarboxylase 1 (ODC1)

The 116 genes induced by nitrogen starvation were used for KEGG pathway enrichment analysis (http://expath.itps.ncku.edu.tw) with the thresholds of p-value < 0.05 indicated in the parenthesis.

Table S3 Gene ontology enrichment analysis of nitrogen starvation-repressed genes in rice roots

Locus ID		Gene Description
**Biological Process/localization (GO:0051179), establishment of localization (GO:0051234), transport (GO:0006810)**		
Os01g0383100	LOC_Os01g28600	Exocyst complex component EXO70A1
Os02g0620600	LOC_Os02g40730	Ammonium transporter 1 member 2 (AMT1;2)
Os05g0411100	LOC_Os05g34030	NRT1/ PTR FAMILY 3.1-like (NPF)
Os05g0443700	LOC_Os05g37150	Syntaxin 6, N-terminal domain-containing protein
Os05g0472400	LOC_Os05g39540	Zinc transporter 9 (ZIP9)
Os05g0472700	LOC_Os05g39560	Zinc transporter 5 (ZIP5)
Os06g0566300	LOC_Os06g37010	Zinc transporter 10 (ZIP10)
Os08g0207500	LOC_Os08g10630	Zinc transporter 4 (ZIP4)
Os09g0484900	LOC_Os09g31130	Tonoplast dicarboxylate transporter (TDT)
Os10g0554200	LOC_Os10g40600	NRT1/PTR FAMILY 6.3 (NPF6.5)
Os12g0204100	LOC_Os12g10280	Aquaporin NIP3;5
**ion transport (GO:0006811), cation transport (GO:0006812), metal ion transport (GO:0030001)**		
Os05g0472400	LOC_Os05g39540	Zinc transporter 9 (ZIP9)
Os05g0472700	LOC_Os05g39560	Zinc transporter 5 (ZIP5)
Os06g0566300	LOC_Os06g37010	Zinc transporter 10 (ZIP10)
Os08g0207500	LOC_Os08g10630	Zinc transporter 4 (ZIP4)
Os09g0484900	LOC_Os09g31130	Tonoplast dicarboxylate transporter (TDT)
**small molecule metabolic process (GO:0044281)**		
Os01g0191700	LOC_Os01g09570	ATP-dependent 6-phosphofructokinase (PFK01)
Os02g0306401	LOC_Os02g20360	Nicotianamine aminotransferase A
Os05g0194900	LOC_Os05g10650	ATP-dependent 6-phosphofructokinase 4 (PFK04)
Os07g0406300	LOC_Os07g22350	Glucose-6-phosphate dehydrogenase (G6PDH)
Os08g0120600	LOC_Os08g02700	Fructose-bisphosphate aldolase
Os10g0554200	LOC_Os10g40600	NRT1/ PTR FAMILY 6.3 (NPF6.5)
Os11g0484500	LOC_Os11g29400	6-phosphogluconate dehydrogenase (6PGDH)
**alcohol metabolic process (GO:0006066), monosaccharide metabolic process (GO:0005996), hexose metabolic process (GO:0019318), glucose metabolic process (GO:0006006)**		
Os08g0120600	LOC_Os08g02700	Fructose-bisphosphate aldolase
Os01g0191700	LOC_Os01g09570	ATP-dependent 6-phosphofructokinase (PFK01)
Os07g0406300	LOC_Os07g22350	Glucose-6-phosphate dehydrogenase (G6PDH)
Os05g0194900	LOC_Os05g10650	ATP-dependent 6-phosphofructokinase 4 (PFK04)

Os11g0484500	LOC_Os11g29400	6-phosphogluconate dehydrogenase (6PGDH)

cellular carbohydrate metabolic process (GO:0044262)

Os05g0194900	LOC_Os05g10650	ATP-dependent 6-phosphofructokinase 4 (PFK04)
Os08g0120600	LOC_Os08g02700	Fructose-bisphosphate aldolase
Os01g0191700	LOC_Os01g09570	ATP-dependent 6-phosphofructokinase 1 (PFK01)
Os11g0484500	LOC_Os11g29400	6-phosphogluconate dehydrogenase (6PGDH)
Os07g0406300	LOC_Os07g22350	Glucose-6-phosphate dehydrogenase (G6PDH)
Os04g0506800	LOC_Os04g42760	Sialyltransferase-like protein 3 (STLP3)

catabolic process (GO:0009056), cellular catabolic process (GO:0044248)

Os01g0208700	LOC_Os01g11054	phosphoenolpyruvate carboxylase 4 (PPC4)
Os08g0120600	LOC_Os08g02700	Fructose-bisphosphate aldolase
Os01g0191700	LOC_Os01g09570	ATP-dependent 6-phosphofructokinase 1 (PFK01)
Os05g0194900	LOC_Os05g10650	ATP-dependent 6-phosphofructokinase 4 (PFK04)
Os11g0484500	LOC_Os11g29400	6-phosphogluconate dehydrogenase (6PGDH)

Molecular Function/transporter activity (GO:0005215)

Os02g0620600	LOC_Os02g40730	Ammonium transporter 1 member 2 (AMT1;2)
Os05g0411100	LOC_Os05g34030	NRT1/PTR FAMILY 3.1-like
Os05g0472400	LOC_Os05g39540	Zinc transporter 9 (ZIP9)
Os05g0472700	LOC_Os05g39560	Zinc transporter (ZIP5)
Os06g0566300	LOC_Os06g37010	Zinc transporter (ZIP10)
Os08g0207500	LOC_Os08g10630	Zinc transporter (ZIP4)
Os09g0484900	LOC_Os09g31130	Tonoplast dicarboxylate transporter (TDT)
Os10g0554200	LOC_Os10g40600	NRT1/PTR FAMILY 6.3 (NPF6.5)
Os12g0204100	LOC_Os12g10280	Aquaporin NIP3;5

cation transmembrane transporter activity (GO:0008324), inorganic cation transmembrane transporter activity (GO:0022890)

Os02g0620600	LOC_Os02g40730	Ammonium transporter 1 member 2 (AMT1;2)
Os05g0472400	LOC_Os05g39540	Zinc transporter 9 (ZIP9)
Os05g0472700	LOC_Os05g39560	Zinc transporter (ZIP5)
Os06g0566300	LOC_Os06g37010	Zinc transporter (ZIP10)
Os08g0207500	LOC_Os08g10630	Zinc transporter (ZIP4)

Cellular Component/cell (GO:0005623), cell part (GO:0044464)

Os05g0114400	LOC_Os05g02390	Zinc finger protein, ZOS5-02
Os10g0328400	LOC_Os10g18099	Unknown

Os03g0784700	LOC_Os03g57120	Ferredoxin--NADP reductase (FNR)
Os09g0474000	LOC_Os09g29820	bZIP transcription factor 53
Os07g0147500	LOC_Os07g05360	Photosystem II 10 kDa polypeptide, PsbR
Os05g0411100	LOC_Os05g34030	NRT1/PTR FAMILY 3.1-like (NPF)
Os06g0566300	LOC_Os06g37010	Zinc transporter 10 (ZIP10)
Os05g0194900	LOC_Os05g10650	ATP-dependent 6-phosphofructokinase 4 (PFK04)
Os04g0165200	LOC_Os04g08290	Zinc finger protein STAR3-like, ZOS4-04
Os01g0191700	LOC_Os01g09570	ATP-dependent 6-phosphofructokinase 1 (PFK01)
Os01g0383100	LOC_Os01g28600	Exocyst complex component EXO70A1
Os01g0179800	LOC_Os01g08460	Probable serine incorporator (Serinc)
Os09g0484900	LOC_Os09g31130	Tonoplast dicarboxylate transporter (TDT)
Os08g0207500	LOC_Os08g10630	Zinc transporter 4 (ZIP4)
Os05g0443700	LOC_Os05g37150	Syntaxin 6, N-terminal domain-containing protein
Os03g0243100	LOC_Os03g13950	Actin-depolymerizing factor 5 (ADF5)
Os01g0908200	LOC_Os01g68020	BTB/POZ and TAZ domain-containing protein 2 (BT2)
Os02g0214900	LOC_Os02g12350	Histone deacetylase 3 (HDAC3)
Os12g0204100	LOC_Os12g10280	Aquaporin NIP3;5
Os10g0554200	LOC_Os10g40600	NRT1/PTR FAMILY 6.3 (NPF6.5)
Os05g0472400	LOC_Os05g39540	Zinc transporter 9 (ZIP9)
Os10g0578800	LOC_Os10g42780	Plastidal glycolate/glycerate translocator 1 (PLGG1)
Os02g0620600	LOC_Os02g40730	Ammonium transporter 1 member 2 (AMT1;2)
Os05g0472700	LOC_Os05g39560	Zinc transporter 5 (ZIP5)
Os01g0803300	LOC_Os01g58910	EamA domain-containig drug/metabolite transporter (DMT)
Os04g0506800	LOC_Os04g42760	Sialyltransferase-like protein 3 (STLP3)

membrane (GO:0016020)

Os01g0179800	LOC_Os01g08460	Probable serine incorporator (Serinc)
Os01g0803300	LOC_Os01g58910	EamA domain-containig drug/metabolite transporter (DMT)
Os02g0620600	LOC_Os02g40730	Ammonium transporter 1 member 2 (AMT1;2)
Os03g0784700	LOC_Os03g57120	Ferredoxin--NADP reductase (FNR)
Os04g0506800	LOC_Os04g42760	Sialyltransferase-like protein 3 (STLP3)
Os05g0411100	LOC_Os05g34030	NRT1/PTR FAMILY 3.1-like (NPF)
Os05g0443700	LOC_Os05g37150	Syntaxin 6, N-terminal domain-containing protein
Os05g0472400	LOC_Os05g39540	Zinc transporter 9 (ZIP9)
Os05g0472700	LOC_Os05g39560	Zinc transporter 5 (ZIP5)
Os06g0566300	LOC_Os06g37010	Zinc transporter 10 (ZIP10)
Os07g0147500	LOC_Os07g05360	photosystem II 10 kDa polypeptide, chloroplastic
Os08g0207500	LOC_Os08g10630	Zinc transporter 4 (ZIP4)

Os09g0484900	LOC_Os09g31130	Tonoplast dicarboxylate transporter (TDT)
Os10g0554200	LOC_Os10g40600	NRT1/PTR FAMILY 6.3 (NPF6.5)
Os10g0578800	LOC_Os10g42780	Plastidal glycolate/glycerate translocator 1 (PLGG1)
Os12g0204100	LOC_Os12g10280	Aquaporin NIP3;5

Table S4 KEGG pathway enrichment analysis of genes rapidly repressed by nitrogen starvation in rice roots

Pathway (P-value)	Gene Identifier		Gene Description
Pentose phosphate pathway (1.79E-5)	Os08g0120600	LOC_Os08g02700	Fructose-bisphosphate aldolase
	Os11g0484500	LOC_Os11g29400	6-phosphogluconate dehydrogenase (6PGDH)
	Os07g0406300	LOC_Os07g22350	Glucose-6-phosphate dehydrogenase (G6PDH)
	Os01g0191700	LOC_Os01g09570	ATP-dependent 6-phosphofructokinase 1 (PFK01)
Photosynthesis (2.99E-5)	Os01g0860601	LOC_Os01g64120	Ferredoxin, root R-B1
	Os07g0147500	LOC_Os07g05360	Photosystem II 10 kDa polypeptide, PsbR
	Os03g0784700	LOC_Os03g57120	Ferredoxin--NADP reductase (FNR)
	Os05g0443500	LOC_Os05g37140	Ferredoxin 6, chloroplastic
Nitrogen metabolism (1.46E-4)	Os02g0770800	LOC_Os02g53130	Nitrate reductase (NAD(P)H)
	Os08g0468100	LOC_Os08g36480	Nitrate reductase (NADH) 1
	Os01g0357100	LOC_Os01g25484	Nitrite reductase
Carbon metabolism (8.98E-4)	Os08g0120600	LOC_Os08g02700	Fructose-bisphosphate aldolase
	Os01g0208700	LOC_Os01g11054	Phosphoenolpyruvate carboxylase 4 (PPC4)
	Os11g0484500	LOC_Os11g29400	6-phosphogluconate dehydrogenase (6PGDH)
	Os07g0406300	LOC_Os07g22350	Glucose-6-phosphate dehydrogenase (G6PDH)
	Os01g0191700	LOC_Os01g09570	ATP-dependent 6-phosphofructokinase 1 (PFK01)
Fructose and mannose metabolism (0.02)	Os08g0120600	LOC_Os08g02700	Fructose-bisphosphate aldolase
	Os01g0191700	LOC_Os01g09570	ATP-dependent 6-phosphofructokinase 1 (PFK01)
Carbon fixation in photosynthetic organisms (0.03)	Os08g0120600	LOC_Os08g02700	Fructose-bisphosphate aldolase
	Os01g0208700	LOC_Os01g11054	Phosphoenolpyruvate carboxylase 4 (PPC4)
Glutathione metabolism (0.03)	Os11g0484500	LOC_Os11g29400	6-phosphogluconate dehydrogenase (6PGDH)
	Os07g0406300	LOC_Os07g22350	Glucose-6-phosphate 1-dehydrogenase (G6PDH)
Biosynthesis of amino acids (0.04)	Os08g0120600	LOC_Os08g02700	Fructose-bisphosphate aldolase
	Os02g0306401	LOC_Os02g20360	Nicotianamine aminotransferase A
	Os01g0191700	LOC_Os01g09570	ATP-dependent 6-phosphofructokinase 1 (PFK01)

Table S5 List of genes rapidly repressed by nitrogen starvation and induced by glutamine in rice roots

No.	Locus ID		Fold change		Gene description
			-N/+N	+Gln/-N	
1	Os05g0114400	LOC_Os05g02390	-13	5.4	Zinc finger transcription factor, ZOS5-02
2	Os11g0184900	LOC_Os11g08210	-7.4	4.0	NAC domain-containing protein 5 (NAC5)
3	Os05g0194900	LOC_Os05g10650	-6.2	2.4	ATP-dependent 6-phosphofructokinase 4 (PFK04)
4	Os09g0482800	LOC_Os09g30490	-5.9	3.2	EF-hand domain-containing protein
5	Os07g0589000	LOC_Os07g40000	-4.8	2.3	LOB domain-containing protein 37 (LBD37)
6	Os12g0113500	LOC_Os12g02200	-3.5	2.3	CBL-interacting protein kinase 14 (CIPK14)
7	Os03g0445700	LOC_Os03g33090	-2.7	2.7	LOB domain-containing protein 37 (LBD37)
8	Os06g0633100	LOC_Os06g42660	-2.6	3.6	Glutamine dumper 6 (GDU6)
9	Os03g0823400	LOC_Os03g60840	-2.6	3.5	Bowman-Birk type trypsin inhibitor (BBTI)
10	Os07g0119300	LOC_Os07g02800	-2.1	2.6	MYB family protein

Table S6 List of genes rapidly induced by nitrogen starvation and glutamate in rice roots

No.	Locus ID		Fold change		Gene description
			-N/+N	+Glu/-N	
1	Os03g0318400	LOC_Os03g20290	3.5	3.6	Aspartic proteinase nepenthesin-1
2	Os09g0455300	LOC_Os09g28210	3.4	2.4	Basic helix-loop-helix transcription factor (bHLH120)
3	Os01g0666000	LOC_Os01g47580	3.4	3.6	Lipid phosphate phosphatase 2
4	Os12g0478400	LOC_Os12g29430	3.2	2.8	Wall-associated receptor kinase 125 (WAK125)
5	Os01g0705200	LOC_Os01g50910	3.2	3.7	Late embryogenesis abundant protein, group 3
6	Os02g0627100	LOC_Os02g41680	2.4	2.2	Phenylalanine ammonia-lyase 4 (PAL4)
7	Os08g0473900	LOC_Os08g36910	2.4	2.7	Alpha amylase isozyme 3D
8	Os12g0518200	LOC_Os12g33300	2.2	5.1	EamA domain-containig drug/metabolite transporter (DMT)
9	Os03g0194600	LOC_Os03g09880	2.1	2.2	Cytochrome b561 and DOMON domain-containing protein
10	Os08g0508800	LOC_Os08g39840	2.1	5.4	Lipoxygenase 7; AtLOX2 homolog
11	Os01g0882800	LOC_Os01g66010	2.1	2.1	Amino acid permease 8 (AAP8)
12	Os06g0292400	LOC_Os06g18900	2.0	2.0	Unknown

Table S7 List of genes rapidly repressed by nitrogen starvation and induced by glutamate in rice roots

No.	Locus ID		Fold change		Gene description
			-N/+N	+Glu/-N	
1	Os11g0184900	LOC_Os11g08210	-7.4	2.0	NAC domain-containing protein 5 (NAC5)
2	Os09g0482800	LOC_Os09g30490	-5.9	2.1	EF-hand domain-containing protein
3	Os09g0484900	LOC_Os09g31130	-5	2.3	Tonoplast dicarboxylate transporter (TDT)
4	Os07g0589000	LOC_Os07g40000	-4.8	2.0	LOB domain-containing protein 37 (LBD37)
5	Os03g0823400	LOC_Os03g60840	-2.6	4.7	Bowman-Birk type trypsin inhibitor (BBTI)
6	Os07g0119300	LOC_Os07g02800	-2.1	2.1	MYB family protein

Table S8. Sequences of primers used for qRT-PCR analysis of N starvation-induced genes

No.	Locus ID	Primer sequence 5'→3'
1	Os12g0189300	TTCTTTATCGTGGCCCGGA/GGCATCATCAAGACCAGTGACA
2	Os12g0156100	TCGACTGGAGCTCGCTGATTA/GATGTCGTCGTTGAAGCTGAAA
3	Os06g0725200	GCCCAATTAACCAAGCCCAT/CGTAATTAAGCAGTGGTGCGG
4	Os08g0137800	TTTCGTGCCGGAGATCAGATAG/TTCACCTCCACCACATCATGC
5	Os07g0127500	GATCGATCTGCAATAATGGCG/CAGTAGCACCAGGACAAAGCCT
6	Os03g0667500	TCTGCGGCATCATGCAATT/TGATCGAGTCTGACCAAGCTCA
7	Os02g0626600	ACAACACTCGCCTCGCAATT/CTCTGAGAACTGCGCGAACAT
8	Os09g0555500	CGAGGCCAACGATTACAACAA/GCCACAATCTTCTTTGCCTTTG
9	Os09g0543900	TCGATCTTCGACAAGGTGACGT/ACGCGTAGATGATCGCCATCT
10	Os06g0587401	AATAAAGACGTAGCCGGCGTC/GGCCTACTGCCCCATAATTGT
11	Os01g0595600	CGGTCAACGACGGCTATAAGA/TACATGACGACCCAAACACGAA
12	Os12g0556300	TGCAATGGTGTGGCGTTTTA/ATTAGAGGTGCTGCAGTCGCA
13	Os03g0318400	TTGCCATTATTGAAGGCGGA/TTCTGCTGCTGGAAATTGCC
14	Os03g0183500	TTACGCCATGATTCCGAGAG/CCACAGCTCCATCCTCGATAT
15	Os09g0455300	GCTCCGAGCTTTTGTTGGACT/TACGGTCGAATGAAACGCG
16	Os01g0666000	CCGGCTCCTTCAGATGAGAAA/CGGTGATGTACCTAAAGTGCGC
17	Os07g0687900	TTCGAAGCCATGGAGGTTCA/CACCACCTCTTCACCAGCATCT
18	Os05g0161500	AACGAGCTGCAAAATCGATCTG/CTTCAAATTGACGCCGAAACC
19	Os12g0478400	AAATCCCTACGTTGCTGGTGG/TAATCATCCGAGCGAGCACAC
20	Os01g0705200	ATAACAAGAACAACGCCGCTG/AGTTCCAGGCTTGTAGGTGCTG
21	Os06g0521500	CGAGAAGGAGGAAAAAGCCAA/TTGATGGCGTCGATCACGT
22	Os06g0218900	AGCAGTTAAGCAAGCCCAGACA/TTTCAGGCACAACAGAGGTCG
23	Os08g0470200	ATTTTCCGGCCTTACCCTCA/CGACAAATCCGCCGCTAAT
24	Os07g0643700	AATCCACACCGAAAAAGCG/ATTCGCGAGCTTGACGAAC
25	Os08g0353700	CGAAAAGCACCACAGGAAGAG/TGGTCTGCAGTCTTGGACTTGT
26	Os07g0678300	GCCAAATTCAAGACCGAGTTCA/TATGTCGAACGCCGTCATGTC
27	Os02g0205200	TAACGCGCATGCCTTTTGGT/CGTTACACACGCGGAAACGAA
28	Os03g0316200	TCAACTACTCCAAGCTCCGCAT/CCATCCTCTCGTACTCCACGAA
29	Os08g0540900	TTTCTGATGTGTTCGTGGAGGA/CATGATCACCACGATGACGAC
30	Os07g0468100	AGATCTACGACTTCATCGGCCT/TAGCGTAGCTAGTCGTCGATGC
31	Os05g0135400	TCATCCGCATGCATTTCCA/ATCAGTATCGACCCGTCACACC
32	Os10g0391400	ATTAACGAGGCCGTGAGGATG/CGTGCACTGTACAAATGCTGC
33	Os04g0605300	AGCATTGTTGATATGGCCGATC/TGAAGCAAACAGGCAACCAGT
34	Os06g0522300	TTGTCAACGGTTGTGATGCCT/ATCCTTCTCGCTCTCCATGGA
35	Os04g0308300	TGCATCAACCGTCAGCGTT/AGAACCGCGATGAATTCGC
36	Os02g0646200	TGACTGCATTTTGCGCTTCC/CCTCTGCGCCTCAAAAATAACG

37	Os08g0349300	CAACAACCAGCAGCCCTACAAT/GCAAATGTTTTGAACCCGTACG
38	Os06g0142200	CTGTCGTATCTGCCATTAGCCA/TTACATGCAGTGAGCCCCAGT
39	Os10g0488400	CAAGGCGGTGTGTACAGCAGTA/AAGGTCTATGTGGCCAGTGCA
40	Os07g0582400	CGGAGGTGTTCATCAACTTCG/AGCATAGTTCGAGACGTACCCG
41	Os04g0308401	TGCATCAACCGTCAGCGTT/AGAACCGCGATGAATTCGC
42	Os01g0802700	CCTGGCAGTGACATCATTTGTG/TGTGCTCATGATGTCTGCGTG
43	Os05g0334400	AAATGCTCGTGGAGACCAGCT/ACGCTTTGGAATCTCGACCTG
44	Os12g0467700	TCTGTCAAGGATAACGCCGAG/TTGTCCGTCGTATCGAGGAAG
45	Os10g0517500	CCCCGTGTTGCTGGTTTGTATA/AGCAGCTTAGCTGTGCCATTCA
46	Os03g0812400	TCCAGGAGTTCAGAGCCATGAT/CCGTCAAGTACGTCTAAACCGC
47	Os06g0702000	AGCATAGTTGCATCATGCTGC/AAAAGTCACCAAGAACACGCC
48	Os04g0308500	AACAAGGCCTACTGCGCTGTT/AATTGCTCCCAGTCACCTTGG
49	Os08g0190100	ATCACCATTGCAAATGCGGT/GGCCAAAACATCATCGGAGAT
50	Os03g0180900	TGAGGAAGACCATGGAGCTCTT/TCTTGCGTGTCTTTCAGCGTC
51	Os01g0895200	TTCGATCACGCTTGACACCAT/GCAGAGATCGCCATTGTCTCAT
52	Os04g0469100	CCGTCGATGGTCTACTGCGA/CACATGATGGAGCAGGGGCT
53	Os10g0523700	CTGCTGGCTTAGGTATGTGCAT/TCCTTCAGCTAGAAGGCATCAA
54	Os08g0360300	GGCCAATCGACAAGAGATTGG/AGTCCTGGACGTTCTTGATCCC
55	Os02g0627100	AATTCTTCGTTGCATAGCGGC/TGAAACCTGCCACTCGTACCA
56	Os08g0473900	TTGTATCCGATTGTAGCGTTCG/CCCGCAATTAACCTAGAGGCT
57	Os07g0633400	GTGATTGAGGATGGCAAGTTCA/TGTCAAGGATTTCCCGGCT
58	Os04g0365100	CAATTGCACAGGCATGCTTG/TCCACTTGCTTCATTGTTGGC
59	Os09g0325700	GCTCCGACAACATCTCTGTCGT/TCTCTCTACAAGGCGTTGCCTC
60	Os12g0227500	GAAAACGGTGTATGAGCGAACA/CGGATCGTGAGTTCAATGATTG
61	Os08g0347000	CCGACCAAGAAAGCTTCGAGTA/TGCCTCCACCCTGAACTTTATC
62	Os12g0150200	CACCTGAAGCGGCTGAACTACA/CGCAGAACTTGGAGTCGAACTG
63	Os04g0517500	CCGCTTTTTGCCTGAGAGAA/CAGCAGCACTCCCATTACACA
64	Os12g0245700	TCCTCGCAGCTGGTAATCTTG/CCGCAACACATACACAAACACA
65	Os01g0826400	CGTCGTCGTAGGAATCTCAAAG/CCCCCTAAACTACAATTTCCGT
66	Os08g0352100	CGAAAAGCACCACAGGAAGAG/TGGTCTGCAGTCTTCGACTTGT
67	Os02g0699000	GATGAGCACCACCTTCATCCA/GCACCGAGAAGATGTGCATGT
68	Os04g0589800	GCGTCCGACAAATCGTACAT/GGACCAGATCACTCGCTACACT
69	Os03g0860100	GCCACCACTCCATCTGATCTCT/GGAACCGGATCCACATGTTAAC
70	Os04g0244800	ACGTTGTCTCCGAGTTCTGCTC/GCAGTCTATCCGCACCTTCATC
71	Os12g0518200	GCATGGCAGCCCTTTTATTTAG/TCAAATAGGCCGATGTCACCTA
72	Os09g0396900	GCTGCAGGCATCCAAGTAAAT/CAAGCACGCAGCAAACGTA
73	Os05g0332600	GAGGAAGAACTTGGAAATGGCT/GCCTTTTGAACCATCTTCCAGT
74	Os01g0736600	ACGGACACATTGGTGCAATTC/CGCCTGACCAATCAAGCAA

75	Os07g0461900	GGAAACGCCCATGTGAAAGA/AGCTCGATGCCGACAATGA
76	Os10g0508700	CACACTGGTGCTGCATTGTACT/CTCAGCAAAGACAGCTGAAGGT
77	Os09g0572700	CCCTCCTCATCATGGCTTAATT/GCTCTCTCAGGATCGGAAAAGA
78	Os10g0576600	GATCCTTGCTCCATCAGTTTGC/CATCATCAAGAAGGCGCTAGCT
79	Os05g0546400	GAATACATGGTCGGCGAACTC/CTTCTTCTCGGTCTCGATGGA
80	Os07g0599500	GAGAGCCATTATCCCGAGAAGC/TTTCAGGATACGATACGACCGG
81	Os03g0131100	CTGCCTCAGGTTACCCGTTACA/ATGCTCCCATGAAGGCTGAAC
82	Os02g0581200	CCACACCCACAGCTCAACGATA/TAGTCCCCCTCGATCCAATGA
83	Os04g0608300	GGCTGTTGTTCATCCATCCAA/CAAATCATCCCTCACCATCCA
84	Os04g0597600	AGGTGAAGACGGTCGTGTCATG/ACCCCGTTCCTAACGCTACCTT
85	Os08g0508800	TGGCCGGAACAAGGATAGAAA/GCGTTTTGTCTCATCAGATGGA
86	Os04g0639000	GGAGAAAAGCCACCGAACAACT/GGCAAAGCAACGGCATCATA
87	Os03g0184300	CCACCACTGGAAGATCAACATC/CGAGGCAATCTACTCCCACTT
88	Os09g0484800	TCATGAACACCGAGGAGGAGA/GCCATTGATGCTGAACTCGAA
89	Os01g0917900	GCAGCATGGCGGTAGTAGCTAT/CTCGTACGCCATCAACCAATC
90	Os02g0198200	GAATGGAACGGTGACCTTCAAG/TCCCCACCCAACGAGTAAAAG
91	Os07g0561300	CGTTCGAATCGGAGGTTGTAGA/TGAAGCGCGTGTCCTGTAATAA
92	Os03g0738600	CACGATCATTTCATGCCCTACT/AAGTTGTCGTCGAGGCTATTGA
93	Os03g0180800	AAGCCTTGGACACATGCACTC/CAATTCTCAAATCGCAGACGTG
94	Os12g0108500	ATTGTTCGGTCACCGACATTG/CTGCAAGCCACATATGCTTGAA
95	Os09g0442100	ATCAGGACAGGCATCGACAGA/TCACGGTCATGCAGCACAA
96	Os01g0882800	CGTGCCCTACATGATCGTCTT/CAGGAGATCTGATCGAAGTCGG
97	Os10g0521400	ACGGCACCCTCCAAAAGAA/ACGACTCGTTGATCCCTTGCT
98	Os08g0398300	AGGCAGTTGATGACTCCCTGAA/CCCCACTTGCTTATTTCCAACA
99	Os09g0511300	GAGCCGTCGGTCATCGATATAA/ATGTTGAACTCCAGCCCTTCG
100	Os10g0459700	GATCCATTGTTCGCGTTCGG/TCCCAAACTTCTTTCAAACACGTAA
101	Os09g0543400	CTTCATGGCCGGCCGGACCTTC/GGCCGTCGTCGATCCAGTACTC
102	Os03g0194600	GAACCTGAGGAGCACCAAGAA/GCCTACTACGCATCAAGACGTG
103	Os08g0550200	CCATCTGCACGAACCTGTTTG/TTGAACCCGAAGAACACCATGA
104	Os03g0125100	CCGCTCGCATCTTACCATTAC/TTTTAGTGCTGCGGCGAAG
105	Os08g0472800	GCGAGCATAATCTCCTTCACGT/CCCTTTGGAATCAGGAAACCTT
106	Os11g0701100	AGCGCGTGGATGTTTCAGAA/ACCTGACGAACTGCAGCATCTC
107	Os01g0164100	TGCCACGACGTCAAGAATCA/CGCCCATGAAGAAGTCAGAGAC
108	Os07g0599300	GACCAAGCAGCCATGATCGTA/CACAAGGTGTAGAGGCCAGGTT
109	Os12g0484600	GTTCGTTGTGTCGGTGTTTGG/TCTGCGCCATGTTGTCGAT
110	Os08g0200100	TGGATCTCATGCTGCCCTAATT/CCAGAAGCTCAATACCCACCAT
111	Os01g0138500	GACGAAAGCGAATCAACGAGAA/GCTCGGATTCCAGAAACTCGA
112	Os02g0143400	TCAGGAAGGCAGAGGAGGAGTT/ATCCTCAGAGCTCCCTCATGCT

113	Os06g0142650	ATGGAGAGCAAGACGGCCAA/GGCAGTCATGGTGCAGGTAG
114	Os06g0292400	GAAAAGGCAGGTGGCAGAAA/ACCACAAATGTCTGCACGAGAG
115	Os04g0541700	AAGCAAGAGAAGCTCGCTCTCA/GCCTCTCTCTTAGCTCGTGCAA
116	Os05g0510100	TCGGTGATCGGATCAATGG/GATACATATTTTACACATCTCTAAGCATGAAC

Table S9. Sequences of primers used for qRT-PCR analysis of N starvation-repressed genes

No.	Locus	Primer sequence (5’ -> 3’)
1	Os02g0770800	TGGAGAAGATGGGCTATGAC/ACTCCGATGTACCGGAACTC
2	Os05g0114400	AGTTCGCCATTGGCCAGGCTCTC/GTGGATGGTTCAGGTCGAGCCACAG
3	Os08g0468100	CATAGGCCATGATTTCCCTTTC/TACAGGAAGGAATCAACCGCTA
4	Os11g0184900	CGGCGAGCGAGGTTAAAAAT/CCCATCCGGCAGAAACAAA
5	Os01g0631200	TCGTTTTTGTTTACCCGGCA/TCGTGAATCAGGAGAGCCTCTG
6	Os03g0684700	GCAGTCTGCGATGTGCTTTTC/TGTATTTCTTGGCAGCAGGTGA
7	Os08g0120600	CTCGCAATGTCGCGTATTTG/CTCGCTAAACAAGACCAAGCAA
8	Os05g0194900	CCGCCTGAAGCTTGAAGAA/CCAAACATGGCCGTGTGAT
9	Os03g0609500	TGCGAGCTGGACCTCTGCCT/TCAGCTGAAGAGGTTCAGCA
10	Os09g0482800	TGTCCTCTTGCTGAAATGGCAT/CCATCCCGGATTACATAGCTGA
11	Os03g0126900	TCGGTGTTCTTTCTTATCGTCG/CCATTCTGATCACATTCCGATC
12	Os08g0113900	TGTCCATGGTGGTAATCGACAA/CCGTGTACCGTAAGTTGCTGCT
13	Os01g0860601	ACTGCGTCATCTACACCCACAA/CGCGATCGACATGGACTCTAT
14	Os04g0506800	TCATTGCAACAAAGCGAAGC/GACCCAAATGTTCACGGATTG
15	Os09g0484900	GGAACGCCCCTGAAAATTGT/TGGCAGTAGGATGGTGAGAGCT
16	Os07g0589000	TCCCTTAGCCATTTTTTGGC/TCAAGTAGAAGCAGTGCACGC
17	Os12g0198900	ATTTGGAGAGTGCCCAGCATC/CACGAAGCATTCGCATCTCA
18	Os04g0665600	AAGCTGATGAATTCGGATGGC/TGGAGATGGCTCTTGATGTGG
19	Os07g0147500	TCTAGTTGGCGGCGCTATTCT/GCTTGGTCTGCTGCACTTAGCT
20	Os05g0119000	CGCCGACTAATCTTCCCTCTT/CCATAAT GAATCAAAATGCAAGGT
21	Os05g0360400	AAAGGTTGGTGTCGGTGATGAC/AGCAAAACCTCGCGTCTTCTC
22	Os09g0545280	AAGGCAACCTACGCTGCTCAT/TGATGCAGTGGCCCTTATCTG
23	Os01g0179600	TCGCGCCAATTAGATGTGTC/TGGAGCATCATCCCAATTCA
24	Os09g0433800	AGAAGCTTTCCAGGTCTCATGG/GCACATAAGCAGGAGCAGGTTA
25	Os02g0620600	CATGTCTAGTGGAACCGGAGGA/CAATCACGAAACCTGCAGCAT
26	Os02g0756600	CAACGGCCAGAGCAAGAAAAC/CATGGAGCAGTTGATGTCGGA
27	Os02g0120100	CCAAGGTTGCTTCTGGATCAAA/TGCAGTATGATCCACGGAACAG
28	Os02g0325600	GAGTGCATCACATCAAAGTGCA/TGCGATCACGAAGCAATCA
29	Os11g0113700/ Os12g0113500	TCTGAAGGACATTGTCTTGGCC/TGCGACTGCTGCTATTCATCTC
30	Os10g0578800	AAAGCTTGTAAGGAGGCATGCA/CGAGAATGTTGATGCCACAGC
31	Os01g0908200	GCATGCAGTTCAGGAGAAAGGA/GCCCCCACTTATCGTCATCAT
32	Os05g0111800	AGCACTTGGATGATCCTGGCT/CAAACGCTCTTGTCACCTTCAA
33	Os11g0305400	CCAGGATTGCGTGAAAATCCT/CTGCCTCTCGTGATGGAATAGC
34	Os03g0764600	GGTAGTGTTGGCCATGTATA/GCGAAAATTTGGGGTCTGCC
35	Os05g0401500	TCCCTTTTTGGTTGCGAGAA/CAGCAGGACATGAACATCCATC

36	Os05g0443700	AAGTAGCCTGGACCAGCACTGA/CACGTGGCAGTTCTGAACAAAA
37	Os05g0472400	ACAGTTGCTCAGAGCGATGTCA/TACCGGAATCCCGTTCAAAAAC
38	Os04g0280500	GTCATCATCCACCTCCAGCATT/GCGGCATAAGATGGTTGCAT
39	Os05g0506800	CCCATCGAAATTGGCAAAGA/GCACACCGTAGAATTGTCATCG
40	Os06g0535200	AGGCCAGTTTTTCTTGCCACT/CGCGTTAGCATCTACAAACAGC
41	Os06g0566300	TGCTGATTTTCTTAGCCGGAAG/TACGAGCCCACTTGAAGCCTT
42	Os05g0380250	GTGAATCCATCGGCAGGTTCT/GCAATGGCCTGTTGATTGGT
43	Os01g0208700	ATGGCCTTGCCGGTTAAAA/TCAATCCACTCCCACCAATCTT
44	Os07g0686300	TGATGTTCATGGCGTACCTGC/ACACGCCCTTAATGGCTAGAGC
45	Os02g0214900	AACCGATGGCAGAGGATCTTG/ATTTGCATCCGCAGTAGGAGC
46	Os01g0179800	TGCATCGAATCAGCAAAGCA/CCCCTTTTGCATGTTCAGCA
47	Os03g0784700	AGCAGAAAAACAAGAACGCTGG/TCCTCGATCTTGTCCTGGACA
48	Os03g0445700	TCGTGCGACCTAGGCCTGTG/CCGGCCTCAGACAAAAAGGTT
49	Os04g0649500	CGTCCTCTCCGATTGGTTTCT/CACTTCCCCACTGACAAAGTGA
50	Os04g0649600	TTGATGCTCTGCTCTTGGTGTT/TGATACGGTGAACCTCGCAA
51	Os04g0640900	CAAGCTCCTGATACCGCATCA/CCTTTGCCATGCGAAGAAGAT
52	Os04g0475600	TTTGGCTGGAGGAGATGATG/TCACAAACTCCACCTTCCAGTT
53	Os12g0204100	TCAATGAACCCTGCAAGAACAC/AGCCCCTGTTCCAGCAATT
54	Os02g0807000	CCCGAGTTTGTCCGATGTACA/CAGTTCAGCCAAAAGGTGATCA
55	Os04g0520700	GATGCAAGAGAGGACAGGAAGA/CTCCAGGTGCCCTCAAAATT
56	Os06g0633100	TTTTCCACCCCTGTAATCATCG/TTGACCTCTCGTCTCCTCTCCA
57	Os06g0683800	TGGTTCGCTTACTCCACAAGCT/TCGCAATCTGCCAAGCAAC
58	Os05g0472700	GTGCGGCGTTTCAATTTGTT/TGATCAGACGCGCCTAAGAAC
59	Os03g0823400	GGTTTCAGGTTTATGTGCTGGA/AGCAGAGCGAGGAGATGAAAA
60	Os02g0765600	ACCTGCGATTTTTACCCTGACC/CGTGGACAATTGCATCCGTAAT
61	Os05g0501600	TGGATGGAGCACCCTACACTGA/GGCATCTTTGCAGTGACTTGGT
62	Os01g0803300	GCGGACTGCAAGAAAGCAAAT/CACTAGCTCCTTGCTCGTTGGA
63	Os01g0383100	GCCCCAGCAATTGTTCGATA/ACAGGATCCGTGACCTTCCAT
64	Os11g0484500	AAGATTTGCAGCTACGCGCA/TCCACACTCTTTGCTCGCAGA
65	Os11g0256900	TGCGAGCTGCTTGACATAAGTG/CGGTTCTCATCCATCTCGAAGA
66	Os06g0692600	CGGCGATACAGGACATTGTTAG/AGCCGATGAATTCGACGTTGT
67	Os01g0357100	GGGGCCGTGCGGAGGGAGAG/ATGGGCGAAGGCATATTGTC
68	Os05g0443500	GCTGATTTCAAAGTGGACTCGC/TGGCCATTTTTTGGATAGCG
69	Os04g0683700	TTCAAGGTGCCCAAGAAGGT/GGCCGTCTTGGGAAGCTC
70	Os03g0599000	AC ACTCAAGGTTTGGCGATACC/TGACCTGGCCACCAAATTG
71	Os04g0561500	GAACATGACGCCCCATTTCA/AACCCGAGATTCCGCATGA
72	Os07g0406300	TGCCATTGAAGGAGAGCGAA/GGCATCCAACTCATCAGAACG
73	Os04g0403701	CCAATTGGTGATCCTGCAAAGT/TGCTTCCTTCGCTGCTTTAAGT

74	Os01g0621900	TGATCTGTTTCCAAGAGTT/GACAGAAAGCCCGGTACTCC
75	Os03g0190300	CTTGGCTGATCAATCCATTCGT/CAATCCCTTAGGCAATGATCCA
76	Os08g0207500	TCATGGCGACTTTCTTCTCCC/AGCTGGATGAGATTGCGATCC
77	Os08g0465700	CCGTTGTGGTCAGCTAGTTGCT/ACGTACGCGCCACAATTCA
78	Os03g0838900	GAGGACATTCTCCGCTGATCAA/TGTTGTCACCAGCATCGACAA
79	Os04g0165200	GACACGTGGCAGATAACTCCAA/TGGTTAGCGGTGTGTGGTTTTA
80	Os09g0453300	CAAGTCGCACCACGATGAACT/CCTGGCGTCACATTTTGCA
81	Os02g0756200	TGTAATCCATAGTGCCGGACAC/CAACTTCCCAAGCGTACAGGTA
82	Os03g0854000	ACGCGTGCTACCTAACCTACCA/GCGATCAGCGATAGCCTGATAT
83	Os01g0355100	CGCCACTGTTGTTGGTTTCTTT/TAGACGCCAACTGCATCAAGG
84	Os10g0554200	GGCCACTAATAGTGACCGATCG/GCTAGCTTGCGTATGTCATGCT
85	Os10g0328400	CGTCGTTTGATTTGGATACGTG/TCACCACAGAGCAACTCGTCA
86	Os06g0323100	GTCTGTTCCCGGAGCGATCTAT/GCAGACAGAAGGTGGAGGTGAA
87	Os07g0119300	GGAGGATGGCAGGTCTGAGA/CTCGATCTCACTGTATACAA
88	Os02g0306401	TCGCTCTTCGGAAATTGGA/TGCTAGACACCACACTGTGCAT
89	Os09g0474000	AACCTATCCAGGAATTGCTCCC/CCATCTGGTAGGCGTCATCTGT
90	Os03g0243100	GAGTGGCAGCGGATTAGTGAA/TGGAATTTCTACGTCACCGACA
91	Os01g0747300	GAGTAAGATCGCCATGTCGGTC/GCTCCCGCAGAACACAATACA
92	Os02g0525100	GAAATTCTCTCGTGCTGCTCAA/CTCCAAAAATATTGGTGCCTCC
93	Os01g0191700	GCCATGACACCGGCAAAATA/CCTGGTTAATACCGCGATCCTT
94	LOC_Os03g12690.1	GGGTGGAGAAGATATCCATGCT/ACACGCTCGGAAGTCGCT
95	Os05g0342000	TCACATCCCACCAATTGTTCCT/TGGTGGAGTGTGCGCTAGAGTA
96	Os01g0888900	CGTGTTTTGGTCGAATTCAAGG/AAATTTACTCTCCACCCCGCC
97	Os04g0645500	TATCGTCTTTTTCGCCGGACA/CGTTGGATTGGAGACGATGGA
98	Os05g0411100	AACGATTTCTGCGACCGGT/TGATCAGGTTGGCATTGAACC

		Primer sequences of reference genes used for qRT-PCR analysis
99	*UBC3* Os02g0634800	AAGGCATGGCTGATTCATGT/CAGATGTTCACATGGTGACAGTA
100	*UBQ10* Os02g0161900	TGGTCAGTAATCAGCCAGTTTGG/GCACCACAAATACTTGACGAACAG
